# Synthesis of new tricyclic 5,6-dihydro-4*H*-benzo[*b*][1,2,4]triazolo[1,5-*d*][1,4]diazepine derivatives by [3^+^ + 2]-cycloaddition/rearrangement reactions

**DOI:** 10.3762/bjoc.14.155

**Published:** 2018-07-18

**Authors:** Lin-bo Luan, Zi-jie Song, Zhi-ming Li, Quan-rui Wang

**Affiliations:** 1 Department of Chemistry, Fudan University, 2005 Songhu Road, Fudan University, Shanghai 200438, People’s Republic of China

**Keywords:** 1,4-benzodiazepine (BDZ), cyclization, hydrazones, oxidation, rearrangement

## Abstract

Two new series of tricyclic heterocycles, namely 5,6-dihydro-4*H*-benzo[*b*][1,2,4]triazolo[1,5-*d*][1,4]diazepinium salts **10** and the related neutral, free bases **13** were synthesized from 4-acetoxy-1-acetyl-4-phenylazo-1,2,3,4-tetrahydroquinolines **8** and nitriles **9** in the presence of aluminium chloride by the [3^+^ + 2]-cycloaddition reaction of the in situ generated azocarbenium intermediates **14** followed by a ring-expansion rearrangement. In the rearrangement reaction, the phenyl substituent in the initially formed spiro-triazolium adducts **16** underwent a [1,2]-migration from C(3) to the electron-deficient N(2). This led to the ring expansion from 6-membered piperidine to 7-membered diazepine furnishing the tricyclic 1,2,4-triazole-fused 1,4-benzodiazepines.

## Introduction

**Figure 1:**
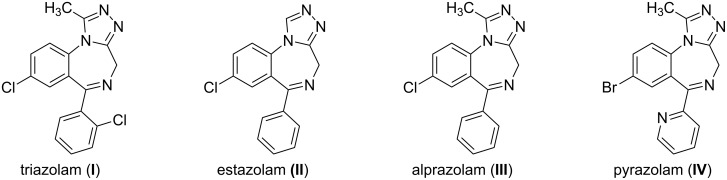
Examples of marketed pharmaceutical 1,2,4-triazolobenzodiazepines.

Heterocyclic compounds comprising a 1,4-benzodiazepine (BDZ) ring have been a topic of continued interest as they exhibit a wide spectrum of drug-like profiles such as good anticonvulsant activity against acutely elicited seizure, particularly for the central nervous system [1–5]. Many biologically active small molecules with such a core structure have been marketed for the treatment of various diseases, mostly as psychotropic substances [6]. Thus, for example, chlordiazepoxide (Librium) and the benzodiazepine diazepam (Valium) have been a sedative and hypnotic medication and marked for the treatment of anxiety, insomnia and withdrawal symptoms from alcohol and/or drug abuse by Hoffmann-La Roche since the 1960’s. Benzodiazepines are thus categorized as a privileged heterocyclic system that is the structural basis of a large number of drugs as defined by Evans about 30 years ago [7–12]. Later, more detailed research revealed that improved biological activities, metabolic stability and other profiles could be achieved when a third heterocycle, especially a 1,2,4-triazolo moiety, was attached to the seven-membered ring as part of 1,4-benzodiazepine [13–14]. Among various reported 1,2,4-triazole-annulated 1,4-benzodiazepines, triazolam (**I**), estazolam (**II**), alprazolam (**III**) and pyrazolam (**IV**) are prominent examples of such clinically drugs having enhanced effects on the neurotransmitter γ-aminobutyric acid (GABA) at the GABA_A_ receptor and low toxicity (Figure 1) [15].

Although 1,4-benzodiazepines are widely prescribed medicines, side effects like drowsiness, drug resistance, addiction and withdrawal potential are detrimental [16]. Consequently, the development of expedient synthetic protocols to access new members of 1,4-benzodiazepine derivatives has long been the subject of considerable interest aiming at the discovery of biologically active and drug-like compounds [17–30].

Over the past years, we have been engaged in the synthesis of novel 1,2,4-triazolo heterocycles annulated to benzoazepine or heteroazepine derivatives by a tandem [3^+^ + 2]-cycloaddition/rearrangement between 1-aza-2-azoniaallenium ions with nitriles [31–34]. In the [3^+^ + 2]-cycloaddition, α-acetoxyazo intermediates are initially transformed into an azocarbenium ion. Subsequently, it takes part in a cationic cycloaddition reaction with the triple bond of a nitrile followed by a rearrangement reaction [35–39]. This type of ionic cycloaddition–rearrangement protocol proved to be quite general and has also been conducted intramolecularly for a tethered olefin moiety by Brewer and co-workers in recent years [40–42]. In the present work, motivated to achieve structural diversity and potential biological profile improvement of 1,4-benzodiazepines, we wish to describe the synthesis of unprecedented tricyclic heterocycles, i.e., 5,6-dihydro-4*H*-benzo[*b*][1,2,4]triazolo[1,5-*d*][1,4]diazepinium salts **10** and the related neutral free base derivatives **13** via the cationic [3^+^ + 2]-cycloaddition/rearrangement reactions using the bicyclic 4-acetoxy-4-azo-1,2,3,4-tetrahydroquinolines **8/12** as the key starting material.

## Results and Discussion

**Scheme 1:**
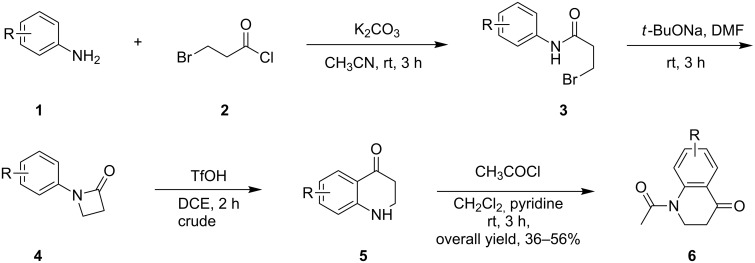
Preparation of *N*-acylated 2,3-dihydro-4(1*H*)-quinolones **6**.

The required *N*-acylated 2,3-dihydro-4(1*H*)-quinolones **6** were generally prepared following a literature method with the acid-catalysed Fries rearrangement of *N*-arylazetidin-2-ones of the general form **4** [43–44]. As illustrated in Scheme 1, the preparation starts from the related anilines **1** which were acylated with 3-bromopropionyl chloride (**2**) to afford amides **3**. Upon basic treatment with *t*-BuONa, the amides **3** were converted to the cyclized β-propiolactams **4**. In the presence of triflic acid, the Fries rearrangement occurred smoothly to yield the dihydroquinolinones **5**, which were then converted to 1-acetyl-2,3-dihydroquinolin-4(1*H*)-ones **6** by reaction with acetyl chloride in 36–56% overall yields (Scheme 1, see Supporting Information File 1 for details).

**Scheme 2:**
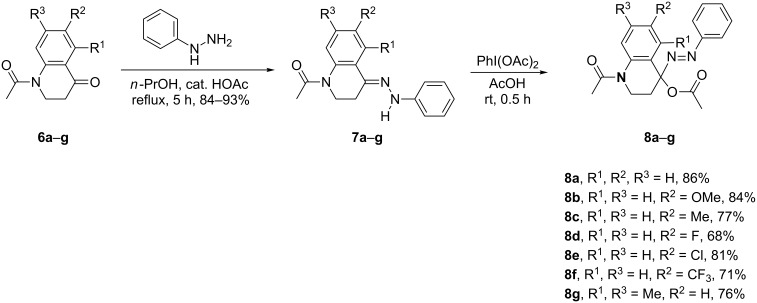
Synthesis of α-acetoxyazo compounds **8a**–**g**. Reaction conditions: for synthesis of **8a**: **7a** (10.42 mmol), PhI(OAc)_2_ (12.50 mmol), AcOH (10 mL); for synthesis of **8b**–**g**: **7b**–**g** (0.71 mmol), PhI(OAc)_2_ (0.85 mmol), AcOH (1 mL). ^b^Isolated yield. ^c^Ratio of E/Z isomers was not determined.

The phenylhydrazones **7** were readily acquired by condensation of the quinolones **6** and phenylhydrazine with a catalytic amount of AcOH in refluxing *n*-propyl alcohol. Subsequently, the hydrazones **7** were converted into the 4-acetoxy-1-acetyl-4-phenylazo-1,2,3,4-tetrahydroquinolines **8** via the oxidation with hypervalent iodine(III) reagent PhI(OAc)_2_ (Scheme 2) [45]. The electron-withdrawing *N*-acetyl functionality in compounds **8** was introduced to conceal the basicity of the N(1) atom, thus avoiding the plausible disturbance in the following cationic polar cycloaddition/rearrangement reaction.

After the accomplishment of the synthesis of key intermediates **8**, we tried to apply the well-documented protocol in this laboratory for the synthesis of the target tricyclic heterocycles [46]. Thus, the α-acetoxyazo compound **8a** was allowed to react with acetonitrile in the presence of AlCl_3_ as a Lewis acid at low temperature (−40 °C) in dry CH_2_Cl_2_ for a period of 0.5 h. Then the reaction mixture was gradually warmed to room temperature and kept at this temperature for an additional hour. The work-up afforded successfully the 5,6-dihydro-4*H*-benzo[*b*][1,2,4]triazolo[1,5-*d*][1,4]diazepinium salt **10a** as a white solid in 81% yield. It is noteworthy to mention that the counter ion in the product had been changed from anion AlCl_3_(OAc)^−^ to picrate anion. This variation proved to be quite beneficial for the formation of stable salts.

**Scheme 3:**
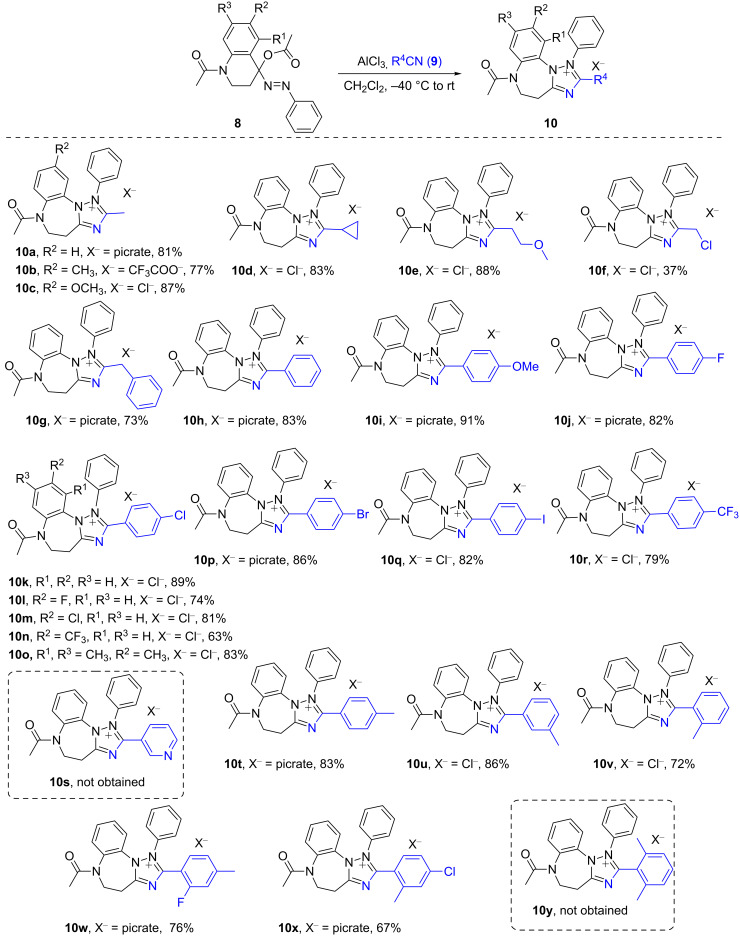
Synthesis of tricyclic benzo[*b*][1,2,4]triazolo[1,5-*d*][1,4]diazepinium salts **10**. Reaction conditions: substrate **8** (0.25 mmol) in CH_2_Cl_2_ (2 mL), nitrile **9** (0.35 mmol), AlCl_3_ (0.35 mmol), CH_2_Cl_2_ (5 mL), −40 °C for 0.5 h, then room temperature for 1 h under an atmosphere of N_2_. ^b^Isolated yields.

Encouraged by the above success, we then examined the protocol’s efficacy by applying different kinds of nitriles. As can be seen from Scheme 3, most of the expected tricyclic benzo[*b*][1,2,4]triazolo[1,5-*d*][1,4]diazepinium salts **10** were produced readily from the corresponding acetoxyazo compounds **8** and the corresponding nitriles **9**. Moderate to good yields of **10a**–**g** were obtained with aliphatic nitriles, except for 2-chloroacetonitrile which gave a low yield (37%) of **10f** from the reaction with **8a**. This type of reaction worked also well with 3-methoxypropanenitrile to provide salt **10e** in 88% yield. Similarly, 2-phenylacetonitrile participated in the reaction smoothly to give **10g** in 73% yield.

As mentioned above, with the purpose to obtain stable tricyclic 1,2,4-triazolodiazepinium salts **10**, the anion AlCl_3_(OAc)^−^ in the initially formed products was replaced with a suitable counter ion such as chloride, trifluoroacetate and picrate, which proved to be a useful way to facilitate the isolation of the target salts **10**.

In order to further explore the scope and generality in view of nitriles, the strategy was also probed with aromatic nitriles **9** that were reacted with **8** under the same reaction conditions. The nucleophilicity of the N atom in aromatic nitriles should be lower than that of aliphatic ones owing to the conjugation of the triple bond with the benzene ring. To our delight, compound **8a** reacted smoothly with benzonitrile to give the 2-phenyl-substituted benzo[*b*][1,2,4]triazolo[1,5-*d*][1,4]diazepinium picrate **10h** in 83% yield. Then the effect of the electronic nature of benzonitriles was examined with benzonitriles carrying different functionalities. It was disclosed that various electron-donating and electron-withdrawing groups on the benzene ring were compatible with the reaction conditions furnishing the desired products **10i**–**r** in good yields. This indicated that the nucleophilicity of the nitrogen atom of nitrile **9** is strong enough to override the electronic effect of the substituent in the benzene ring. However, when nicotinonitrile was employed, the reaction was unsuccessful, and the expected product **10s** could not be identified in the reaction mixture. We speculate that this could be due to the stronger electron density at the N atom in the pyridine ring that interferes with the action of the Lewis acid.

By employment of **8a** as a model substrate, we next turned our attention to determine possible steric effects with *o*-, *m*- and *p*-methyl-substituted benzonitriles. The tested reactions proceeded well to give the products **10t**–**v** with comparable good yields, respectively. This indicated that there was no obvious steric impact on the reaction with monosubstituted benzonitriles. On the other hand, from disubstituted benzonitriles including 2-fluoro-4-methylbenzonitrile and 2-methyl-4-chlorobenzonitrile, the tricyclic benzo[*b*][1,2,4]triazolo[1,5-*d*][1,4]diazepinium salts **10w** and **10x** were obtained in moderate yields of 76% and 67%, respectively. However, one limitation was observed for the reaction between **8a** and 2,6-dimethylbenzonitrile in which the cyano group is flanked by two substituents in the *ortho-*positions. Under the same conditions, the reaction failed to give the desired product **10y**. This result could be attributed to the increased steric hindrance in nitrile moiety.

**Scheme 4:**
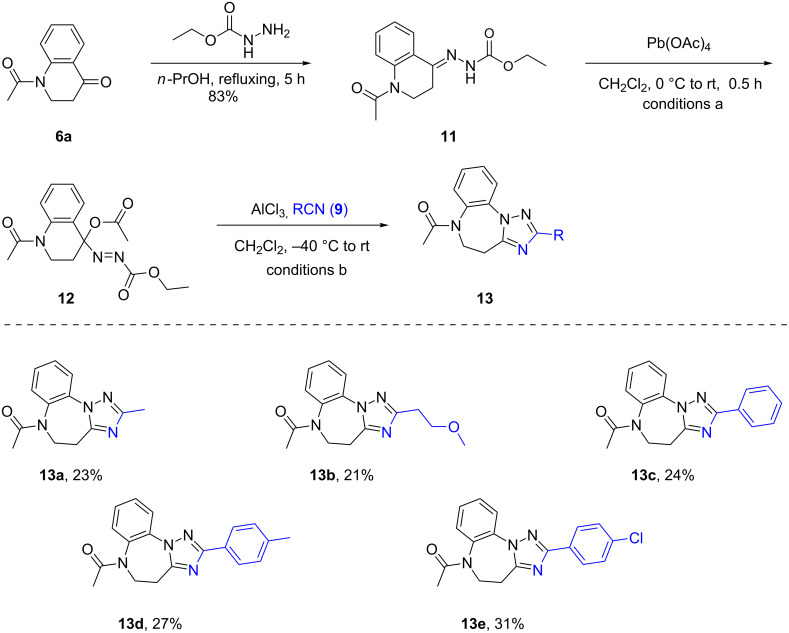
Synthesis of N(1)-unsubstituted benzo[*b*][1,2,4]triazolo[1,5-*d*][1,4]diazepines **13**. Reaction conditions: a) compound **11** (1.20 mmol), Pb(OAc)_4_ (1.44 mmol), CH_2_Cl_2_ (10 mL) to give the crude compound **12**. b) (i) compound **12** (1.20 mmol, calculated via theory yield), nitrile (1.68 mmol), AlCl_3_ (1.68 mmol), CH_2_Cl_2_ (5 mL), −40 °C for 0.5 h, then at rt for 1 h under N_2_; (ii) ice water (1 mL); 0 °C, 5 min. Isolated yields are given.

As can be seen from Scheme 3, equally satisfactory results could be obtained from the reaction of other acetoxyazo compounds **8** with several different substituents on the benzene ring, as exemplified by the synthesis of **10b**,**c**,**l**–**o**. These results demonstrated the broad scope of the present strategy for the preparation of novel tricyclic heterocycles **10**. Based on the success in the synthesis of the triazolobenzodiazepinium salts **10**, we then turned our efforts to access the N(1)-unsubstituted neutral tricyclic heterocycles. To this end, the phenylhydrazones **7** were replaced by the ethoxycarbonylhydrazone **11**, in which the ethoxycarbonyl group was hoped to be removable by hydrolysis [34]. As presented in Scheme 4, ethyl carbazate was used to prepare the hydrazone **11** by condensation with 2,3-dihydro-4(1*H*)-quinolone **6a** [46]. However, it was odd that the oxidation using the hypervalent iodine(III) reagent PhI(OAc)_2_ as described for phenylhydrazones **7** failed to produce the expected α-acetoxy-ethoxycarbonyl compound **12**. Instead, the hydrazone **11** remained intact and was recovered. Therefore, we switched to a stronger oxidant, Pb(OAc)_4_. To our pleasure, hydrazone **11** was successfully oxidized to provide the required azoester **12**. However, NMR analysis revealed that compound **12** was impure and contained also some cyclized byproduct [47]. Furthermore, it was discovered that compound **12** was quite unstable and tended to degrade when it was subjected to chromatographic separation. Based on this, we planned to use the product mixture resulting from the oxidation of hydrazone **11** directly for the following reaction with nitriles **9** in the presence of AlCl_3_. We were pleased to observe that the reaction proceeded as expected under the usual reaction conditions and the desired products **13** were obtained after quenching with H_2_O at 0 °C. As can be seen from Scheme 4, both aliphatic and aromatic nitriles worked similarly.

**Scheme 5:**
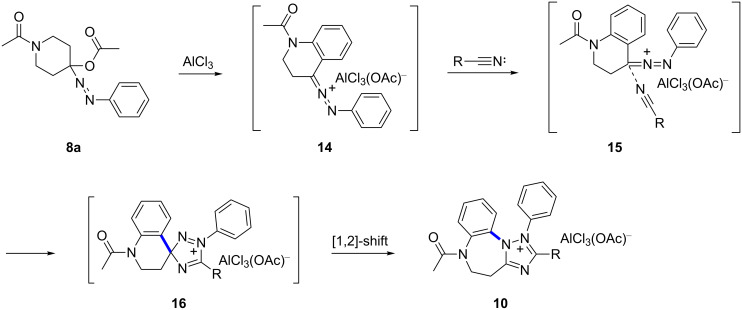
Mechanistic rationale for the [3^+^ + 2]-cycloaddition/rearrangement reaction.

In light of the experimental achievements and preceding theoretical work, we rationalized the synthesis with a proposed mechanism as outlined in Scheme 5 with the α-acetoxyphenylazo compound **8a** serving as a model substrate. In this pathway, the reaction is initiated by AlCl_3_ coordination with the acetate moiety of **8a** to generate the azocarbenium ion **14** as a reactive intermediate, which cannot be isolated by now [31]. Then, the nitrogen atom of the nitrile approaches to the central electron-deficient carbon atom in **14** to form a Ritter-type nitrilium salt **15** [48]. Salt **15** then undergoes a concerted but asynchronous cyclization [49] to afford the initial spiro-substituted adduct **16**, which has a strong proclivity for ring expansion to occur. Accordingly, the 6-membered piperidine ring was enlarged to the 7-membered diazepine ring giving the isolated benzo[*b*][1,2,4]triazolo[1,5-*d*][1,4]diazepinium salts **10** via [1,2]-shift. It is noteworthy that the intermediate products **16** bear a diazenium function where the electron-deficient N(2) displayed the feature of a latent nitrenium ion. The subsequent [1,2]-shift after cationic Huisgen-type cycloaddition occurs with complete regioselectivity to N(2) not to N(4). Meanwhile, there are two possible migrating moieties: the aromatic side competes with the aliphatic side. It has been reported that the migratory tendency of substituents prefer those with a higher ability to accommodate the respective carbocations [46]. As anticipated, it was the phenyl moiety not the aliphatic moiety that moves from C(3) to N(2) to furnish the isolated products. This rearrangement falls into the uncommon class of migration of a substituent from a carbon atom to an electron-deficient nitrogen atom [50].

**Figure 2:**
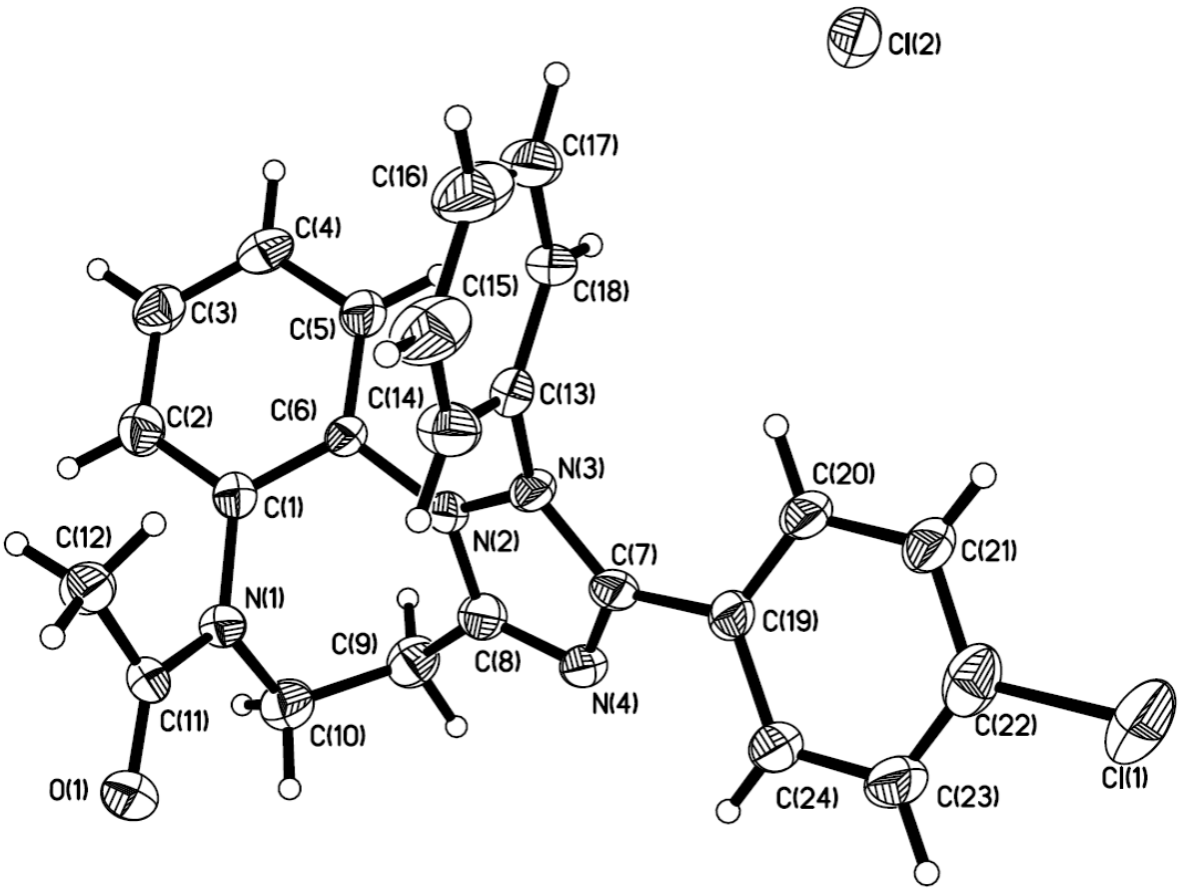
Crystal structure of salt **10k**. The displacement ellipsoids are drawn at the 30% probability level.

**Figure 3:**
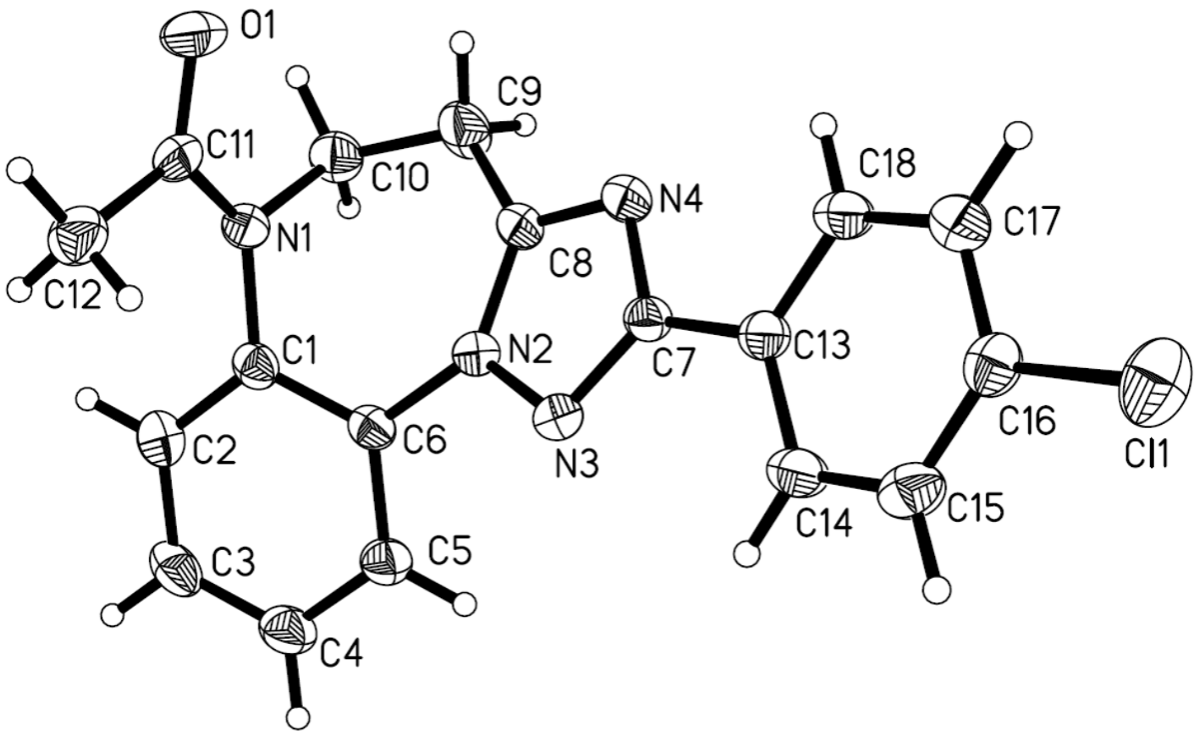
Crystal structure of the free base **13e**. The displacement ellipsoids are drawn at the 30% probability level.

The assignment of the structures for all of 5,6-dihydro-4*H*-benzo[b][1,2,4]triazolo[1,5-*d*][1,4]diazepinium salts **10** and the free base counterparts **13** was made on the basis of spectroscopic analysis. To further support our assignment, we were able to acquire X-ray crystal structures of **10k** as well as of **13e**, which indisputably confirm their structures [51]. For these two compounds, the ORTEP pictures are shown in Figure 2 and Figure 3, respectively.

## Conclusion

In summary, an appealing series of 1,2,4-triazole-fused 1,4-benzodiazepines **10** and **13** were prepared via the [3^+^ + 2]-cycloaddition reaction followed by a cationic [1,2]-rearrangement reaction. The procedure is general and has several advantages such as ready availability of starting materials, good flexibility in terms of substitution, and an unprecedented fusion pattern of the produced heterocycles. In view of the fact that the constructed 1,2,4-triazolobenzodiazepines represent a class of N-containing fused heterocycles with a new type of scaffold that is biologically interesting, the present synthetic protocol paves the way for further applications in drug-discovery research.

## Supporting Information

File 1 Experimental procedures, characterization data, copies of NMR spectra and X-ray crystal data of compounds **10k** and **13e**.
