# Supplementary material for: Synthesis of new tricyclic 5,6-dihydro-4H-benzo[b][1,2,4]triazolo[1,5-d][1,4]diazepine derivatives by [3+ + 2]-cycloaddition/rearrangement reactions
Source: Beilstein J Org Chem. 2018 Jul 18;14:1826–33. doi: 10.3762/bjoc.14.155 (PMC6071722; doi:10.3762/bjoc.14.155)
Supplement: File 1 — Experimental procedures, characterization data, copies of NMR spectra and X-ray crystal data of compounds 10k and 13e. [file Beilstein_J_Org_Chem-14-1826-s001.pdf]

Supporting Information

for

Synthesis of new tricyclic 5,6-dihydro-4*H*-benzo[*b*][1,2,4]triazolo[1,5-*d*][1,4]diazepine derivatives by [3$^+$ + 2] cycloaddition/ rearrangement reactions

Lin-bo Luan, Zi-jie Song, Zhi-ming Li, Quan-rui Wang*

Address: Department of Chemistry, Fudan University, 2005 Songhu Road,

Fudan University, Shanghai 200438, People's Republic of China

Email: Quanrui Wang - qrwang@fudan.edu.cn

* Corresponding author

Experimental procedures, characterization data, copies of NMR spectra and X-ray crystal data of compounds **10k** and **13e**

1. General Information S2
2. Experimental procedures and characterization data S3
3. Copies of NMR spectra S37
4. X-ray Crystal for compound **10k** S87
5. X-ray Crystal for compound **13e** S91
6. References S95

Experimental procedures and analytical data for new compounds

1. General information

Reagents and starting materials, including anilines, nitriles and hydrazines, were supplied by Aldrich, Acros, Lancaster, Alfa Aesar, and TCI at the highest commercial quality and used without prior purification. CH_2Cl_2 was freshly distilled from CaH_2 before use [1] and the other solvents were used as received from commercial suppliers. TLC analysis was carried out using silica gel on glass plates. Silica gel flash column chromatography was performed on Biotage using a pre-packed silica gel column, a detector with UV wavelength at 214 nm and 254 nm. Reverse flash column chromatography was performed on Biotage using C_{18} (100 g) column, a detector with UV wavelength at 214 nm and 254 nm. ^1H NMR (400 MHz) and ^{13}C NMR (101 MHz) spectra were recorded on a Bruker Avance 400 MHz spectrometer using $CDCl_3$, CD_3OD or DMSO-d_6 as the solvent. Chemical shifts, δ, are reported in ppm relative to TMS (^1H) or to the solvent peak (^{13}C). Coupling constants, *J*, are reported in hertz. Standard abbreviations are used to explain the multiplicities. High-resolution mass spectra were obtained with a Bruker Micro TOF 11 spectrometer at the positive electrospray ionization (ESI) mode. Melting points were measured using Büchi apparatus B-540 and were uncorrected.

2. Experimental procedures and characterization data

2.1 Procedure and characterization data for 1-acetyl-2,3-dihydroquinolin-4(1*H*)-ones 6

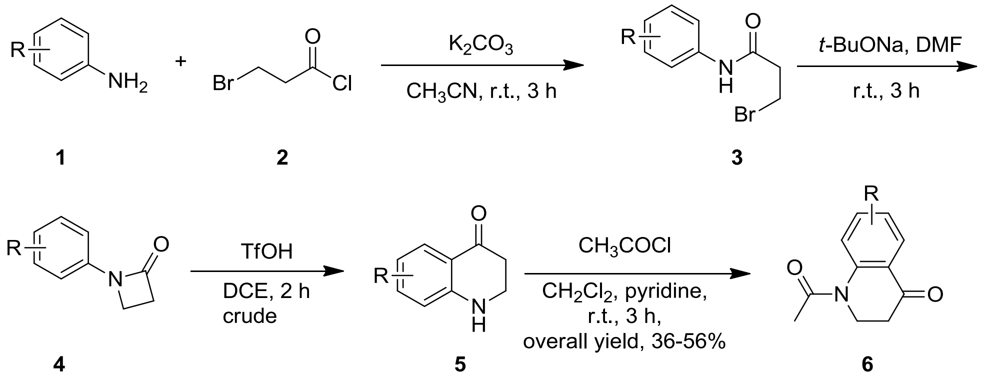

Synthesis of 1-acetyl-2,3-dihydroquinolin-4(1*H*)-ones (6a)

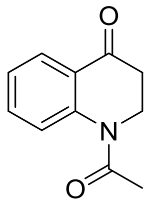

To a solution of aniline (4.3 mL, 4.40 g, 47.25 mmol) and $NaHCO_3$ (4.37 g, 51.98 mmol) in CH_3CN (50 mL) at 0 °C was added dropwise 3-bromopropanoyl chloride (**2**, 8.91 g, 51.98 mmol). The mixture was stirred at 0 °C for a couple of minutes and allowed to warm to room temperature and kept at this temperature for another 3 h. Then the reaction was quenched with water and extracted with EtOAc (20 mL × 3). The combined organic layers were evaporated, and the residue was grinded with petroleum ether/EtOAc (5:1) to give 3-bromo-*N*-phenylpropanamide (**3a**, 8.41 g, 78%) as a brown solid [2]. This solid was then dissolved in DMF (10 mL) and added slowly into a solution of *t*-BuNa (3.90 g, 40.56 mmol) in DMF (70 mL). The resultant mixture

was allowed to warm up to room temperature gradually. The reaction was stirred at room temperature for 3 h, quenched with water and extracted with EtOAc (20 mL × 3). The organic layers were collected, the volatiles evaporated under reduced pressure, and the residue was grinded with MTBE to give 1-phenylazetidin-2-one (**4a**, 3.53 g, 65%) as a red solid [2].

To a solution of 1-phenylazetidin-2-one (**4a**, 3.53 g, 23.97 mmol) in DCE (40 mL) at 0 °C was added TfOH (4.67 mL, 52.77 mmol). The mixture was allowed to warm up to room temperature and stirred for 2 h. The reaction was quenched with aq. $NaHCO_3$ and extracted with EtOAc (15 mL × 3). The combined organic layers were separated, washed with brine, dried over Na_2SO_4, filtered, and concentrated to give the crude 2,3-dihydroquinolin-4(1*H*)-one (**5a**, 3.53 g, calculated as ~100%) as a yellow solid, which was used for next step directly without further purification [2]. The crude 2,3-dihydroquinolin-4(1*H*)-one (**5a**) was dissolved in CH_2Cl_2 (20 mL). Then, pyridine (2.68 g, 33.29 mmol) was added followed by the dropwise addition of acetyl chloride (2.24 g, 28.54 mmol) at 0 °C. After completion of the addition, the reaction was allowed to warm to room temperature and stirred for 3 h. Then the solvent was removed under reduce pressure, and the residue was partitioned between EtOAc (30 mL) and brine. The organic layer was separated, dried over $MgSO_4$, filtered, and concentrated. The crude product 1-acetyl-2,3-dihydroquinolin-4(1*H*)-one (**6a**) was purified by flash column chromatography on silica gel with EtOAc/petroleum ether (boiling point range

60–90 °C) (v/v 1:3) as eluent to afford the pure compound 1-acetyl-2,3-dihydroquinolin-4(1*H*)-one (**6a**) as a light brown solid (4.18 g, 47% overall yield) [3]. **6a**: Mp: 94-95 °C. ^{1}H NMR (400 MHz, DMSO- d_6): δ 7.86 (d, J = 7.6 Hz, 1H), 7.71 (d, J = 8.0 Hz, 1H), 7.61 (dd, J = 8.0 Hz, 1H), 7.29 (dd, J = 7.6 Hz, 1H), 4.13 (t, J = 6.2 Hz, 2H), 2.78 (t, J = 6.2 Hz, 2H), 2.29 (s, 3H). ^{13}C NMR (101 MHz, DMSO-d_6): δ 193.39, 168.55, 143.34, 133.22, 125.97, 124.87, 124.24, 123.97, 43.46, 38.40, 22.60. HRMS (ESI): *m/z* [M + Na]$^+$ calcd for $C_{11}H_{11}NNaO_2$: 212.0687; found 212.0682.

1-Acetyl-6-methoxy-2,3-dihydroquinolin-4(1*H*)-one (6b)

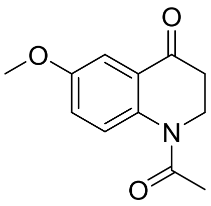

White solid. Yield: 0.59 g, 36%. Mp: 109-110 °C. ^{1}H NMR (400 MHz, $CDCl_3$): δ 7.46 (d, J = 2.7 Hz, 1H), 7.33 (m, 1H), 7.12 (dd, J = 8.8, 2.9 Hz, 1H), 4.27 – 4.14 (m, 2H), 3.86 (s, 3H), 2.79 (t, J = 6.2 Hz, 2H), 2.31 (s, 3H). ^{13}C NMR (101 MHz, $CDCl_3$): δ 194.01, 169.25, 157.13, 137.58, 126.98, 125.63, 121.96, 109.47, 55.73, 43.92, 39.45, 22.97. HRMS (ESI): *m/z* [M + Na]$^+$ calcd for $C_{12}H_{13}NaNO_3$: 242.0788; found 242.0799.

1-Acetyl-6-methyl-2,3-dihydroquinolin-4(1*H*)-one (6c)

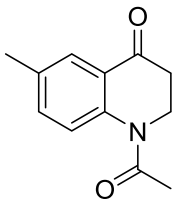

White solid. Yield: 0.57 g, 43%. Mp: 129-130 °C. ^{1}H NMR (400 MHz, $CDCl_3$): δ

7.81 (s, 1H), 7.37 (d, *J* = 6.8 Hz, 1H), 7.31 (m, 1H), 4.23 (t, *J* = 5.9 Hz, 2H), 2.78 (t, *J* = 6.2 Hz, 2H), 2.38 (s, 3H), 2.32 (s, 3H). ^{13}C NMR (101 MHz, $CDCl_3$): *δ* 194.29, 169.32, 141.64, 135.56, 134.94, 127.72, 125.89, 124.00, 43.92, 39.55, 23.06, 20.77. HRMS (ESI): *m/z* $[M + Na]^+$ calcd for $C_{12}H_{13}NaNO_2$: 226.0838; found 226.0844.

1-Acetyl-6-fluoro-2,3-dihydroquinolin-4(1*H*)-one (6d)

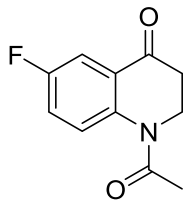

White solid. Yield: 0.55 g, 51%. Mp: 146-147 °C. ^{1}H NMR (400 MHz, $CDCl_3$): *δ* 7.67 (dd, *J* = 8.3, 3.1 Hz, 1H), 7.53 (m, 1H), 7.30 – 7.23 (m, 1H), 4.22 (t, *J* = 5.7 Hz, 2H), 2.81 (t, *J* = 6.2 Hz, 2H), 2.34 (s, 3H). ^{13}C NMR (101 MHz, $CDCl_3$): *δ* 192.83, 169.17, 159.80 (d, J = 247.8 Hz), 140.08, 127.51 (d, *J* = 6.1 Hz), 126.31 (d, *J* = 6.1 Hz), 121.31 (d, *J* = 23.5 Hz), 113.44 (d, *J* = 23.4 Hz), 44.37, 39.21, 23.06. HRMS (ESI): *m/z* $[M + Na]^+$ calcd for $C_{11}H_{10}NaFNO_2$: 230.0588; found 230.0578.

1-Acetyl-6-chloro-2,3-dihydroquinolin-4(1*H*)-one (6e)

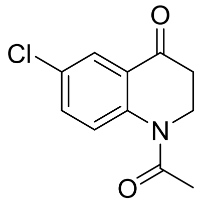

White solid. Yield: 0.62 g, 56%. Mp: 150-151 °C. ^{1}H NMR (400 MHz, $CDCl_3$): *δ* 7.98 (s, 1H), 7.56 – 7.44 (m, 2H), 4.22 (t, *J* = 6.1 Hz, 2H), 2.81 (t, *J* = 6.2 Hz, 2H), 2.35 (s, 3H). ^{13}C NMR (101 MHz, $CDCl_3$): *δ* 192.60, 169.15, 142.26,

133.90, 131.33, 127.33, 126.93, 125.72, 44.25, 39.21, 23.19. HRMS (ESI): *m/z* [M + Na]$^+$ calcd for $C_{11}H_{10}NaClNO_2$: 246.0292; found 246.0305.

1-Acetyl-6-(trifluoromethyl)-2,3-dihydroquinolin-4(1*H*)-one (6f)

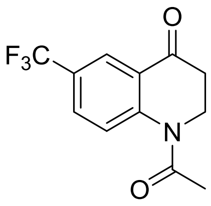

White solid. Yield: 0.69 g, 45%. Mp: 120-121 °C. ^{1}H NMR (400 MHz, $CDCl_3$): δ 8.31 (s, 1H), 7.81 – 7.69 (m, 2H), 4.25 (t, J = 6.3 Hz, 2H), 2.86 (t, J = 6.3 Hz, 2H), 2.40 (s, 3H). ^{13}C NMR (101 MHz, $CDCl_3$): δ 192.35, 169.29, 146.37, 130.42 (q, J = 3.4 Hz), 127.45 (q, J = 33.7 Hz), 125.57, 125.15 (q, J = 3.8 Hz), 124.74, 123.40 (q, J = 271.38 Hz), 44.41, 39.12, 23.38. HRMS (ESI): *m/z* [M + Na]$^+$ calcd for $C_{12}H_{10}NaF_3NO_2$: 280.0556; found 280.0569.

1-Acetyl-5,7-dimethyl-2,3-dihydroquinolin-4(1*H*)-one (6g)

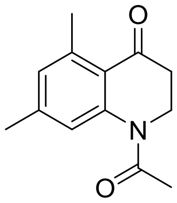

White solid. Yield: 0.57 g, 52%. Mp: 96-97 °C. ^{1}H NMR (400 MHz, $CDCl_3$): δ 6.97 (s, 1H), 6.93 (s, 1H), 4.16 (t, J = 6.3 Hz, 2H), 2.76 (t, J = 6.3 Hz, 2H), 2.61 (s, 3H), 2.37 (s, 3H), 2.27 (s, 3H). ^{13}C NMR (101 MHz, $CDCl_3$): δ 195.68, 169.27, 145.13, 143.64, 142.02, 130.80, 123.30, 123.06, 43.04, 40.94, 22.88, 22.53, 21.71. HRMS (ESI): *m/z* [M + Na]$^+$ calcd for $C_{13}H_{15}NaNO_2$: 240.0995; found 240.0994.

2.2 Representative procedure and characterization data for 1-acetyl-2,3-dihydroquinolin-4(1*H*)-one phenylhydrazones 7

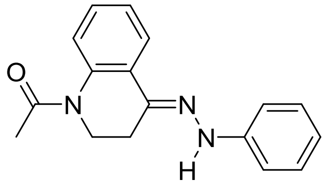

Synthesis of 7a: A catalytic amount of acetic acid was added into a mixture of phenylhydrazine (1.61 g, 14.85 mmol) and 1-acetyl-2,3-dihydroquinolin-4(1*H*)-one **6a** (2.81 g, 14.85 mmol) in anhydrous *n*-propanol (10 mL) at room temperature. The reaction was refluxed under an atmosphere of nitrogen for 5 h. Then the solution was concentrated, and the residual solid was recrystallized from hot *n*-propanol to give 1-acetyl-2,3-dihydroquinolin-4(1*H*)-one phenylhydrazone (**7a**) as a white solid (3.77 g, 13.46 mmol, yield 91%). In all manipulations, special care was taken to minimize the exposure time of the hydrazone to air. Mp: 180-181 °C. ^{1}H NMR (400 MHz, DMSO-d_6): δ 9.34 (s, 1H), 8.05 (d, J = 7.7 Hz, 1H), 7.52 – 7.33 (m, 1H), 7.32 – 7.17 (m, 6H), 6.78 (dd, J = 6.6 Hz, 1H), 3.98 – 3.82 (m, 2H), 2.83 – 2.69 (m, 2H), 2.18 (s, 3H). ^{13}C NMR (101 MHz, DMSO-d_6): δ 169.02, 146.14, 139.31, 137.72, 129.41, 128.89, 127.82, 125.84, 125.11, 124.32, 119.73, 113.38, 27.68, 22.98. HRMS (ESI): *m/z* $[M]^+$ calcd for $C_{17}H_{17}N_3O$: 280.1444; found 280.1454.

1-Acetyl-6-methoxy-2,3-dihydroquinolin-4(1*H*)-one phenylhydrazone (7b)

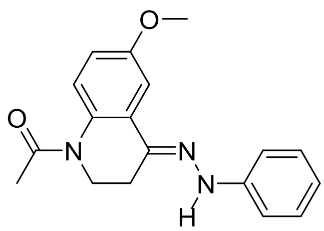

Light brown solid. Yield: 0.40 g, 93%. Mp: 248-249 °C. ^{1}H NMR (400 MHz, DMSO-d_6): δ 9.35 (s, 1H), 7.51 (d, J = 2.8 Hz, 1H), 7.35 – 7.27 (m, 1H), 7.28 – 7.20 (m, 4H), 6.87 (d, J = 7.5 Hz, 1H), 6.79 (dd, J = 6.4 Hz, 1H), 3.89 (s, 2H), 3.81 (s, 3H), 2.72 (s, 2H), 2.10 (s, 3H). ^{13}C NMR (151 MHz, DMSO-d_6): δ 169.00, 157.32, 146.07, 137.64, 133.04, 130.16, 129.44, 126.21, 119.82, 114.40, 113.43, 108.12, 55.73, 40.54, 27.55, 22.72. HRMS (ESI): *m/z* [M + Na]$^+$ calcd for $C_{18}H_{19}NaN_3O_2$: 332.1369; found 332.1379.

1-Acetyl-6-methyl-2,3-dihydroquinolin-4(1*H*)-one phenylhydrazone (7c)

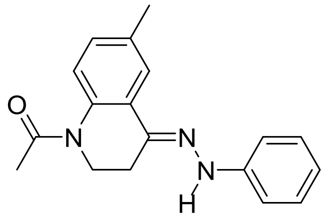

White solid. Yield: 0.36 g, 87%. Mp: 190-191 °C. ^{1}H NMR (600 MHz, DMSO-d_6): δ 9.30 (s, 1H), 7.85 (d, J = 1.1 Hz, 1H), 7.35 – 7.19 (m, 5H), 7.10 (d, J = 7.0 Hz, 1H), 6.83 – 6.75 (m, 1H), 3.97 – 3.79 (m, 2H), 2.85 – 2.66 (m, 2H), 2.36 (s, 3H), 2.15 (s, 3H). ^{13}C NMR (151 MHz, DMSO-d_6): δ 168.95, 146.17, 137.89, 137.08, 135.20, 129.41, 128.71, 124.91, 124.39, 119.71, 113.41, 27.68, 22.90, 21.25. HRMS (ESI): m/z [M + Na]$^+$ calcd for $C_{18}H_{19}NaN_3O$:

316.1420; found 316.1425.

1-Acetyl-6-fluoro-2,3-dihydroquinolin-4(1*H*)-one phenylhydrazone (7d)

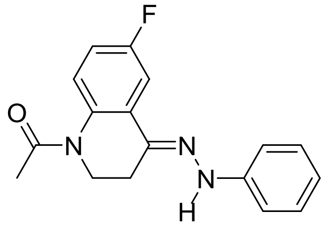

Light brown solid. Yield: 0.35 g, 84%. Mp: 206-207 °C. ^{1}H NMR (600 MHz, DMSO-d_6): δ 9.47 (s, 1H), 7.74 (dd, J = 10.0, 3.0 Hz, 1H), 7.53 – 7.41 (m, 1H), 7.31 – 7.27 (m, 2H), 7.27 – 7.23 (m, 2H), 7.15 – 7.08 (m, 1H), 6.84 – 6.78 (m, 1H), 3.98 – 3.83 (m, 2H), 2.88 – 2.65 (m, 2H), 2.17 (s, 3H). ^{13}C NMR (151 MHz, DMSO-d_6): δ 169.13, 160.09 (d, J = 261.7 Hz), 159.22, 145.85, 136.71, 136.70 (d, J = 2.2 Hz), 135.74, 130.87, 129.45, 127.26, 120.06, 114.79, 113.56, 109.85, 40.55, 27.29, 22.92. HRMS (ESI): *m/z* $[M + Na]^+$ calcd for $C_{17}H_{16}NaFN_3O$: 320.1170; found 320.1161.

1-Acetyl-6-chloro-2,3-dihydroquinolin-4(1*H*)-one phenylhydrazone (7e)

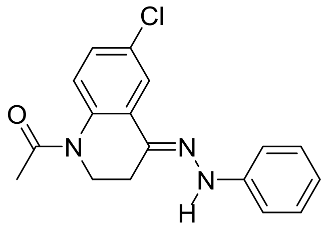

Light brown solid. Yield: 0.39 g, 89%. Mp: 201-202 °C. ^{1}H NMR (400 MHz, DMSO-d_6): δ 9.48 (s, 1H), 7.98 (d, J = 2.2 Hz, 1H), 7.58 – 7.38 (m, 1H), 7.34 – 7.19 (m, 5H), 6.84 – 6.76 (m, 1H), 3.95 – 3.86 (m, 2H), 2.83 – 2.70 (m, 2H), 2.19 (s, 3H). ^{13}C NMR (101 MHz, DMSO-d_6): δ 169.10, 145.81, 137.86, 136.38, 130.59, 130.04, 129.47, 127.25, 127.07, 123.28, 120.08, 113.53, 27.30, 23.02.

HRMS (ESI): *m/z* [M + Na]$^+$ calcd for $C_{17}H_{16}NaClN_3O$: 336.0874; found 336.0886.

1-Acetyl-6-(trifluoromethyl)-2,3-dihydroquinolin-4(1*H*)-one phenylhydrazone (7f)

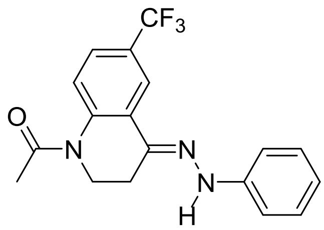

Light brown solid. Yield: 0.45 g, 92%. Mp: 183-184 °C. ^{1}H NMR (400 MHz, DMSO-d_6): δ 9.54 (s, 1H), 8.30 (s, 1H), 7.71 (d, J = 7.5 Hz, 1H), 7.58 (d, J = 8.5 Hz, 1H), 7.31 – 7.20 (m, 4H), 6.87 – 6.77 (m, 1H), 4.00 – 3.91 (m, 2H), 2.90 – 2.78 (m, 2H), 2.25 (s, 3H). ^{13}C NMR (101 MHz, DMSO-d_6): δ 169.31, 145.75, 142.02, 136.39, 129.53, 129.25, 126.14, 125.96 (q, J = 33.7 Hz), 124.65 (q, J = 271.9 Hz), 123.86, 120.70 (q, J = 3.9 Hz), 120.68, 120.17, 113.48, 41.82, 27.30, 23.20. HRMS (ESI): *m/z* [M + Na]$^+$ calcd for $C_{18}H_{16}NaF_3N_3O$: 370.1143; found 370.1127.

1-Acetyl-5,7-dimethyl-2,3-dihydroquinolin-4(1*H*)-one phenylhydrazone (7g)

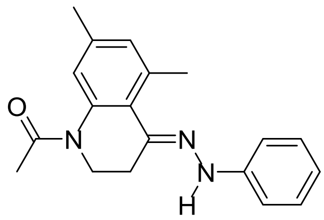

Light brown solid. Yield: 0.38 g, 89%. Mp: 230-231 °C. ^{1}H NMR (600 MHz,

DMSO-d_6): δ 9.16 (s, 1H), 7.25 – 7.21 (m, 2H), 7.20 – 7.17 (m, 2H), 7.09 – 7.03 (m, 1H), 7.02 - 6.94 (m, 1H), 6.79 – 6.75 (m, 1H), 3.92 – 3.75 (m, 2H), 2.90 - 2.73 (m, 2H), 2.64 (s, 3H), 2.30 (s, 3H), 2.15 (s, 3H). ^{13}C NMR (151 MHz, DMSO-d_6): δ 168.67, 146.35, 141.18, 139.77, 137.03, 136.58, 130.85, 129.42, 125.51, 123.67, 119.60, 113.24, 40.54, 29.39, 24.14, 22.69, 21.13. HRMS (ESI): *m/z* [M + Na]$^+$ calcd for $C_{19}H_{21}NaN_3O$: 330.1582; found 330.1585.

2.3 Representative procedure and characterization data for 4-acetoxy-1-acetyl-4-phenylazo-1,2,3,4-tetrahydroquinolines 8

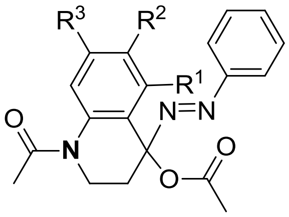

Synthesis of 8a: 1-Acetyl-2,3-dihydroquinolin-4(1*H*)-one phenylhydrazone (**7a**, 2.91 g, 10.42 mmol) was added in portions slowly into a mixture of PhI(OAc)$_2$ (4.03 g,12.5 mmol) in glacial acetic acid (10 mL) at room temperature (exothermic). The reaction mixture turned from colorless to brown during stirring. After the reaction was stirred at room temperature for 30 min, the solution was poured into stirred ice water (20 mL) and sat. aq. $NaHCO_3$ was added dropwise into the stirring solution cooled in an ice bath until free from acetic acid. Then the mixture was extracted with EtOAc (20 mL × 3), the combined organic layers were dried over anhydrous Na_2SO_4, filtered and the filtrate was concentrated under reduced pressure. The crude product was purified by column chromatography on silica gel (EtOAc/petroleum ether

(boiling point range 60–90 °C) 1:2, 0.5% triethylamine in petroleum ether) to give 4-acetoxy-1-acetyl-4-phenylazo-1,2,3,4-tetrahydroquinoline (**8a**) as a yellow oil (3.02 g, 8.92 mmol; yield 86%). ^{1}H NMR (400 MHz, $CDCl_3$): *δ* 7.74 – 7.66 (m, 2H), 7.53 – 7.43 (m, 4H), 7.40 – 7.30 (m, 2H), 7.20 (dd, *J* = 8.0 Hz, 1H), 4.27 – 4.16 (m, 1H), 4.13 – 4.03 (m, 1H), 3.10 – 2.97 (m, 1H), 2.68 – 2.57 (m, 1H), 2.29 (s, 3H), 2.22 (s, 3H). ^{13}C NMR (101 MHz, $CDCl_3$): *δ* 170.09, 169.15, 151.00, 139.56, 131.48, 129.05, 128.92, 128.03, 125.55, 124.64, 122.81, 98.00, 41.63, 33.83, 23.04, 21.85. HRMS (ESI): *m/z* $[M + K]^+$ calcd for $C_{19}H_{19}KN_3O_3$: 376.1058; found 376.1067.

4-Acetoxy-1-acetyl-6-methoxy-4-phenylazo-1,2,3,4-tetrahydroquinoline (8b)

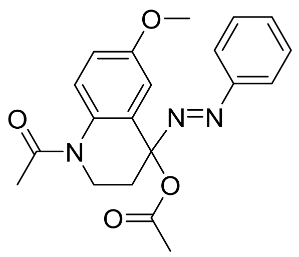

Yellow oil. Yield: 0.22 g, 84%. ^{1}H NMR (600 MHz, CD_3OD): *δ* 7.70 – 7.65 (m, 2H), 7.53 – 7.46 (m, 3H), 7.34 – 7.17 (m, 1H), 7.04 (s, 1H), 6.96 (d, *J* = 7.5 Hz, 1H), 4.13 – 3.99 (m, 2H), 3.78 (s, 3H), 3.05 - 2.86 (m, 1H), 2.67 - 2.50 (m, 1H), 2.38 – 2.00 (m, 6H). ^{13}C NMR (151 MHz, CD_3OD): *δ* 171.16, 169.36, 157.88, 151.08, 132.42, 131.42, 128.96, 125.94, 122.23, 114.21, 112.36, 97.82, 54.66, 40.79, 33.39, 21.49, 20.30. HRMS (ESI) *m/z* $[M + Na]^+$ calcd for $C_{20}H_{21}NaN_3O_4$: 390.1424; found 390.1420.

4-Acetoxy-1-acetyl-6-methyl-4-phenylazo-1,2,3,4-tetrahydroquinoline (8c)

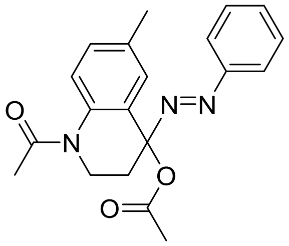

Yellow oil. Yield: 0.19 g, 77%. ^{1}H NMR (600 MHz, CD_3OD): δ 7.69 – 7.63 (m, 2H), 7.49 – 7.43 (m, 3H), 7.33 (s, 1H), 7.29 – 7.22 (m, 1H), 7.16 (d, J = 7.6 Hz, 1H), 4.11 – 3.98 (m, 2H), 3.09 - 3.91 (m, 1H), 2.65 – 2.49 (m, 1H), 2.29 (s, 3H), 2.26 – 2.09 (m, 6H). ^{13}C NMR (151 MHz, CD_3OD): δ 171.04, 169.43, 151.07, 131.36, 129.44, 128.93, 127.71, 124.57, 122.25, 97.91, 33.45, 21.66, 20.38, 19.73. HRMS (ESI): m/z $[M + Na]^+$ calcd for $C_{20}H_{21}NaN_3O_3$: 374.1475; found 374.1482.

4-Acetoxy-1-acetyl-6-fluoro-4-phenylazo-1,2,3,4-tetrahydroquinoline (8d)

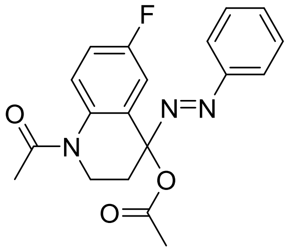

Yellow oil. Yield: 0.17 g, 68%. ^{1}H NMR (600 MHz, CD_3OD): δ 7.70 – 7.62 (m, 2H), 7.49 – 7.44 (m, 3H), 7.41 – 7.31 (m, 1H), 7.28 – 7.19 (m, 1H), 7.14 – 7.06 (m, 1H), 4.09 – 4.00 (m, 2H), 3.11 – 2.86 (m, 1H), 2.66 – 2.49 (m, 1H), 2.21 (s, 3H), 2.16 (s, 3H). ^{13}C NMR (151 MHz, CD_3OD): δ 169.53, 167.77, 158.34 (d, J = 214.8 Hz), 149.48, 134.06, 130.10, 127.49, 125.28 (d, J = 7.9 Hz), 120.82, 114.20 (d, J = 23.2 Hz), 112.34 (d, J = 24.5 Hz), 95.79, 39.33, 31.62, 20.12, 18.76. HRMS (ESI): m/z $[M + Na]^+$ calcd for $C_{19}H_{18}NaFN_3O_3$: 378.1224; found

378.1216.

4-Acetoxy-1-acetyl-6-chloro-4-phenylazo-1,2,3,4-tetrahydroquinoline (8e)

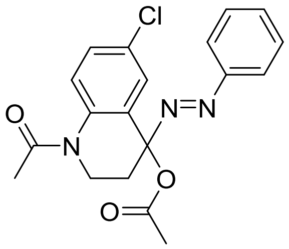

Yellow oil. Yield: 0.21 g, 81%. ^{1}H NMR (400 MHz, $CDCl_3$): *δ* 7.72 (dd, *J* = 6.6, 3.0 Hz, 2H), 7.51 – 7.44 (m, 4H), 7.43 – 7.32 (m, 1H), 7.32 – 7.26 (m, 1H), 4.21 – 4.10 (m, 1H), 4.09 – 4.00 (m, 1H), 3.04 (s, 1H), 2.65 – 2.53 (m, 1H), 2.28 (s, 3H), 2.23 (s, 3H). ^{13}C NMR (101 MHz, $CDCl_3$): *δ* 169.74, 169.04, 150.85, 138.04, 131.75, 130.72, 129.12, 129.06, 127.94, 125.92, 122.90, 97.37, 41.94, 33.47, 23.10, 21.83. HRMS (ESI): *m/z* $[M + Na]^+$ calcd for $C_{19}H_{18}NaClN_3O_3$: 394.0929; found 394.0930.

4-Acetoxy-1-acetyl-6-(trifluoromethyl)-4-phenylazo-1,2,3,4-tetrahydro-quinoline (8f)

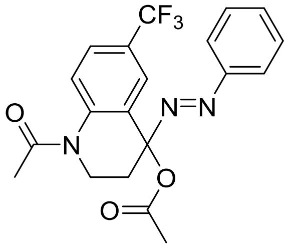

Yellow oil. Yield: 0.20 g, 71%. ^{1}H NMR (600 MHz, CD_3OD): *δ* 7.85 - 7.76 (m, 1H), 7.74 (s, 1H), 7.71 – 7.66 (m, 2H), 7.66 – 7.63 (m, 1H), 7.52 – 7.47 (m, 3H), 4.20 – 4.11 (m, 1H), 4.11 – 4.03 (m, 1H), 3.10 – 3.00 (m, 1H), 2.70 – 2.60 (m,

1H), 2.30 (s, 3H), 2.19 (s, 3H). ^{13}C NMR (151 MHz, CD_3OD): δ 169.56, 167.78, 149.48, 140.71, 130.16, 127.50, 125.22 (q, J = 21.92 Hz), 123.90, 123.28, 123.25, 122.70 (q, J = 253.11 Hz), 120.78, 95.38, 40.64, 31.23, 20.45, 18.69. HRMS (ESI): m/z [M + Na]$^+$ calcd for $C_{20}H_{18}NaF_3N_3O_3$: 428.1192; found 428.1191.

4-Acetoxy-1-acetyl-5,7-dimethyl-4-phenylazo-1,2,3,4-tetrahydroquinoline (8g)

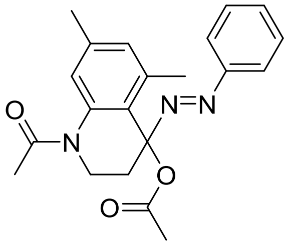

Yellow oil. Yield: 0.20 g, 76%. ^{1}H NMR (400 MHz, CD_3OD): δ 7.65 (dd, J = 6.7, 2.9 Hz, 2H), 7.51 – 7.44 (m, 3H), 7.08 - 6.89 (m, 1H), 6.86 (s, 1H), 4.53 – 4.31 (m, 1H), 3.66 – 3.49 (m, 1H), 2.69 – 2.58 (m, 1H), 2.39 – 2.13 (m, 13H). ^{13}C NMR (101 MHz, CD_3OD): δ 169.25, 151.30, 138.39, 136.98, 131.46, 130.35, 129.02, 123.22, 122.16, 100.36, 40.56, 33.92, 21.58, 20.97, 20.33, 19.62. HRMS (ESI): m/z [M + Na]$^+$ calcd for $C_{21}H_{23}NaN_3O_3$: 388.1632; found 388.1635.

2.4 General procedure and characterization data for 5,6-dihydro-4*H*-benzo[*b*][1,2,4]triazolo[1,5-*d*][1,4]diazepinium salts 10

A solution of 1-acetyl-4-acetoxy-4-phenylazo-1,2,3,4-tetrahydroquinoline (**8**, 0.25 mmol) in dry CH_2Cl_2 (2 mL) was added dropwise slowly to a mixture of nitrile **9** (0.35 mmol) and $AlCl_3$ (0.35 mmol) in CH_2Cl_2 (5 mL) at −40 °C under

an atmosphere of nitrogen (exothermic). After stirring at this temperature for 0.5 h, the reaction was allowed to warm to room temperature and stirred for additional 1 h. Then the mixture was cooled to 0 °C and extracted with H_2O (1 mL × 3). The combined aqueous phase was concentrated under reduced pressure and purified by reverse flash column chromatography. The collected fractions were lyophilized to afford the respective 5,6-dihydro-4*H*-benzo[*b*][1,2,4]triazolo[1,5-*d*][1,4]diazepinium salt **10**.

For the chloride salt **10**, reverse flash column chromatography conditions were as follows: C18, acetonitrile = 5% in H_2O and acetonitrile; 0.5% hydrochloric acid in H_2O. For the trifluoroacetic salt **10**, the reverse flash column chromatography conditions were: C18, acetonitrile = 5% in H_2O and acetonitrile; 0.5% trifluoroacetic acid in H_2O.

The picric salts **10** were obtained by exchanging the Cl^- or CF_3COO^- anion by treatment with picric acid. The following procedure was representative: 0.9–1.1% picric acid solution was added dropwise into the corresponding salt **10** dissolved in H_2O (1 mL) and a brown suspension appeared. The picric acid solution was added dropwise continually until no further suspension appeared. The mixture was filtered, and the filtered cake was washed with H_2O (0.5 mL × 2), collected and dried in vacuum to give the picric salt **10**.

6-Acetyl-2-methyl-1-phenyl-5,6-dihydro-4*H*-benzo[*b*][1,2,4]tri-azolo[1,5-*d*][1,4]diazepinium picrate (10a)

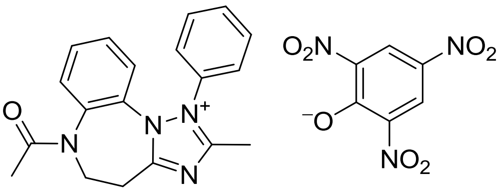

Yellow solid. Yield: 71.9 mg, 81%. Mp: 251-252 °C. ^{1}H NMR (400 MHz, DMSO-*d*$_6$): *δ* 8.59 (s, 2H), 8.23 – 7.95 (m, 1H), 7.85 – 7.54 (m, 5H), 7.43 (t, *J* = 7.8 Hz, 1H), 7.39 – 7.24 (m, 1H), 6.99 (d, *J* = 8.1 Hz, 1H), 5.01 – 4.85 (m, 1H), 3.79 – 3.62 (m, 1H), 3.56 – 3.45 (m, 1H), 3.14 – 2.98 (m, 1H), 2.56 (s, 3H), 1.77 (s, 3H). ^{13}C NMR (101 MHz, DMSO-*d*$_6$): *δ* 169.90, 161.27, 160.59, 159.47, 142.32, 136.22, 133.35, 132.92, 132.34, 131.07, 130.36, 129.98, 128.18, 126.31, 125.68, 124.60, 49.37, 24.05, 23.59, 13.86. HRMS (ESI): *m/z* [M]$^+$ calcd for $C_{19}H_{19}N_4O^+$: 319.1553; found 319.1566.

6-Acetyl-2,9-dimethyl-1-phenyl-5,6-dihydro-4*H*-benzo[*b*][1,2,4]tri-azolo[1,5-*d*][1,4]diazepinium trifluoroacetate (10b)

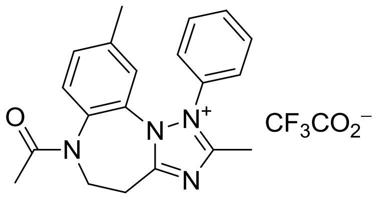

White solid. Yield: 85.9 mg, 77%. Mp: 178-179 °C. ^{1}H NMR (400 MHz, DMSO-*d*$_6$): *δ* 8.23 – 7.96 (m, 1H), 7.90 – 7.53 (m, 4H), 7.46 (d, *J* = 8.2 Hz, 1H), 7.42 – 7.19 (m, 1H), 6.77 (s, 1H), 4.96 – 4.89 (m, 1H), 3.67 (dd, *J* = 12.9, 7.5 Hz, 1H), 3.50 (dd, *J* = 15.3, 5.8 Hz, 1H), 3.14 – 3.00 (m, 1H), 2.56 (s, 3H), 2.12 (s, 3H), 1.76 (s, 3H). ^{13}C NMR (101 MHz, DMSO-*d*$_6$): *δ* 169.99, 160.54, 159.50,

158.57 (q, *J* = 34.6 Hz), 139.91, 133.67, 133.48, 133.37, 131.89, 131.01, 130.38, 127.91, 126.32, 116.62 (q, *J* = 294.3 Hz), 49.25, 24.11, 23.53, 20.83, 13.87, 13.85. HRMS (ESI): *m/z* $[M]^+$ calcd for $C_{20}H_{21}N_4O^+$: 333.1726; found 333.1710.

6-Acetyl-9-methoxy-2-methyl-1-phenyl-5,6-dihydro-4*H*-benzo[*b*][1,2,4]tri-azolo[1,5-*d*][1,4]diazepinium chloride (10c)

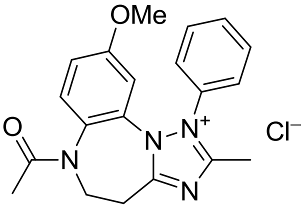

White solid. Yield: 83.7 mg, 87%. Mp: 211-212 °C. ^{1}H NMR (400 MHz, DMSO-d_6): δ 8.31 – 7.94 (m, 1H), 7.89 – 7.58 (m, 4H), 7.53 – 7.29 (m, 1H), 7.22 (dd, *J* = 8.9, 2.6 Hz, 1H), 6.53 (d, *J* = 2.6 Hz, 1H), 4.94 (td, *J* = 12.8, 6.3 Hz, 1H), 3.67 (dd, *J* = 12.7, 7.8 Hz, 4H), 3.08 (td, *J* = 14.0, 7.9 Hz, 1H), 2.57 (s, 3H), 1.75 (s, 3H). ^{13}C NMR (151 MHz, DMSO-d_6): δ 170.19, 160.61, 159.60, 159.19, 133.46, 133.10, 131.13, 130.48, 128.90, 128.59, 118.24, 111.65, 56.38, 49.27, 40.42, 40.28, 40.14, 40.01, 39.87, 39.73, 39.59, 24.19, 23.50, 13.86. HRMS (ESI): *m/z* $[M]^+$ calcd for $C_{20}H_{21}N_4O_2^+$: 349.1667; found 349.1659.

6-Acetyl-2-cyclopropyl-1-phenyl-5,6-dihydro-4*H*-benzo[*b*][1,2,4]tri-azolo[1,5-*d*][1,4]diazepinium chloride (10d)

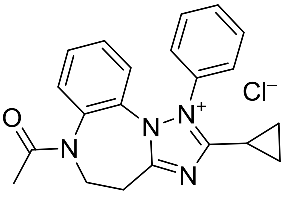

White solid. Yield: 79.0 mg, 83%. Mp: 156-157 °C. ^{1}H NMR (400 MHz,

DMSO-d_6): δ 8.31 – 7.94 (m, 1H), 7.90 – 7.50 (m, 6H), 7.42 (t, J = 7.8 Hz, 1H), 7.01 (d, J = 8.1 Hz, 1H), 4.89 (td, J = 12.9, 6.3 Hz, 1H), 3.68 (dd, J = 12.8, 7.4 Hz, 1H), 3.45 (dd, J = 15.2, 5.7 Hz, 1H), 3.00 (td, J = 13.7, 7.6 Hz, 1H), 1.93 (dd, J = 10.7, 6.4 Hz, 1H), 1.79 (s, 3H), 1.45 – 1.25 (m, 4H). ^{13}C NMR (101 MHz, DMSO-d_6): δ 170.02, 164.78, 159.53, 136.10, 133.27, 132.66, 132.33, 131.11, 130.96, 130.27, 129.82, 129.13, 128.13, 126.33, 49.32, 24.13, 23.75, 12.13, 11.37, 8.31. HRMS (ESI): *m/z* [M]$^+$ calcd for $C_{21}H_{21}N_4O^+$: 345.1710; found 345.1704.

6-Acetyl-2-(2-methoxyethyl)-1-phenyl-5,6-dihydro-4*H*-benzo[*b*][1,2,4]tri-azolo[1,5-*d*][1,4]diazepinium chloride (10e)

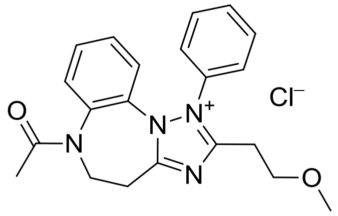

White solid. Yield: 87.8 mg, 88%. Mp: 154-155 °C. ^{1}H NMR (400 MHz, DMSO-d_6): δ 8.16 – 8.05 (m, 1H), 7.89 – 7.78 (m, 2H), 7.74 (dd, J = 7.3 Hz, 1H), 7.64 (dd, J = 7.7 Hz, 1H), 7.60 – 7.49 (m, 1H), 7.46 – 7.32 (m, 2H), 7.06 (d, J = 8.0 Hz, 1H), 4.94 (td, J = 12.6, 6.2 Hz, 1H), 3.87 – 3.81 (m, 2H), 3.71 – 3.68 (m, 1H), 3.55 (dd, J = 15.2, 6.1 Hz, 1H), 3.26 (s, 3H), 3.14 – 2.97 (m, 3H), 1.77 (s, 3H). ^{13}C NMR (101 MHz, DMSO-d_6): δ 169.95, 161.47, 159.78, 136.22, 133.51, 132.99, 132.30, 131.07, 130.04, 129.92, 128.04, 126.58, 67.99, 58.60, 49.44, 27.85, 24.18, 23.65. HRMS (ESI): *m/z* [M]$^+$ calcd for $C_{21}H_{23}N_4O_2^+$: 363.1816; found 363.1812.

6-Acetyl-2-(chloromethyl)-1-phenyl-5,6-dihydro-4*H*-benzo[*b*][1,2,4]tri-azolo[1,5-*d*][1,4]diazepinium chloride (10f)

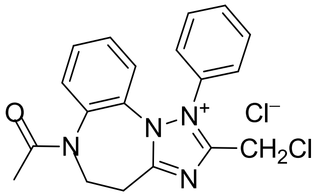

White solid. Yield: 36.0 mg, 37%. Mp: 174-176 °C. ^{1}H NMR (400 MHz, DMSO-d_6): δ 8.25 – 8.03 (m, 1H), 7.91 – 7.80 (m, 2H), 7.76 (t, J = 7.4 Hz, 1H), 7.67 (t, J = 7.7 Hz, 1H), 7.62 – 7.50 (m, 1H), 7.47 – 7.31 (m, 2H), 7.05 (d, J = 8.1 Hz, 1H), 5.12 – 4.87 (m, 3H), 3.72 (dd, J = 12.6, 7.8 Hz, 1H), 3.60 (dd, J = 15.2, 5.9 Hz, 1H), 3.11 (td, J = 14.2, 8.0 Hz, 1H), 1.77 (s, 3H). ^{13}C NMR (101 MHz, DMSO-d_6): δ 169.92, 160.09, 157.93, 136.40, 133.85, 133.34, 132.43, 131.10, 129.97, 129.66, 127.81, 126.66, 49.48, 34.66, 24.21, 23.68. HRMS (ESI): *m/z* [M]$^+$ calcd for $C_{19}H_{18}ClN_4O^+$: 353.1164; found 353.1163.

6-Acetyl-2-benzyl-1-phenyl-5,6-dihydro-4*H*-benzo[*b*][1,2,4]tri-azolo[1,5-*d*][1,4]diazepinium picrate (10g)

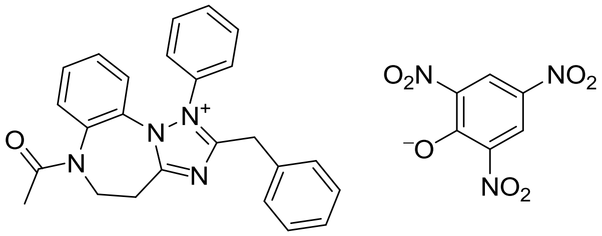

Yellow solid. Yield: 113.8 mg, 73%. Mp: 198-199 °C. ^{1}H NMR (400 MHz, DMSO-d_6) δ 8.59 (s, 2H), 8.09 – 7.97 (m, 1H), 7.86 – 7.71 (m, 3H), 7.64 (dd, J = 7.8 Hz, 1H), 7.61 – 7.52 (m, 1H), 7.47 – 7.32 (m, 7H), 7.09 (d, J = 8.1 Hz, 1H), 4.99 – 4.86 (m, 1H), 4.29 – 4.11 (m, 2H), 3.74 – 3.64 (m, 1H), 3.57 – 3.48 (m, 1H), 3.11 – 2.96 (m, 1H), 1.78 (s, 3H). ^{13}C NMR (101 MHz, DMSO-d_6) δ

169.95, 161.72, 161.27, 159.77, 142.32, 136.26, 133.57, 133.53, 132.98, 132.32, 131.11, 130.12, 129.89, 129.13, 128.06, 128.03, 126.55, 125.68, 124.60, 49.49, 32.91, 24.13, 23.67. HRMS (ESI): *m/z* $[M]^+$ calcd for $C_{25}H_{23}N_4O^+$: 395.1866; found 395.1872.

6-Acetyl-1,2-diphenyl-5,6-dihydro-4*H*-benzo[*b*][1,2,4]triazolo[1,5-*d*][1,4]diazepinium picrate (10h)

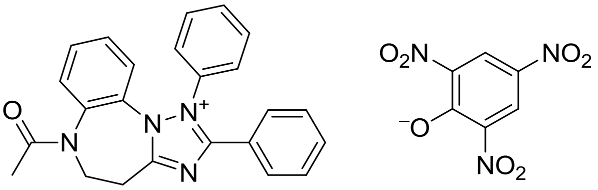

Yellow solid. Yield: 126.5 mg, 83%. Mp: 109-110 °C. ^{1}H NMR (400 MHz, DMSO-d_6): δ 8.59 (s, 2H), 7.98 – 7.87 (m, 1H), 7.85 (d, *J* = 7.9 Hz, 1H), 7.78 – 7.72 (m, 2H), 7.71 – 7.62 (m, 4H), 7.59 (d, *J* = 7.3 Hz, 2H), 7.57 – 7.51 (m, 2H), 7.48 – 7.41 (m, 1H), 7.02 (d, *J* = 8.1 Hz, 1H), 5.00 (td, *J* = 12.8, 6.3 Hz, 1H), 3.75 (dd, *J* = 12.8, 7.6 Hz, 1H), 3.64 (dd, *J* = 15.0, 6.0 Hz, 1H), 3.14 (td, *J* = 14.1, 7.8 Hz, 1H), 1.80 (s, 3H). ^{13}C NMR (101 MHz, DMSO-d_6): δ 170.04, 161.28, 159.65, 158.08, 142.32, 136.52, 133.72, 133.61, 133.18, 132.46, 131.34, 131.26, 130.05, 129.92, 129.67, 127.89, 126.96, 125.69, 124.60, 124.30, 49.47, 24.17, 23.76. HRMS (ESI): *m/z* $[M]^+$ calcd for $C_{24}H_{21}N_4O^+$: 381.1710; found 381.1710.

6-Acetyl-2-(4-methoxyphenyl)-1-phenyl-5,6-dihydro-4*H*-benzo[*b*][1,2,4]triazolo[1,5-*d*][1,4]diazepinium picrate (10i)

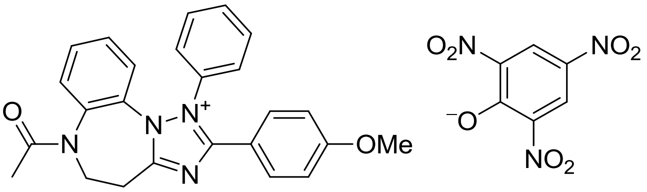

Yellow solid. Yield: 101.7 mg, 91%. Mp: 158-159 °C. ^{1}H NMR (400 MHz, DMSO-d_6): δ 8.59 (s, 2H), 8.07 - 7.86 (m, 1H), 7.82 (d, *J* = 8.0 Hz, 1H), 7.79 – 7.72 (m, 2H), 7.72 – 7.68 (m, 1H), 7.68 – 7.58 (m, 2H), 7.53 (d, *J* = 8.8 Hz, 2H), 7.47 – 7.40 (m, 1H), 7.09 (d, *J* = 8.9 Hz, 2H), 7.02 (d, *J* = 8.1 Hz, 1H), 4.99 (td, *J* = 13.0, 6.4 Hz, 1H), 3.81 (s, 3H), 3.73 (dd, *J* = 12.9, 7.8 Hz, 1H), 3.60 (dd, *J* = 14.9, 6.1 Hz, 1H), 3.10 (td, *J* = 14.4, 7.7 Hz, 1H), 1.78 (s, 3H). ^{13}C NMR (101 MHz, DMSO-d_6): δ 170.03, 163.41, 161.27, 159.41, 157.88, 142.31, 136.50, 133.71, 133.03, 132.40, 131.95, 131.55, 131.42, 129.88, 127.93, 126.95, 125.68, 124.61, 116.17, 115.29, 56.16, 49.38, 24.17, 23.74. HRMS (ESI): *m/z* $[M]^+$ calcd for $C_{25}H_{23}N_4O_2^+$: 411.1816; found 411.1801.

6-Acetyl-2-(4-fluorophenyl)-1-phenyl-5,6-dihydro-4*H*-benzo[*b*][1,2,4]tri-azolo[1,5-*d*][1,4]diazepinium picrate (10j)

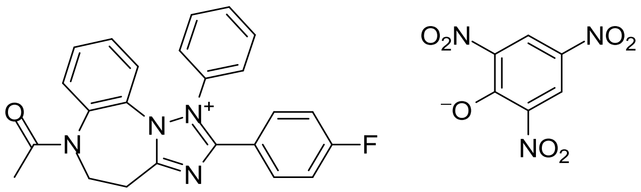

Yellow solid. Yield: 127.1 mg, 82%. Mp: 112-113 °C. ^{1}H NMR (400 MHz, DMSO-d_6): δ 8.59 (s, 2H), 8.03 - 7.87 (m, 1H), 7.85 (d, *J* = 7.8 Hz, 1H), 7.77 – 7.58 (m, 7H), 7.48 – 7.38 (m, 3H), 7.01 (d, *J* = 8.0 Hz, 1H), 5.09 – 4.88 (m, 1H), 3.75 (dd, *J* = 12.2, 8.0 Hz, 1H), 3.63 (dd, *J* = 15.1, 5.6 Hz, 1H), 3.15 (dd, *J* = 21.5, 13.8 Hz, 1H), 1.80 (s, 3H). ^{13}C NMR (101 MHz, DMSO-d_6): δ 170.01, 165.03 (d, *J* = 253.3 Hz), 161.27, 159.65, 157.30, 142.31, 136.51, 133.80, 133.23, 132.94 (d, *J* = 9.6 Hz), 132.47, 131.42, 131.08, 129.97, 127.86, 126.92, 125.68, 124.60, 120.90 (d, *J* = 3.0 Hz), 117.16 (d, *J* = 22.5 Hz), 49.45, 24.15,

23.74. HRMS (ESI): *m/z* $[M]^+$ calcd for $C_{24}H_{20}FN_4O^+$: 399.1616; found 399.1614.

6-Acetyl-2-(4-chlorophenyl)-1-phenyl-5,6-dihydro-4*H*-benzo[*b*][1,2,4]triazolo[1,5-*d*][1,4]diazepinium chloride (10k)

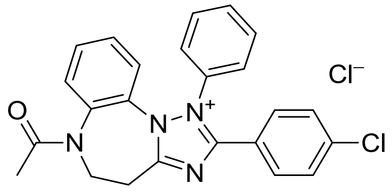

White solid. Yield: 100.4 mg, 89%. Mp: 123-124 °C. ^{1}H NMR (400 MHz, DMSO-d_6): δ 7.87 – 7.84 (m, 1H), 7.81 – 7.72 (m, 2H), 7.68 (dd, *J* = 13.7, 8.1 Hz, 5H), 7.61 – 7.50 (m, 3H), 7.46 (dd, *J* = 7.8 Hz, 1H), 7.04 (d, *J* = 8.2 Hz, 1H), 5.00 (td, *J* = 12.8, 6.3 Hz, 1H), 3.75 (dd, *J* = 12.7, 7.6 Hz, 1H), 3.64 (dd, *J* = 15.1, 6.0 Hz, 1H), 3.21 – 3.08 (m, 1H), 1.80 (s, 3H). ^{13}C NMR (101 MHz, DMSO-d_6): δ 170.01, 159.68, 157.25, 138.63, 136.50, 133.81, 133.27, 132.47, 131.85, 131.40, 131.01, 129.97, 128.75, 127.85, 126.98, 123.22, 49.46, 24.17, 23.76. HRMS (ESI): *m/z* $[M]^+$ calcd for $C_{24}H_{20}ClN_4O^+$: 415.1325; found 415.1339.

6-Acetyl-2-(4-chlorophenyl)-9-fluoro-1-phenyl-5,6-dihydro-4*H*-benzo[*b*][1,2,4]triazolo[1,5-*d*][1,4]diazepinium chloride (10l)

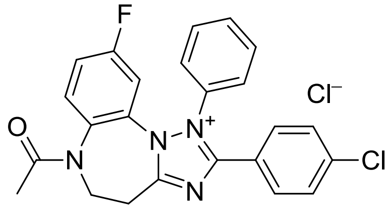

White solid. Yield: 122.5 mg, 74%. Mp: 209-210 °C. ^{1}H NMR (400 MHz, DMSO-d_6): δ 8.10 – 7.82 (m, 2H), 7.81 – 7.57 (m, 9H), 6.87 (dd, *J* = 8.5, 2.9 Hz,

1H), 4.99 (td, *J* = 12.9, 6.3 Hz, 1H), 3.73 (dd, *J* = 12.8, 7.7 Hz, 1H), 3.64 (dd, *J* = 15.1, 6.0 Hz, 1H), 3.23 (td, *J* = 14.9, 7.9 Hz, 1H), 1.80 (s, 3H). ^{13}C NMR (101 MHz, DMSO-d_6): *δ* 170.02, 160.75 (d, *J* = 249.2 Hz), 160.13, 157.48, 138.83, 134.52 (d, *J* = 9.5 Hz), 134.03, 133.26 (d, *J* = 3.5 Hz), 131.86, 131.55, 130.80, 130.06, 128.75 (d, *J* = 11.5 Hz), 123.00, 120.57 (d, *J* = 22.2 Hz), 114.35 (d, *J* = 27.9 Hz), 49.46, 24.07, 23.75. HRMS (ESI): *m/z* [M]$^+$ calcd for $C_{24}H_{19}ClFN_4O^+$: 433.1226; found 433.1221.

6-Acetyl-9-chloro-2-(4-chlorophenyl)-1-phenyl-5,6-dihydro-4*H*-benzo[*b*][1,2,4]triazolo[1,5-*d*][1,4]diazepinium chloride (10m).

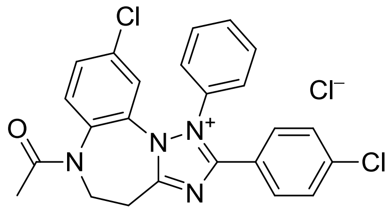

White solid. Yield: 98.4 mg, 81%. Mp: 258-259 °C. ^{1}H NMR (400 MHz, DMSO-d_6): *δ* 7.93 (d, *J* = 8.7 Hz, 1H), 7.86 – 7.78 (m, 3H), 7.77 – 7.69 (m, 3H), 7.67 (d, *J* = 8.7 Hz, 2H), 7.59 (d, *J* = 8.7 Hz, 2H), 7.01 (s, 1H), 4.97 (td, *J* = 13.1, 6.4 Hz, 1H), 3.73 (dd, *J* = 12.6, 7.8 Hz, 1H), 3.64 (dd, *J* = 14.9, 6.1 Hz, 1H), 3.27 (td, 1H), 1.81 (s, 3H). ^{13}C NMR (101 MHz, DMSO-d_6): *δ* 169.92, 160.09, 157.40, 138.85, 135.66, 134.10, 134.03, 133.49, 133.23, 131.85, 131.54, 130.81, 130.08, 128.63, 126.76, 122.98, 49.48, 23.94, 23.81. HRMS (ESI): *m/z* [M]$^+$ calcd for $C_{24}H_{19}Cl_2N_4O^+$: 449.0930; found 449.0948.

6-Acetyl-2-(4-chlorophenyl)-1-phenyl-9-(trifluoromethyl)-5,6-dihydro-4*H*-benzo[*b*][1,2,4]triazolo[1,5-*d*][1,4]diazepinium chloride (10n)

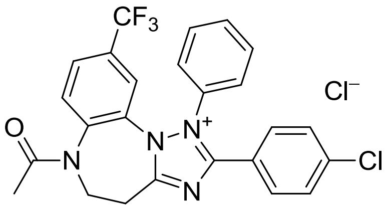

White solid. Yield: 81.8 mg, 63%. Mp: 246-247 °C. ^{1}H NMR (400 MHz, DMSO-d_6): δ 8.13 (dd, J = 19.3, 8.4 Hz, 1H), 8.01 – 7.82 (m, 1H), 7.79 – 7.56 (m, 8H), 7.27 (s, 1H), 5.05 – 4.93 (m, 1H), 3.78 (dd, J = 12.7, 7.6 Hz, 1H), 3.67 (dd, J = 15.0, 5.6 Hz, 1H), 3.38 – 3.25 (m, 1H), 1.84 (s, 3H). ^{13}C NMR (101 MHz, DMSO-d_6): δ 169.82, 160.14, 157.44, 140.45, 138.89, 133.99, 133.93, 131.86, 131.49, 130.76, 130.12, 129.92, 129.79 – 128.87 (q, J = 266.5 Hz), 128.20, 127.12 – 118.95 (q, J = 33.5 Hz), 124.38, 122.95, 49.65, 23.96, 23.81. HRMS (ESI): *m/z* [M]$^+$ calcd for $C_{25}H_{19}ClF_3N_4O^+$: 483.1199; found 483.1203.

6-Acetyl-2-(4-chlorophenyl)-8,10-dimethyl-1-phenyl-5,6-dihydro-4*H*-benzo[*b*][1,2,4]triazolo[1,5-*d*][1,4]diazepinium chloride (10o)

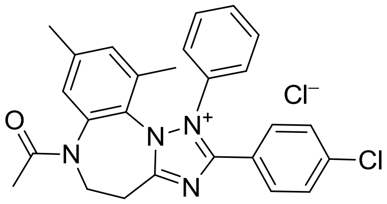

White solid. Yield: 99.5 mg, 83%. Mp: 203-204 °C. ^{1}H NMR (400 MHz, DMSO-d_6): δ 7.71 (dd, J = 7.5 Hz, 1H), 7.66 – 7.59 (m, 4H), 7.57 – 7.36 (m, 5H), 7.19 (s, 1H), 4.82 (td, J = 12.8, 6.3 Hz, 1H), 3.66 – 3.51 (m, 2H), 3.16 (td, J = 14.0, 7.8 Hz, 1H), 2.36 (s, 3H), 2.01 (s, 3H), 1.78 (s, 3H). ^{13}C NMR (101 MHz, DMSO-d_6): δ 170.38, 161.44, 157.92, 144.47, 138.38, 137.48, 137.34,

133.42, 133.07, 132.52, 131.07, 130.78, 130.14, 129.56, 128.72, 123.99, 123.64, 49.07, 24.09, 23.96, 21.14, 18.93. HRMS (ESI): *m/z* $[M]^+$ calcd for $C_{26}H_{24}ClN_4O^+$: 443.1638; found 443.1644.

6-Acetyl-2-(4-bromophenyl)-1-phenyl-5,6-dihydro-4*H*-benzo[*b*][1,2,4]tri-azolo[1,5-*d*][1,4]diazepinium picrate (10p)

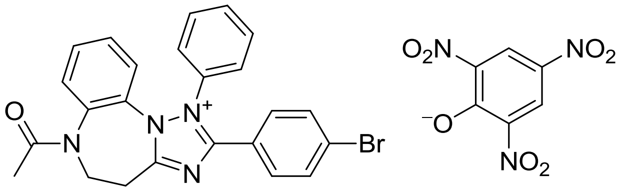

Yellow solid. Yield: 148.0 mg, 86%. Mp: 126-127 °C. ^{1}H NMR (400 MHz, DMSO-d_6): δ 8.59 (s, 2H), 7.99 - 7.88 (m, 1H), 7.85 (d, *J* = 8.0 Hz, 1H), 7.79 (d, *J* = 8.6 Hz, 2H), 7.76 – 7.72 (m, 2H), 7.71 – 7.66 (m, 2H), 7.64 - 7.51 (m, 1H), 7.51 – 7.42 (m, 3H), 7.00 (d, *J* = 8.0 Hz, 1H), 5.00 (td, *J* = 13.0, 6.7 Hz, 1H), 3.75 (dd, *J* = 12.8, 7.4 Hz, 1H), 3.64 (dd, *J* = 15.1, 6.0 Hz, 1H), 3.15 (td, *J* = 14.5, 7.9 Hz, 1H), 1.80 (s, 3H). ^{13}C NMR (101 MHz, DMSO-d_6): δ 170.01, 161.27, 159.74, 157.38, 142.32, 136.50, 133.81, 133.27, 132.91, 132.49, 131.88, 131.42, 130.98, 129.98, 127.84, 127.79, 126.91, 125.67, 124.59, 123.52, 49.47, 24.15, 23.75. HRMS (ESI): *m/z* $[M]^+$ calcd for $C_{24}H_{20}BrN_4O^+$: 459.0815, 461.0796; found 459.0829, 461.0810.

6-Acetyl-2-(4-iodophenyl)-1-phenyl-5,6-dihydro-4*H*-benzo[*b*][1,2,4]tri-azolo[1,5-*d*][1,4]diazepinium chloride (10q)

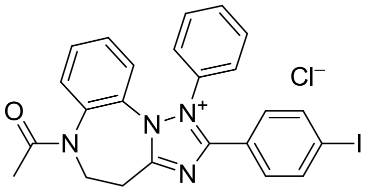

White solid. Yield: 150.8 mg, 82%. Mp: 266-267 °C. ^{1}H NMR (400 MHz, DMSO-d_6): δ 7.95 (d, J = 8.4 Hz, 2H), 7.92 - 7.87 (m, 1H), 7.85 (d, J = 7.9 Hz, 1H), 7.79 – 7.71 (m, 2H), 7.70 – 7.65 (m, 2H), 7.64 - 7.50 (m, 1H), 7.48 – 7.41 (m, 1H), 7.32 (d, J = 8.4 Hz, 2H), 7.02 (d, J = 8.1 Hz, 1H), 4.99 (td, J = 12.9, 6.3 Hz, 1H), 3.75 (dd, J = 12.7, 7.7 Hz, 1H), 3.63 (dd, J = 15.1, 6.1 Hz, 1H), 3.14 (td, J = 14.5, 7.8 Hz, 1H), 1.80 (s, 3H). ^{13}C NMR (101 MHz, DMSO-d_6): δ 170.02, 159.64, 157.67, 138.63, 136.47, 133.77, 133.24, 132.43, 131.53, 131.35, 131.09, 129.93, 127.87, 127.06, 123.77, 102.60, 49.47, 24.19, 23.79. HRMS (ESI): *m/z* [M]$^+$ calcd for $C_{24}H_{20}IN_4O^+$: 507.0676; found 507.0685.

6-Acetyl-1-phenyl-2-(4-(trifluoromethyl)phenyl)-5,6-dihydro-4*H*-benzo[*b*][1,2,4]triazolo[1,5-*d*][1,4]diazepinium chloride (10r)

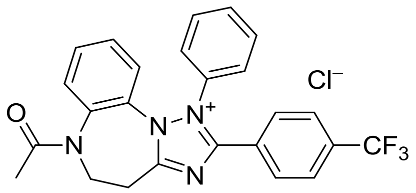

White solid. Yield: 95.8 mg, 79%. Mp: 259-260 °C. ^{1}H NMR (400 MHz, DMSO-d_6): δ 8.05 – 7.84 (m, 4H), 7.81 – 7.58 (m, 7H), 7.47 (t, J = 7.9 Hz, 1H), 7.04 (d, J = 8.1 Hz, 1H), 5.01 (td, J = 12.9, 6.3 Hz, 1H), 3.77 (dd, J = 12.7, 7.7 Hz, 1H), 3.67 (dd, J = 15.1, 6.1 Hz, 1H), 3.18 (td, J = 14.7, 7.9 Hz, 1H), 1.82 (s, 3H). ^{13}C NMR (101 MHz, DMSO-d_6): δ 170.02, 159.83, 156.95, 136.52, 133.87, 133.38, 132.85 (q, J = 32.4 Hz), 132.49, 131.39, 131.15, 130.83, 130.00, 128.35, 127.96, 127.03, 126.65 (d, J = 3.7 Hz), 123.90 (q, J = 272.9 Hz), 149.51, 24.19, 23.78. ^{19}F NMR (376 MHz, DMSO-d_6): δ –61.79. HRMS (ESI): *m/z* [M]$^+$ calcd for $C_{25}H_{20}F_3N_4O^+$: 449.1584; found 449.1584.

6-Acetyl-1-phenyl-2-(*p*-tolyl)-5,6-dihydro-4*H*-benzo[*b*][1,2,4]tri-azolo[1,5-*d*][1,4]diazepinium picrate (10t)

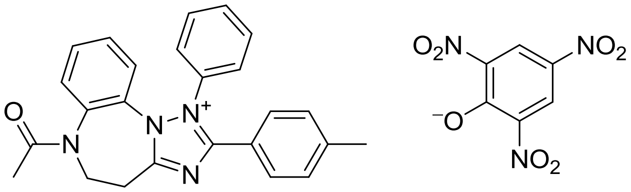

Yellow solid. Yield: 129.4 mg, 83%. Mp: 203-204 °C. ^{1}H NMR (400 MHz, DMSO-d_6): δ 8.59 (s, 2H), 8.04 - 7.86 (m, 1H), 7.84 (d, J = 7.9 Hz, 1H), 7.77 – 7.55 (m, 5H), 7.50 – 7.39 (m, 3H), 7.34 (d, J = 8.1 Hz, 2H), 7.02 (d, J = 8.1 Hz, 1H), 5.00 (td, J = 6.3 Hz, 1H), 3.74 (dd, J = 12.6, 7.7 Hz, 1H), 3.62 (dd, J = 15.0, 6.0 Hz, 1H), 3.12 (td, J = 7.8 Hz, 1H), 2.35 (s, 3H), 1.79 (s, 3H). ^{13}C NMR (101 MHz, DMSO-d_6): δ 170.05, 161.29, 159.58, 158.17, 144.25, 142.30, 136.50, 133.66, 133.10, 132.42, 131.38, 131.33, 130.25, 129.96, 129.89, 127.92, 126.97, 125.70, 124.61, 121.45, 49.45, 24.19, 23.75, 21.56. HRMS (ESI): *m/z* $[M]^+$ calcd for $C_{25}H_{23}N_4O^+$: 395.1866; found 395.1864.

6-Acetyl-1-phenyl-2-(*m*-tolyl)-5,6-dihydro-4*H*-benzo[*b*][1,2,4]tri-azolo[1,5-*d*][1,4]diazepinium chloride (10u)

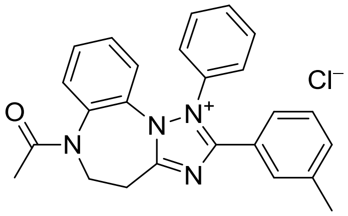

White solid. Yield: 92.6 mg, 86%. Mp: 209-210 °C. ^{1}H NMR (400 MHz, DMSO-d_6): δ 8.06 – 7.88 (m, 1H), 7.85 (dd, J = 8.0, 1.1 Hz, 1H), 7.80 – 7.54 (m, 5H), 7.51 – 7.44 (m, 3H), 7.42 – 7.38 (m, 1H), 7.29 (d, J = 7.9 Hz, 1H), 7.06 (dd, J = 8.2, 1.2 Hz, 1H), 5.06 – 4.92 (m, 1H), 3.85 – 3.75 (m, 1H), 3.70 – 3.58 (m,

1H), 3.24 – 3.07 (m, 1H), 2.58 (s, 3H), 1.87 (s, 3H). ^{13}C NMR (101 MHz, DMSO-d_6): δ 170.21, 159.36, 158.64, 139.68, 136.23, 133.08, 133.03, 132.55, 132.38, 131.75, 131.34, 130.90, 130.82, 129.72, 128.98, 128.15, 127.15, 125.99, 123.53, 49.66, 24.32, 23.92, 20.23. HRMS (ESI): *m/z* [M]$^+$ calcd for $C_{25}H_{23}N_4O^+$: 395.1866; found 395.1860.

6-Acetyl-1-phenyl-2-(*o*-tolyl)-5,6-dihydro-4*H*-benzo[*b*][1,2,4]triazolo[1,5-*d*][1,4]diazepinium chloride (10v)

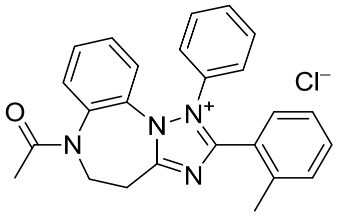

White solid. Yield: 77.6 mg, 72%. Mp: 234-236 °C. ^{1}H NMR (600 MHz, DMSO-d_6): δ 7.85 (dd, *J* = 8.0, 1.1 Hz, 1H), 7.78 – 7.73 (m, 1H), 7.72 – 7.55 (m, 1H), 7.51 – 7.44 (m, 3H), 7.42 – 7.38 (m, 1H), 7.29 (d, *J* = 7.9 Hz, 1H), 7.06 (dd, *J* = 8.2, 1.2 Hz, 1H), 5.01 (td, *J* = 12.9, 6.3 Hz, 1H), 3.76 (dd, *J* = 12.7, 7.7 Hz, 1H), 3.63 (dd, *J* = 15.1, 6.0 Hz, 1H), 3.15 (dd, *J* = 13.2, 7.8 Hz, 1H), 2.31 (s, 3H), 1.81 (s, 3H). ^{13}C NMR (151 MHz, DMSO-d_6): δ 170.04, 159.59, 158.18, 139.19, 136.52, 134.21, 133.68, 133.18, 132.44, 131.34, 131.26, 130.59, 129.91, 129.50, 127.91, 127.08, 127.06, 124.30, 49.46, 24.21, 23.76, 21.29. HRMS (ESI): *m/z* [M]$^+$ calcd for $C_{25}H_{23}N_4O^+$: 395.1866; found 395.1870.

6-Acetyl-2-(2-fluoro-4-methylphenyl)-1-phenyl-5,6-dihydro-4*H*-benzo[*b*][1,2,4]triazolo[1,5-*d*][1,4]diazepinium picrate (10w)

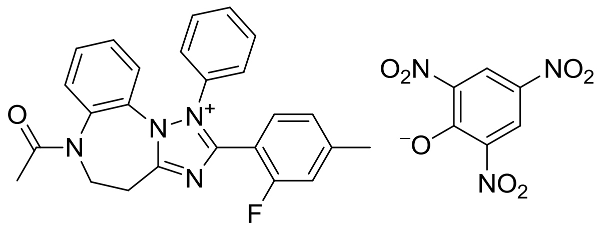

Yellow solid. Yield: 121.9 mg, 76%. Mp: 143-144 °C. ^{1}H NMR (400 MHz, DMSO-d_6): δ 8.59 (s, 2H), 7.86 (d, J = 7.9 Hz, 1H), 7.79 – 7.49 (m, 8H), 7.47 – 7.42 (m, 1H), 7.37 – 7.31 (m, 1H), 7.26 (d, J = 5.8 Hz, 1H), 7.00 (d, J = 8.1 Hz, 1H), 4.98 (td, J = 12.9, 6.0 Hz, 1H), 3.77 (dd, J = 12.7, 7.5 Hz, 1H), 3.65 (dd, J = 15.2, 6.1 Hz, 1H), 3.17 (td, J = 14.0, 7.5 Hz, 1H), 2.21 (s, 3H), 1.84 (s, 3H). ^{13}C NMR (101 MHz, DMSO-d_6): δ 170.14, 161.27, 159.97, 158.21 (d, J = 253.5 Hz), 155.06 (d, J = 2.2 Hz), 142.32, 136.48, 136.39, 136.35, 134.77 (d, J = 3.5 Hz), 133.38, 133.22, 132.54 (d, J = 10.1 Hz), 130.94, 130.87, 129.85, 128.81 (br), 127.98, 127.00, 125.67, 124.59, 117.21 (d, J = 20.6 Hz), 112.02 (d, J = 12.8 Hz), 49.64, 24.22, 23.86, 20.33. HRMS (ESI): *m/z* [M]$^+$ calcd for $C_{25}H_{22}FN_4O^+$: 413.1772; found 413.1764.

6-Acetyl-2-(4-chloro-2-methylphenyl)-1-phenyl-5,6-dihydro-4*H*-benzo[*b*][1,2,4]triazolo[1,5-*d*][1,4]diazepinium picrate (10x)

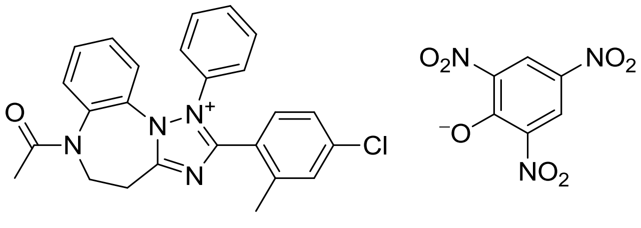

Yellow solid. Yield: 100.2 mg, 67%. Mp: 223-224 °C. ^{1}H NMR (600 MHz,

DMSO-d_6): δ 8.58 (s, 2H), 7.87 (dd, J = 8.0, 1.1 Hz, 1H), 7.71 – 7.67 (m, 1H), 7.66 – 7.62 (m, 2H), 7.61 – 7.56 (m, 2H), 7.55 – 7.49 (m, 2H), 7.48 – 7.44 (m, 1H), 7.29 (dd, J = 8.4, 1.9 Hz, 1H), 7.15 (d, J = 8.4 Hz, 1H), 6.95 (d, J = 8.2 Hz, 1H), 4.99 (td, J = 12.9, 6.2 Hz, 1H), 3.78 (dd, J = 12.7, 7.6 Hz, 1H), 3.65 (dd, J = 15.2, 5.9 Hz, 1H), 3.21 – 3.11 (m, 1H), 2.56 (s, 3H), 1.86 (s, 3H). ^{13}C NMR (151 MHz, DMSO-d_6): δ 170.16, 161.29, 159.57, 157.73, 142.34, 142.30, 137.43, 136.26, 133.23, 133.14, 133.05, 132.49, 131.62, 131.08, 130.58, 129.83, 128.83 (br), 128.11, 126.90, 126.32, 125.67, 124.61, 122.41, 49.66, 24.29, 23.86, 19.92. HRMS (ESI): *m/z* [M]$^+$ calcd for $C_{25}H_{22}ClN_4O^+$: 429.1477; found 429.1476.

2.5 Characterization data for 1-acetyl-2,3-dihydroquinolin-4(1*H*)-one ethoxycarbonylhydrazone 11

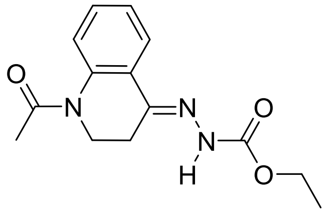

Compound **11** was prepared according to the same as described procedure for the synthesis of **7**. Yield: 83%. White solid. Mp: 139-140 °C. ^{1}H NMR (400 MHz, DMSO-d_6): δ 10.15 (s, 1H), 7.94 (d, J = 8.0 Hz, 1H), 7.51 – 7.40 (m, 1H), 7.35 (t, J = 7.2 Hz, 1H), 7.23 (t, J = 7.6 Hz, 1H), 4.18 (q, J = 7.1 Hz, 2H), 3.85 (t, J = 6.1 Hz, 2H), 2.80 – 2.67 (m, 2H), 2.17 (s, 3H), 1.26 (t, J = 7.1 Hz, 3H). ^{13}C NMR (101 MHz, DMSO-d_6): δ 167.94, 153.48, 144.60, 139.42, 128.37, 126.51, 124.65, 124.02, 60.07, 39.55, 39.34, 39.14, 38.93, 38.72, 38.51, 38.30, 27.15,

21.95, 13.97. HRMS (ESI): *m/z* $[M + H]^+$ calcd for $C_{14}H_{18}N_3O_3$: 276.1343; found 276.1344.

2.6 Procedure and characterization data for 5,6-dihydro-4*H*-benzo[*b*][1,2,4]triazolo[1,5-*d*][1,4]diazepine 13

1-Acetyl-2,3-dihydroquinolin-4(1*H*)-one ethoxycarbonylhydrazone (**11**, 330 mg, 1.20 mmol) was added in portions into a solution of lead tetra-acetate (639 mg, 1.44 mmol) in CH_2Cl_2 (10 mL) at 0 °C. After stirring the reaction mixture at room temperature for 30 min, the reaction was quenched by sat. aq. $NaHCO_3$ solution at 0 °C. The precipitate was removed by filtration through a pad of silica gel, the organic layer was separated, dried over Na_2SO_4, and concentrated under reduced pressure. The residual oil was dissolved in CH_2Cl_2 (2 mL) and slowly added dropwise to a mixture of the appropriate nitrile **9** (1.68 mmol) and $AlCl_3$ (224.0 mg, 1.68 mmol) in CH_2Cl_2 (5 mL) at −40 °C under an atmosphere of nitrogen (exothermic). After stirring at this temperature for 0.5 h, the reaction was allowed to warm to room temperature and stirred at this temperature for another 1 h. Then the reaction was cooled to 0 °C and ice water (1 mL) was added and stirred at 0 °C for 5 min. The organic layer was separated, washed with H_2O (1 mL × 2) and dried with Na_2SO_4. After removal of the solvent under reduced pressure, the crude product was purified via reversed flash column chromatography (C18, acetonitrile = 50–80% in H_2O and acetonitrile; 0.5% NH_4OH in H_2O). The collected fractions were dried via lyophilizer to give the pure product.

6-Acetyl-2-methyl-5,6-dihydro-4*H*-benzo[*b*][1,2,4]triazolo[1,5-*d*][1,4]diazepine (13a)

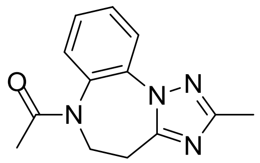

White solid. Yield: 66.9 mg, 23%. Mp: 102-103 °C. ^{1}H NMR (400 MHz, DMSO-d_6): δ 7.95 – 7.91 (m, 1H), 7.62 – 7.53 (m, 2H), 7.49 – 7.43 (m, 1H), 4.76 – 4.65 (m, 1H), 3.33 – 3.23 (m, 2H), 3.11 – 3.00 (m, 1H), 2.31 (s, 3H), 1.75 (s, 3H). ^{13}C NMR (101 MHz, DMSO-d_6): δ 169.12, 160.37, 154.74, 134.45, 134.19, 129.78, 129.52, 128.42, 123.73, 45.34, 26.24, 22.72, 13.98. HRMS (ESI): *m/z* [M + H]$^+$ calcd for $C_{13}H_{14}N_4O$: 243.1243; found 243.1240.

6-Acetyl-2-(2-methoxyethyl)-5,6-dihydro-4*H*-benzo[*b*][1,2,4]triazolo[1,5-*d*][1,4]diazepine (13b)

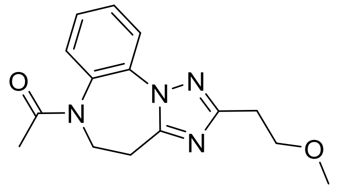

A white solid. Yield: 72.2 mg, 21%. Mp: 110-111 °C. ^{1}H NMR (400 MHz, DMSO-d_6) δ 7.97 – 7.93 (m, 1H), 7.63 – 7.58 (m, 1H), 7.58 – 7.53 (m, 1H), 7.49 – 7.43 (m, 1H), 4.75 – 4.65 (m, 1H), 3.71 (t, *J* = 6.8 Hz, 2H), 3.38 – 3.27 (m, 2H), 3.25 (s, 3H), 3.15 – 3.05 (m, 1H), 2.89 (t, *J* = 6.8 Hz, 2H), 1.77 (s, 3H). ^{13}C NMR (101 MHz, DMSO-d_6) δ 169.09, 161.61, 154.77, 134.34, 134.17, 129.73, 129.47, 128.43, 123.74, 70.22, 58.29, 45.14, 28.71, 26.49, 22.76. HRMS (ESI): *m/z* [M + H]$^+$ calcd for $C_{15}H_{18}N_4O_2$: 287.1492, found 287.1503.

6-Acetyl-2-phenyl-5,6-dihydro-4*H*-benzo[*b*][1,2,4]triazolo[1,5-*d*][1,4]di-azepine (13c)

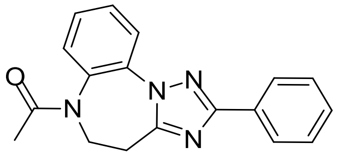

White solid. Yield: 87.7 mg, 24%. Mp: 175-176 °C. ^{1}H NMR (400 MHz, DMSO-d_6): δ 8.11 – 8.06 (m, 3H), 7.68 – 7.59 (m, 2H), 7.54 – 7.46 (m, 4H), 4.83 – 4.72 (m, 1H), 3.51 – 3.34 (m, 2H), 3.23 – 3.14 (m, 1H), 1.78 (s, 3H). ^{13}C NMR (101 MHz, DMSO-d_6): δ 169.23, 161.05, 155.76, 134.48, 134.42, 130.64, 130.14, 129.89, 129.63, 129.33, 128.85, 126.49, 124.00, 45.36, 26.47, 22.78. HRMS (ESI): *m/z* [M + H]$^+$ calcd for $C_{18}H_{16}N_4O$: 305.1397; found 305.1397.

6-Acetyl-2-(*p*-tolyl)-5,6-dihydro-4*H*-benzo[*b*][1,2,4]triazolo[1,5-*d*][1,4]di-azepine (13d)

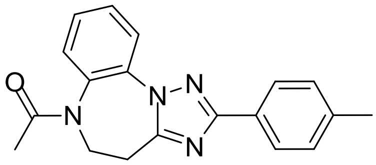

White solid. Yield: 103.2 mg, 27%. Mp: 142-143 °C. ^{1}H NMR (400 MHz, DMSO-d_6): δ 8.10 – 8.05 (m, 1H), 7.97 (d, J = 8.1 Hz, 2H), 7.67 – 7.58 (m, 2H), 7.55 – 7.47 (m, 1H), 7.31 (d, J = 8.0 Hz, 2H), 4.82 – 4.71 (m, 1H), 3.50 – 3.34 (m, 2H), 3.22 – 3.12 (m, 1H), 2.37 (s, 3H), 1.78 (s, 3H). ^{13}C NMR (101 MHz, DMSO-d_6): δ 169.21, 161.10, 155.60, 139.69, 134.49, 134.38, 129.89, 129.85, 129.59, 128.73, 127.92, 126.46, 123.96, 45.31, 26.49, 22.78, 21.45. HRMS (ESI): *m/z* [M + H]$^+$ calcd for $C_{19}H_{18}N_4O$: 319.1542; found 319.1553.

6-Acetyl-2-(4-chlorophenyl)-5,6-dihydro-4*H*-benzo[*b*][1,2,4]tri-azolo[1,5*d*][1,4]diazepine (13e)

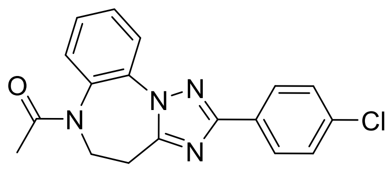

White solid. Yield: 126.0 mg, 31%. Mp: 204-205 °C. ^{1}H NMR (400 MHz, DMSO-d_6): δ 8.11 – 8.05 (m, 3H), 7.67 – 7.56 (m, 4H), 7.55 – 7.49 (m, 1H), 4.82 – 4.71 (m, 1H), 3.51 – 3.33 (m, 2H), 3.19 (dt, J = 10.8, 5.7 Hz, 1H), 1.78 (s, 3H). ^{13}C NMR (101 MHz, DMSO-d_6): δ 168.14, 159.05, 154.91, 133.68, 133.38, 133.33, 128.84, 128.56, 128.44, 128.40, 127.88, 127.17, 122.94, 44.25, 25.40, 21.71. HRMS (ESI): m/z [M + H]$^+$ calcd for $C_{18}H_{15}ClN_4O$: 339.0982; found 339.0987.

3. Copies of NMR spectra

^{1}H NMR spectrum of **6a**

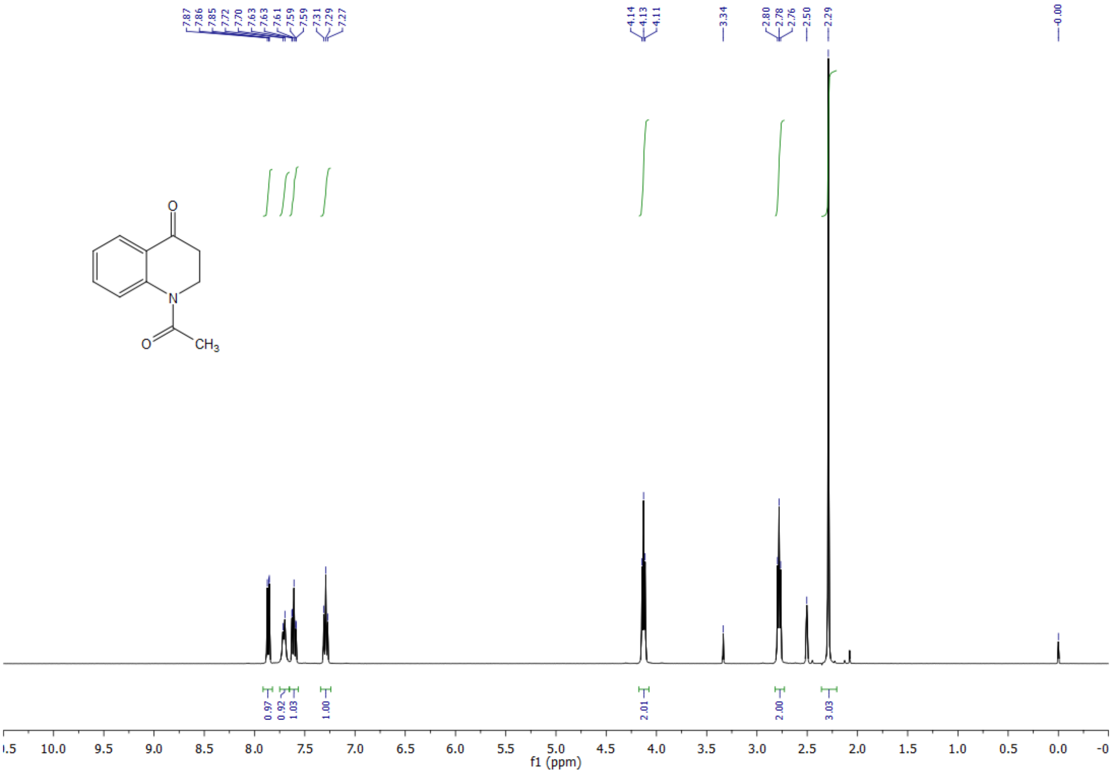

^{13}C NMR spectrum of **6a**

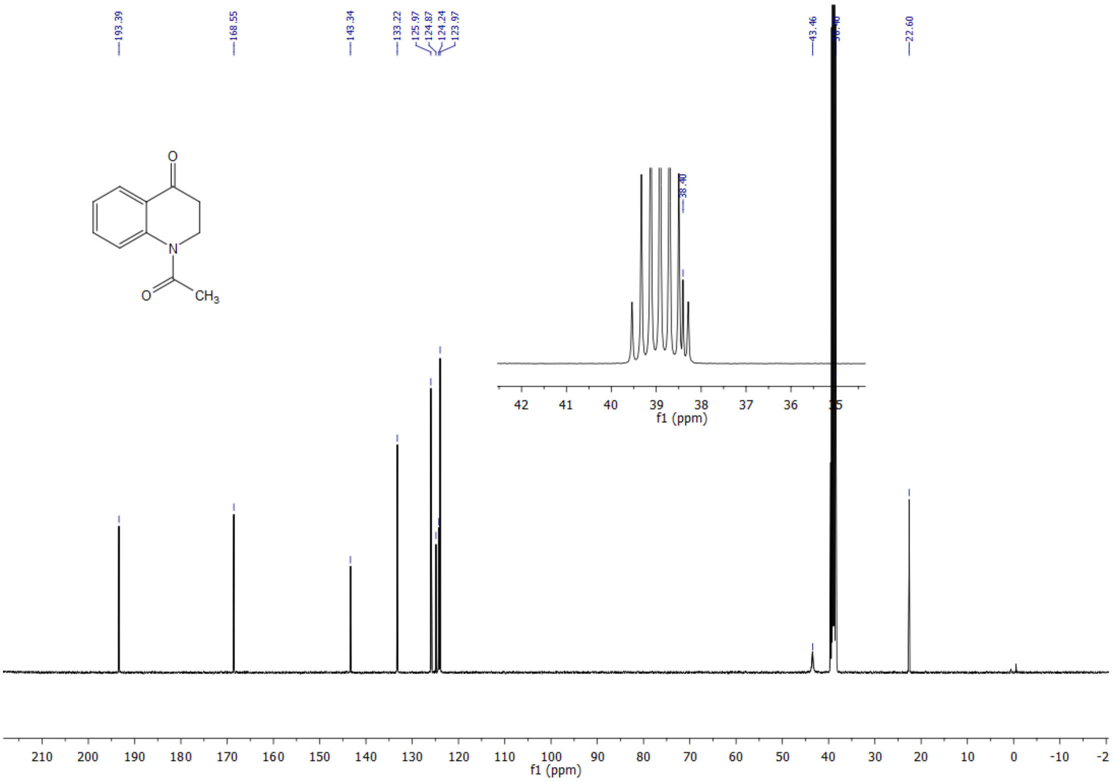

^{1}H NMR spectrum of **6b**

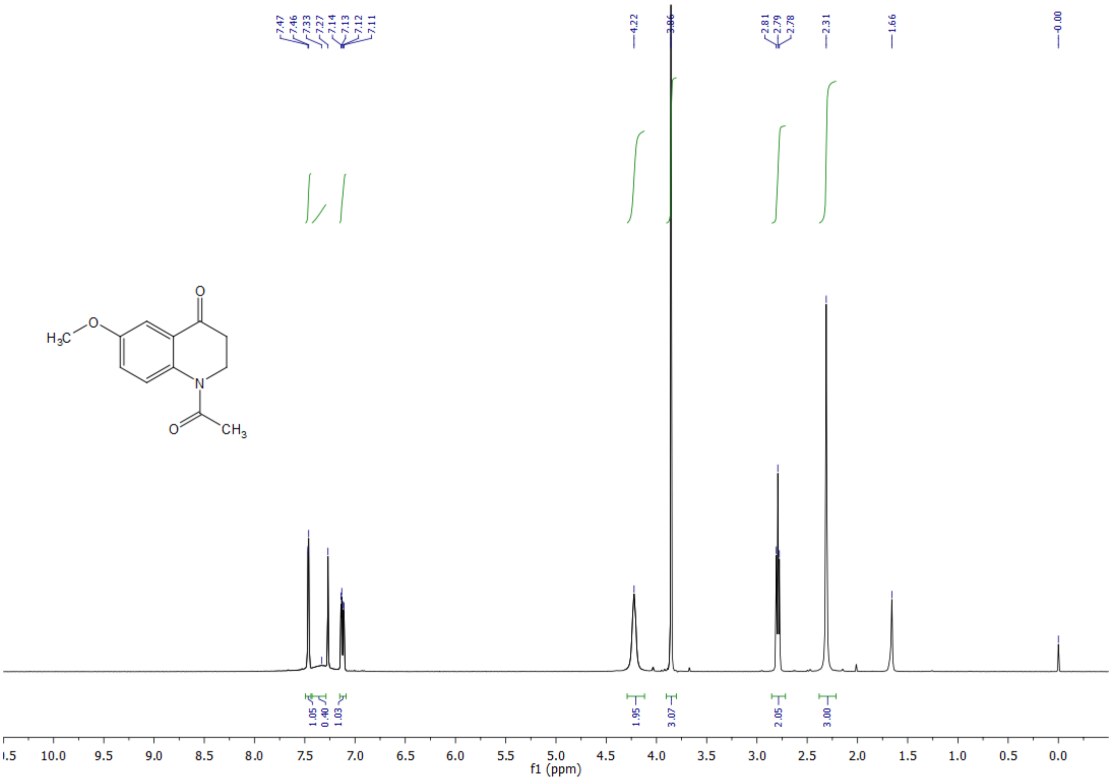

^{13}C NMR spectrum of **6b**

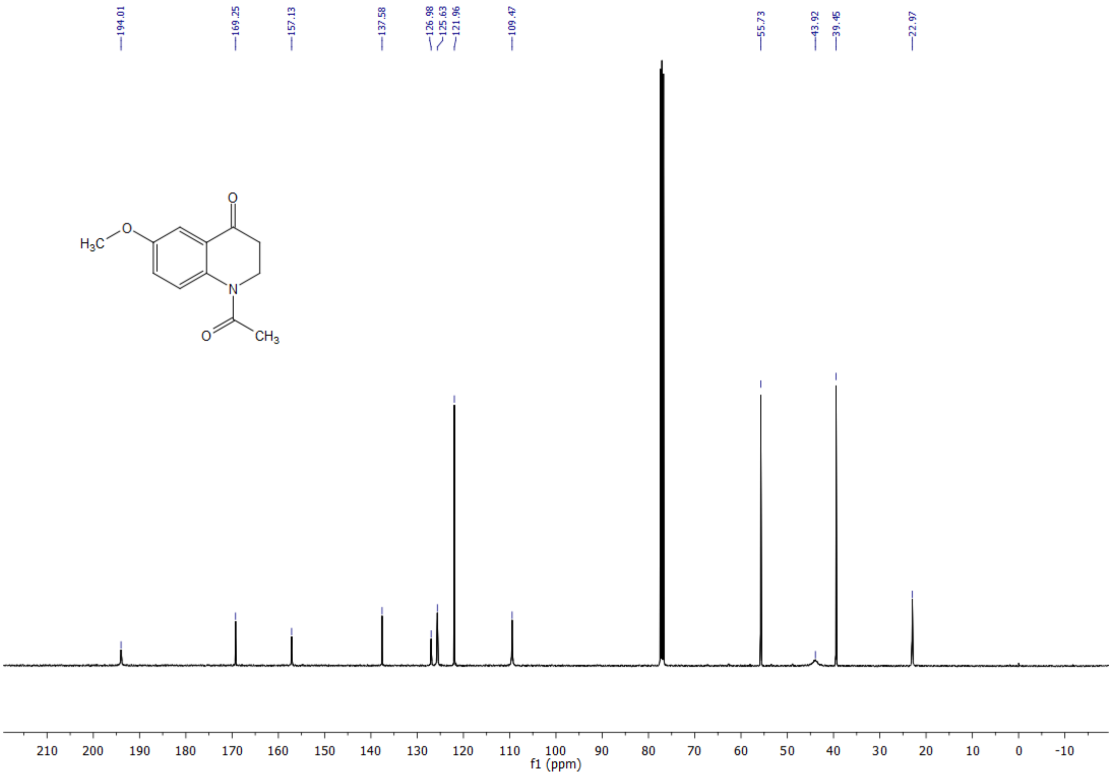

^{1}H NMR spectrum of **6c**

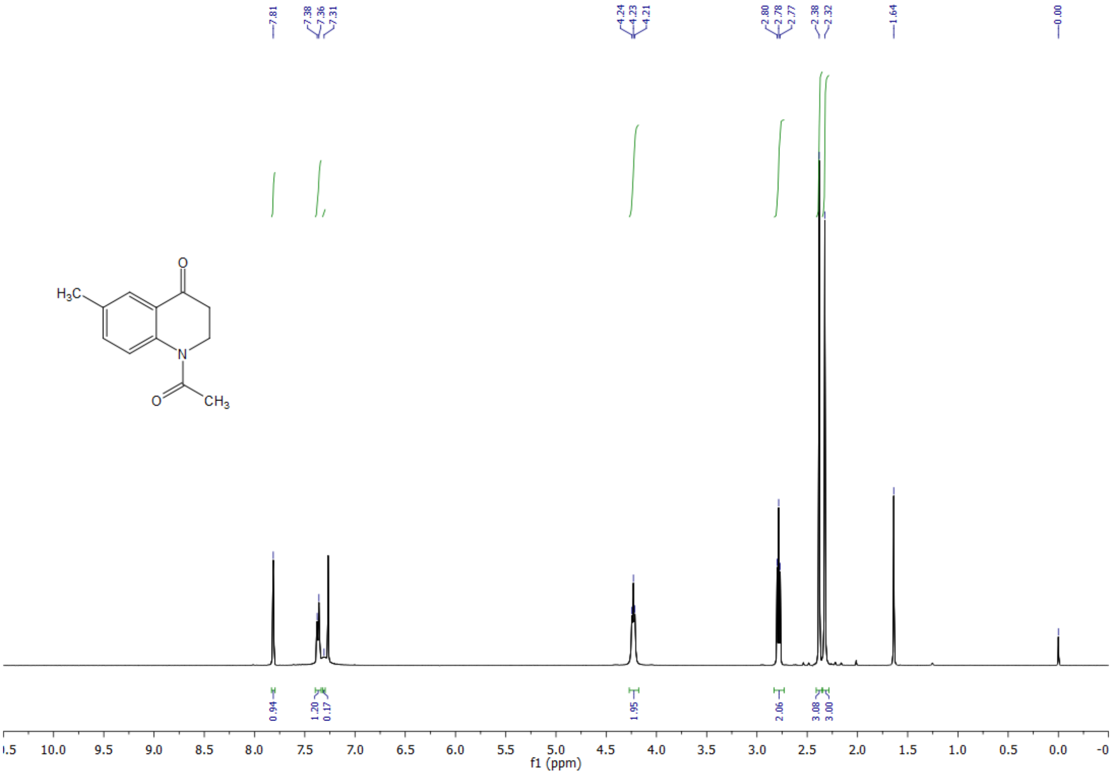

^{13}C NMR spectrum of **6c**

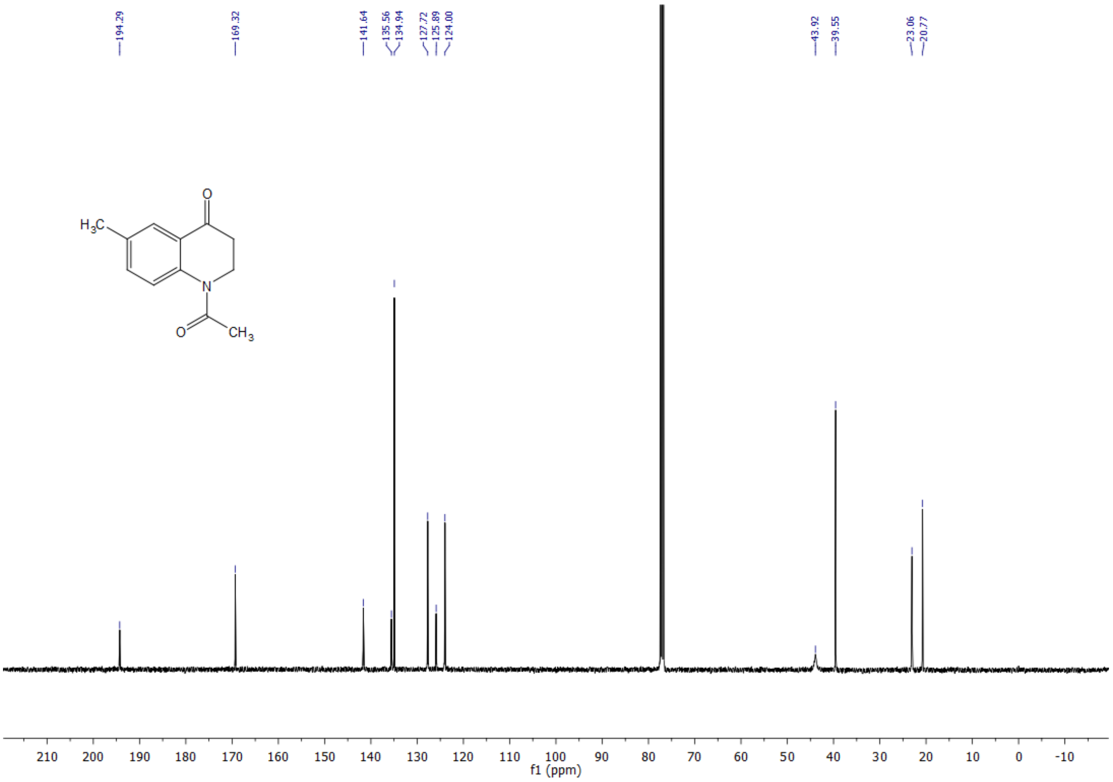

^{1}H NMR spectrum of **6d**

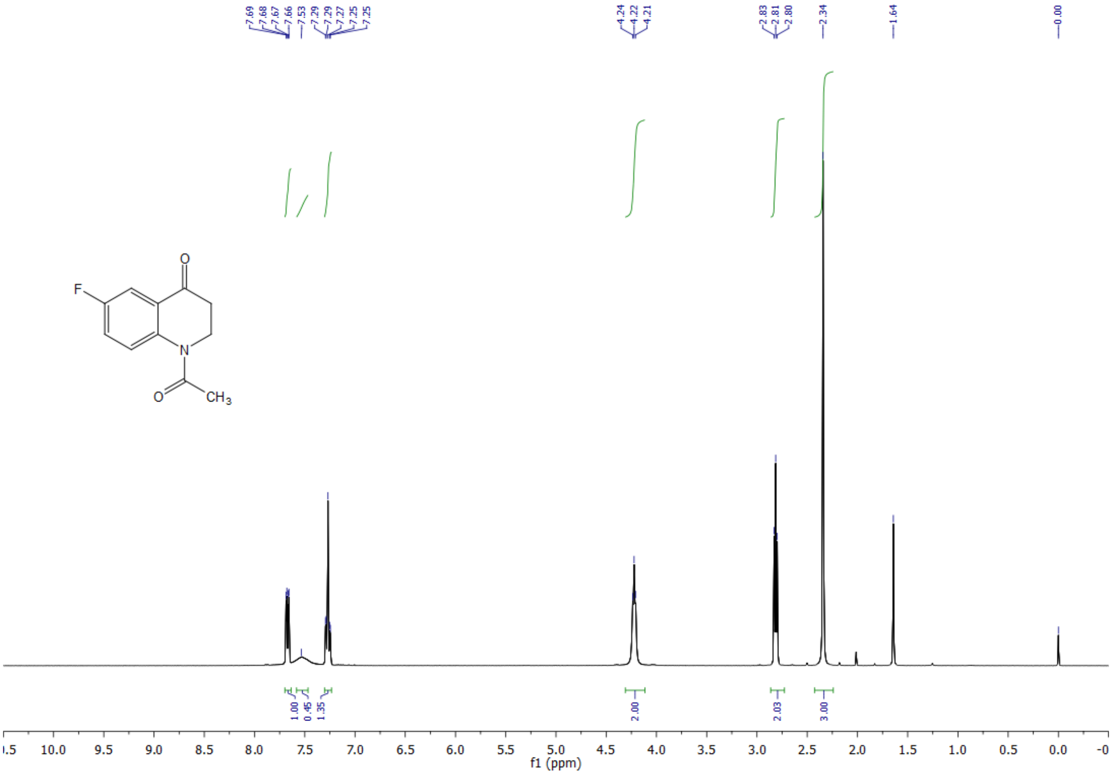

^{13}C NMR spectrum of **6d**

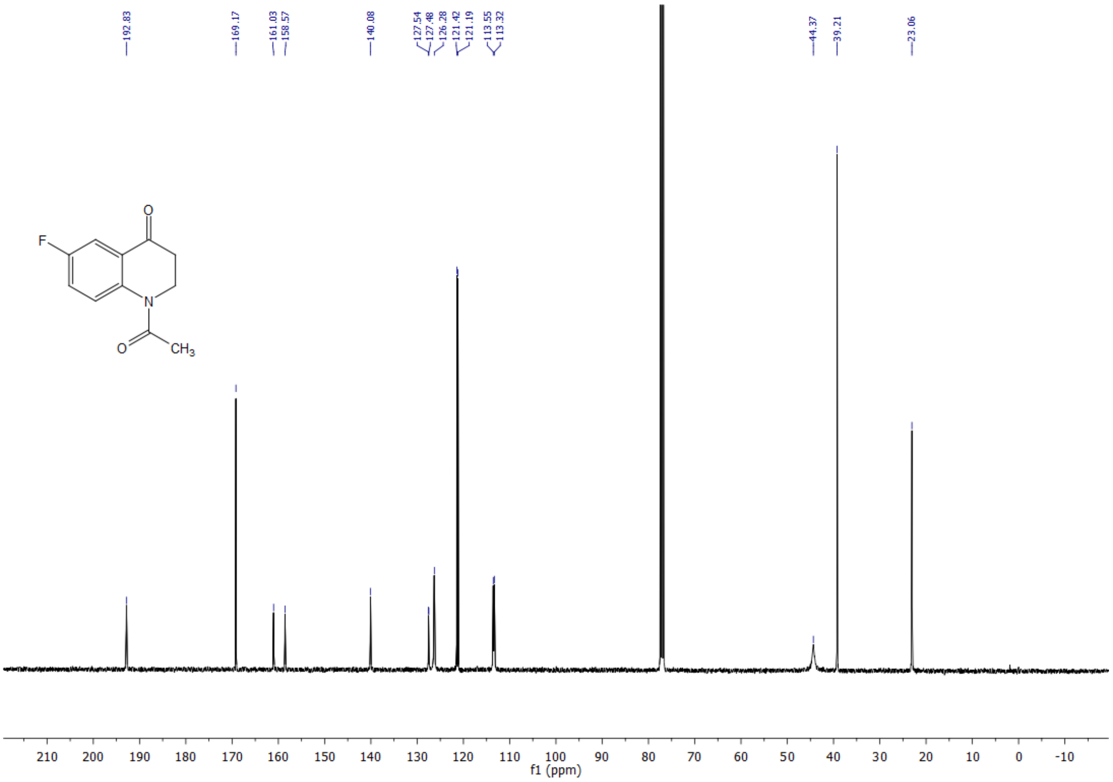

^{1}H NMR spectrum of **6e**

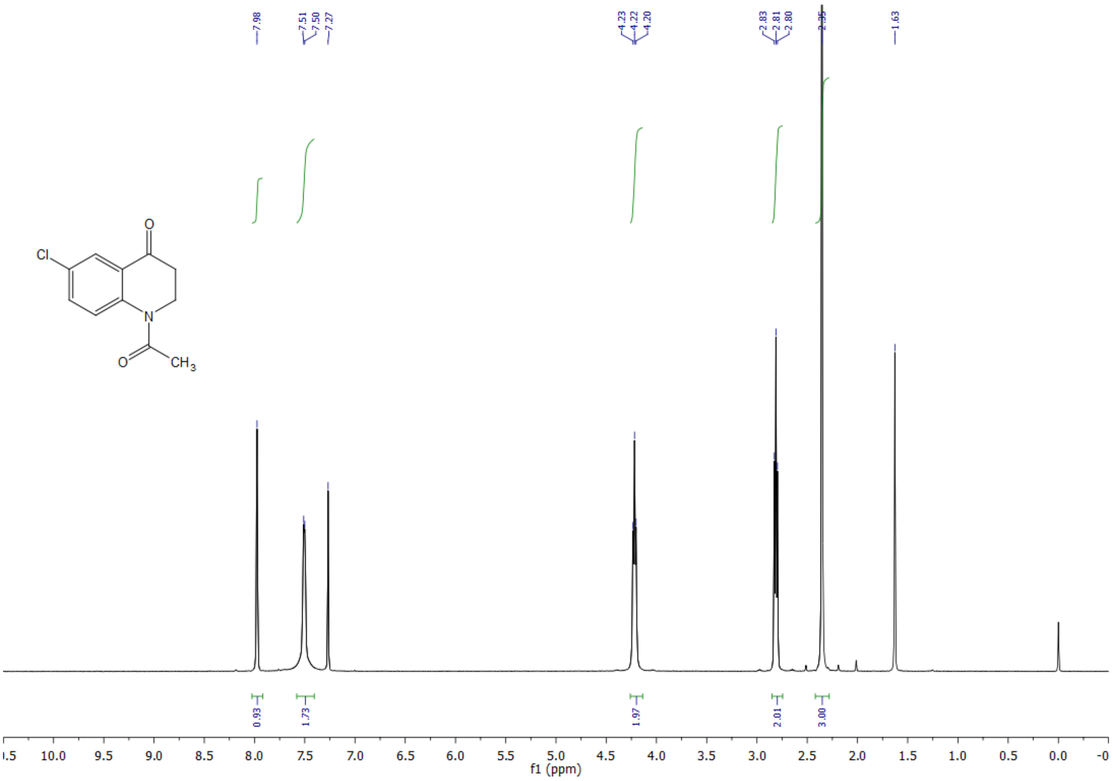

^{13}C NMR spectrum of **6e**

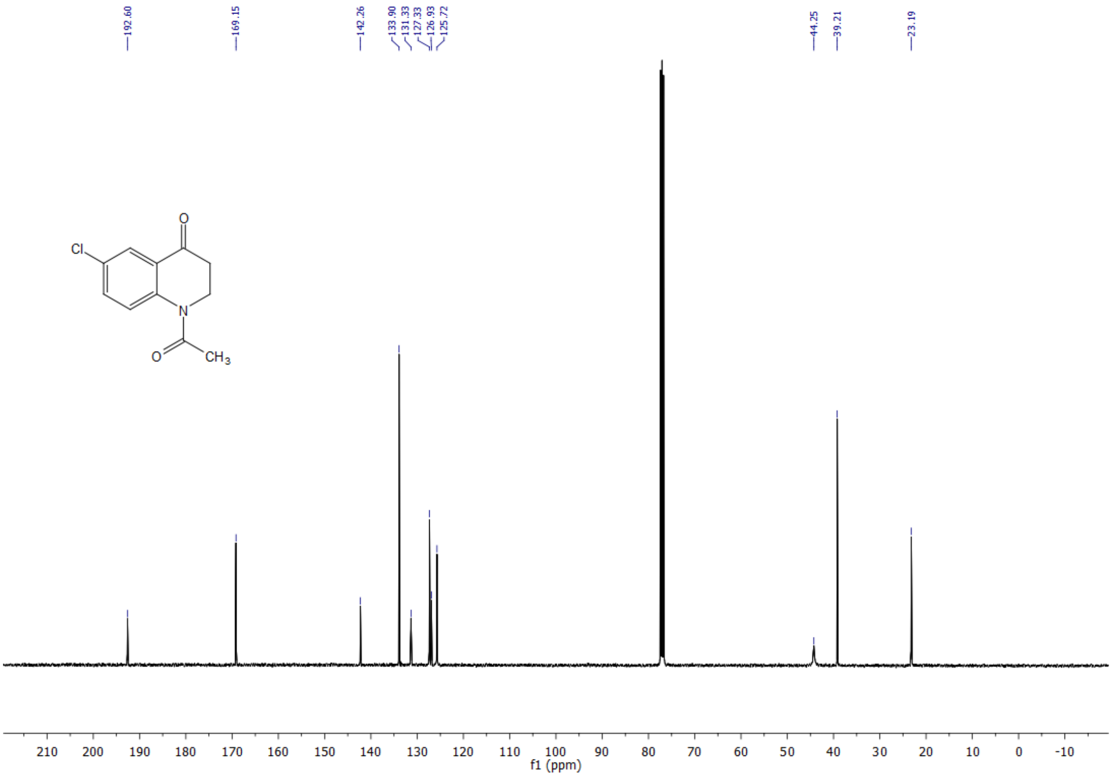

^{1}H NMR spectrum of **6f**

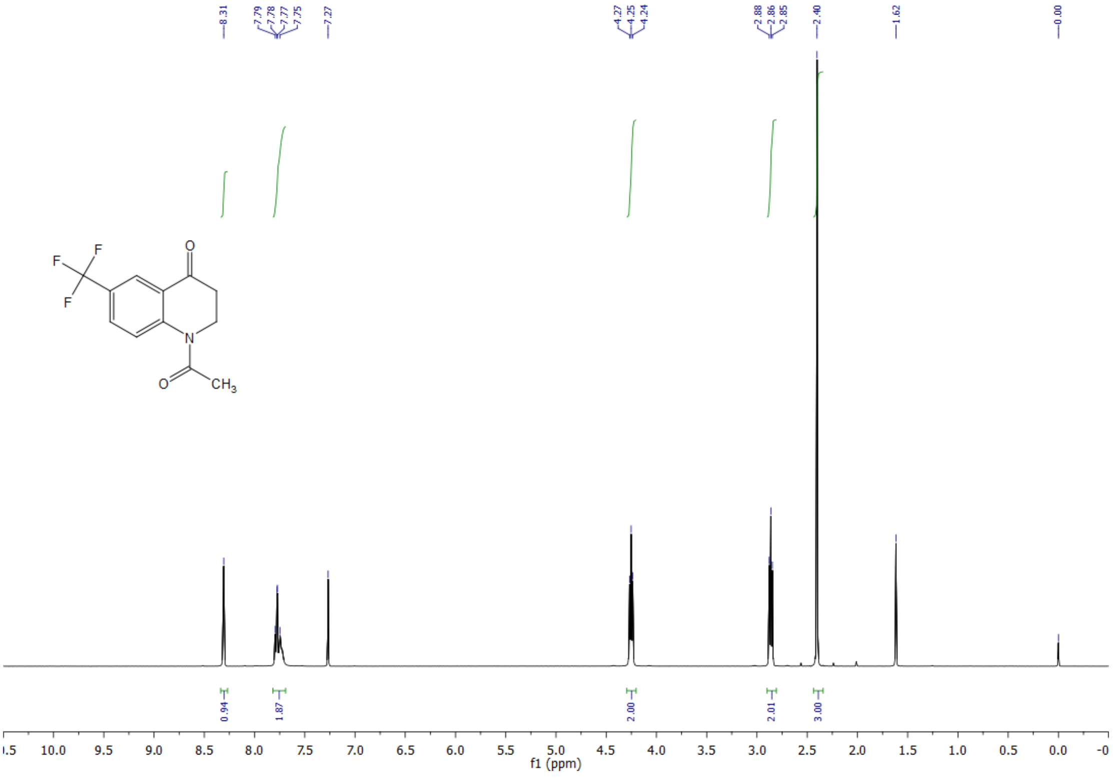

^{13}C NMR spectrum of **6f**

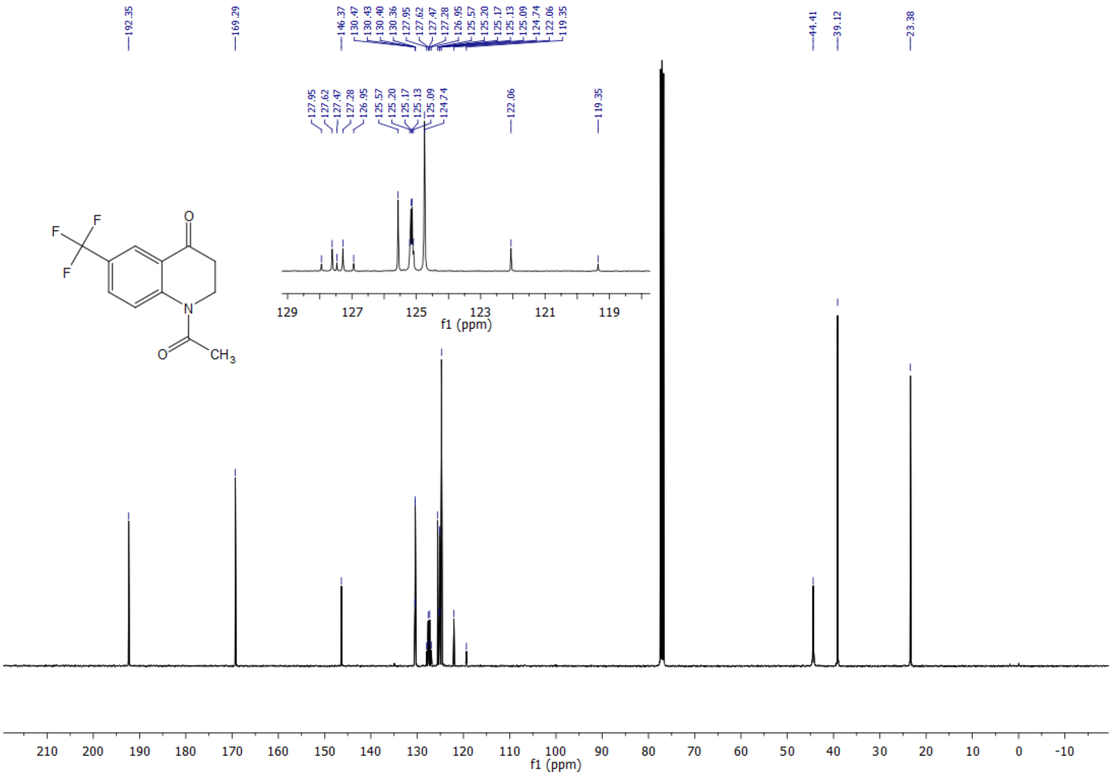

^{1}H NMR spectrum of **6g**

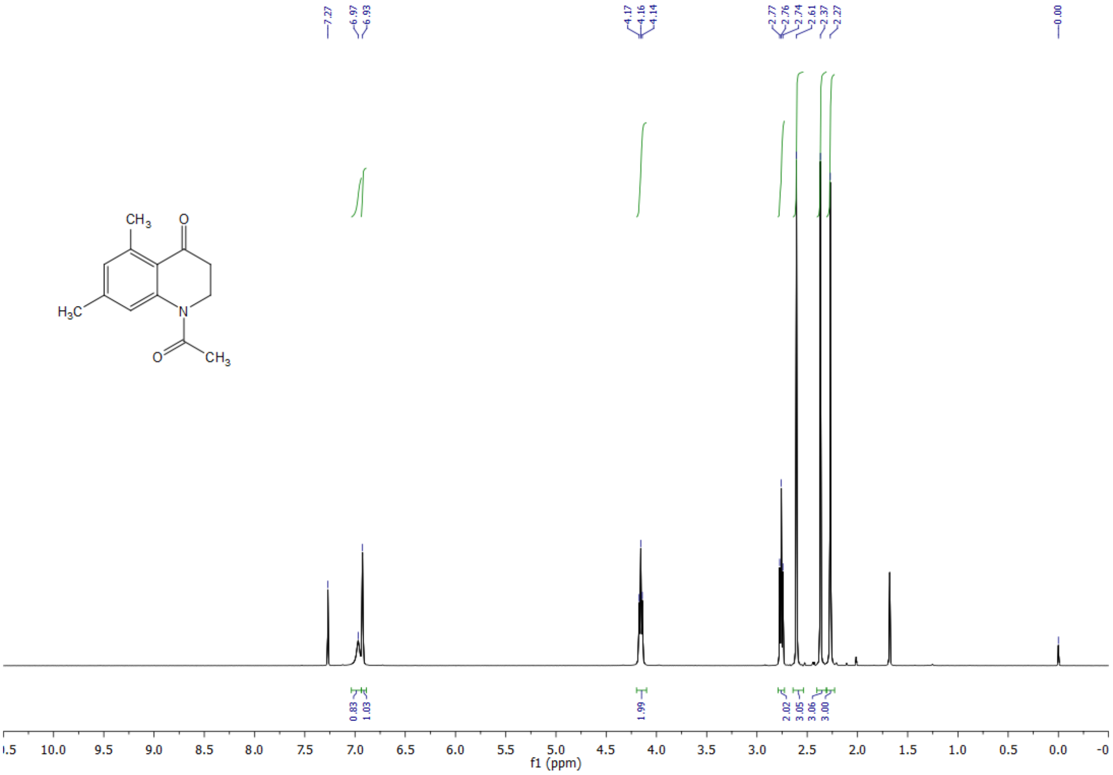

^{13}C NMR spectrum of **6g**

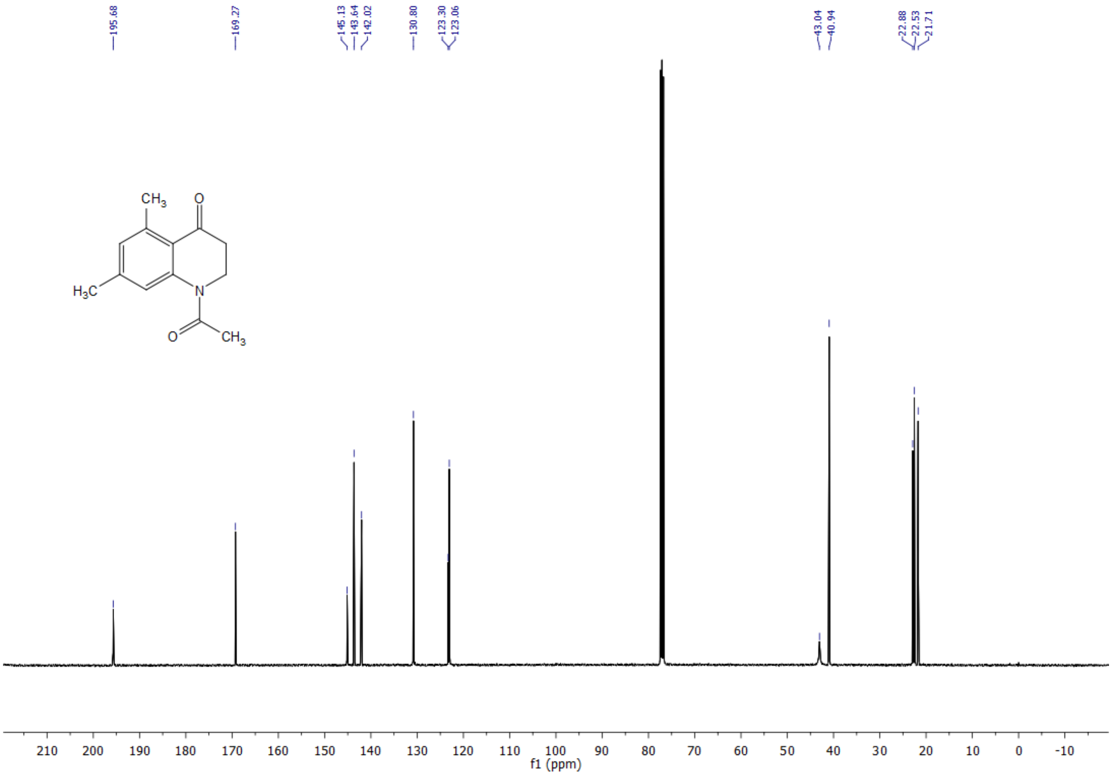

^{1}H NMR spectrum of **7a**

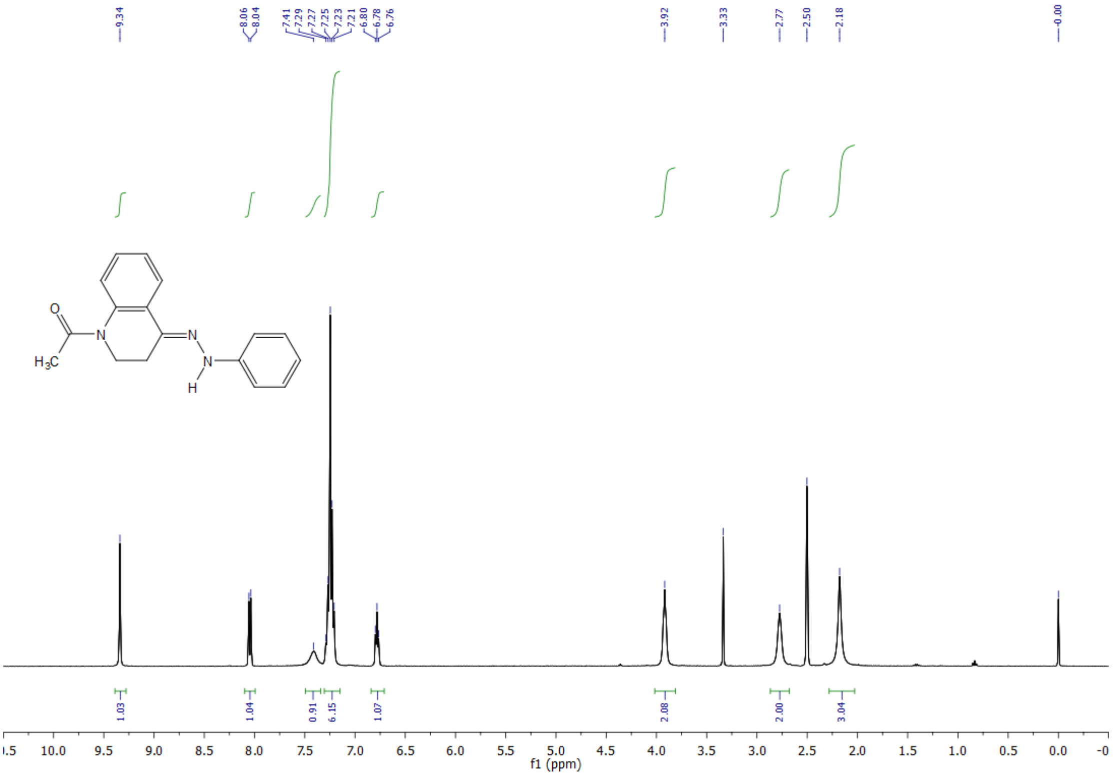

^{13}C NMR spectrum of **7a**

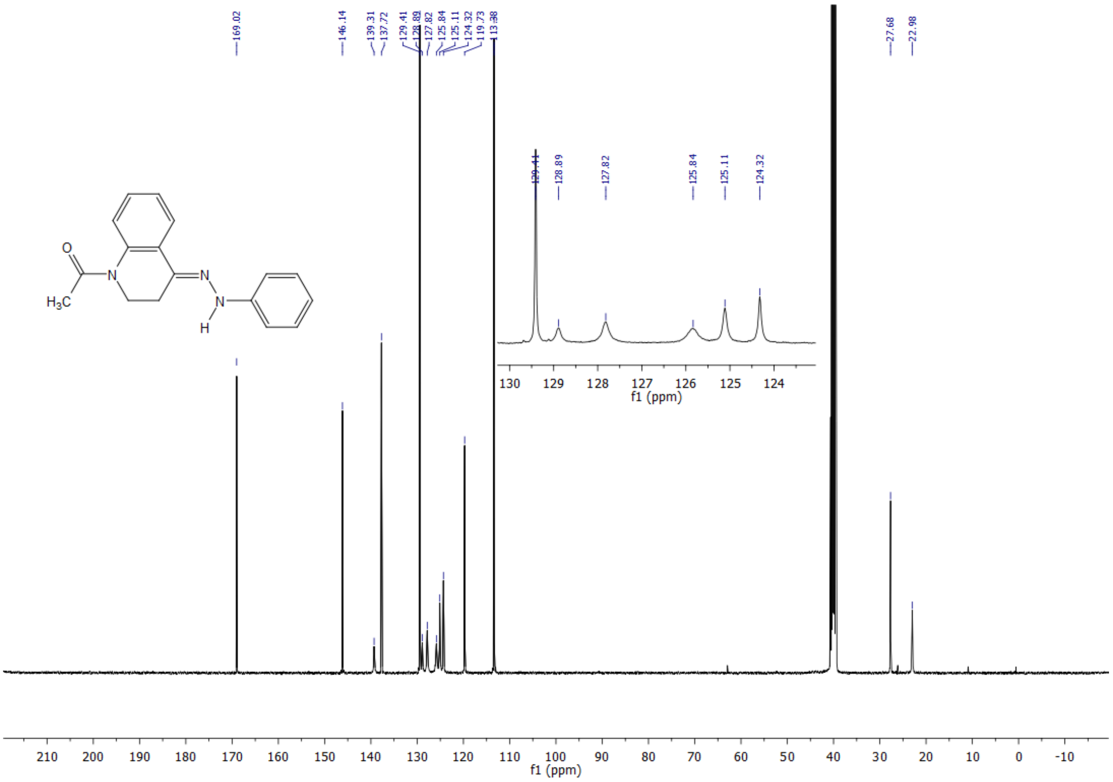

^{1}H NMR spectrum of **7b**

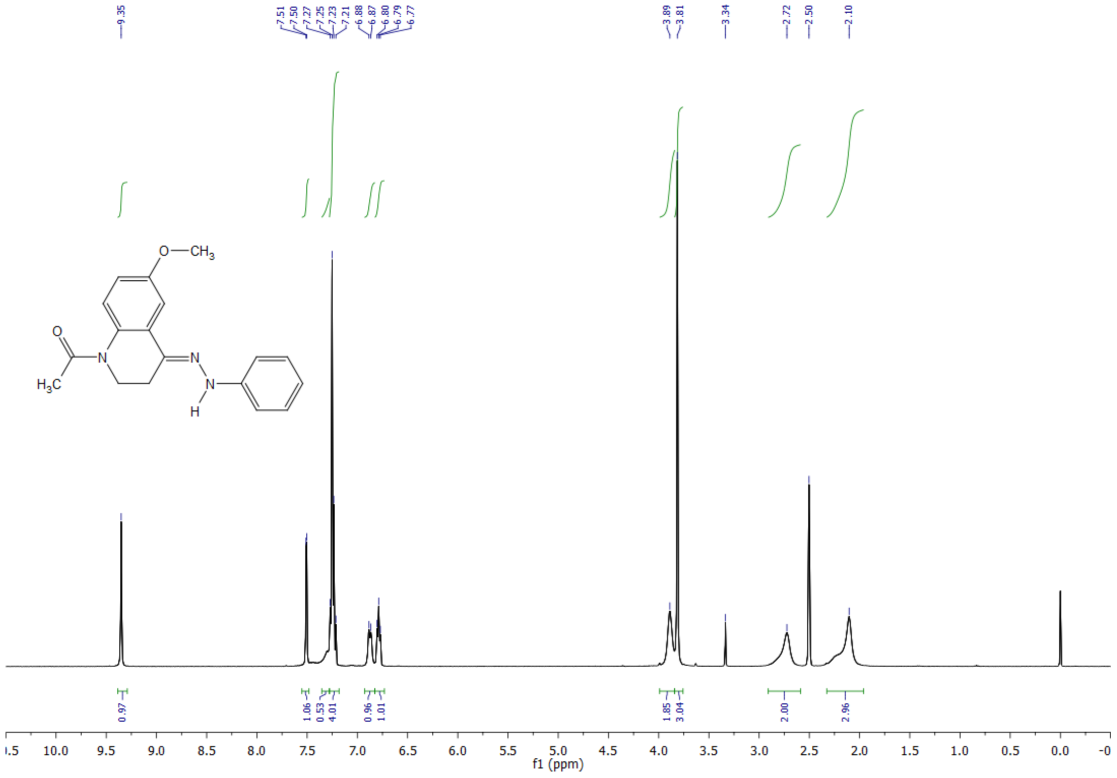

^{13}C NMR spectrum of **7b**

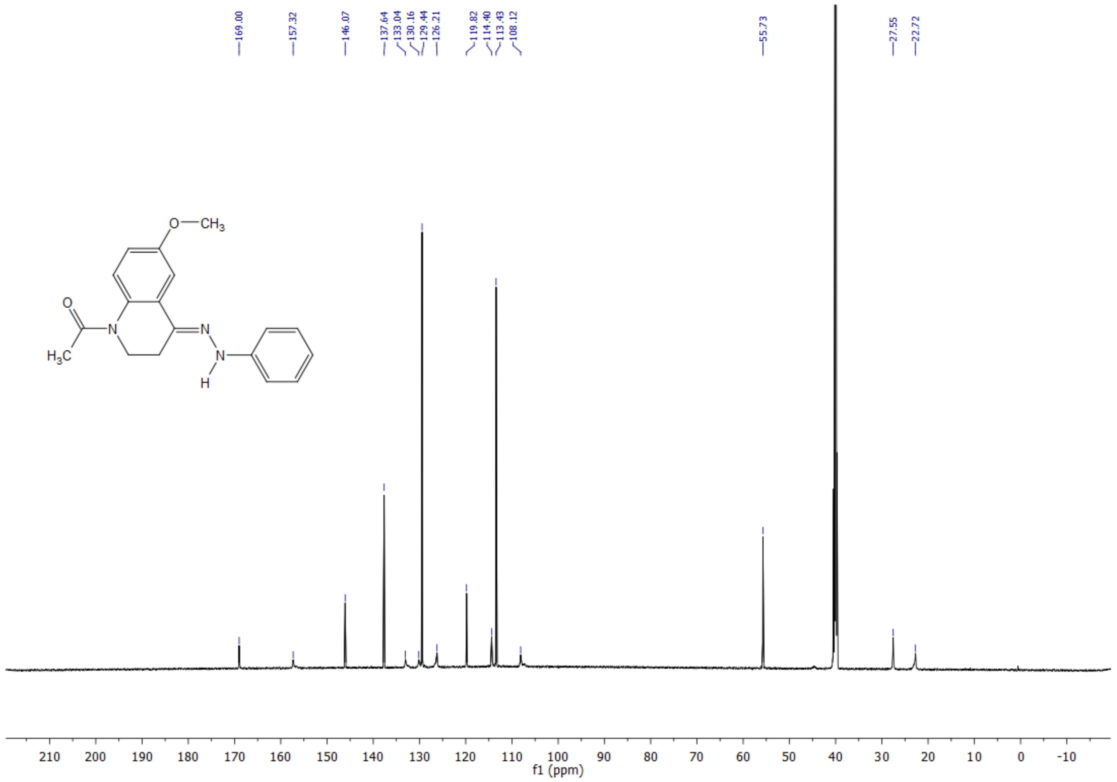

^{1}H NMR spectrum of **7c**

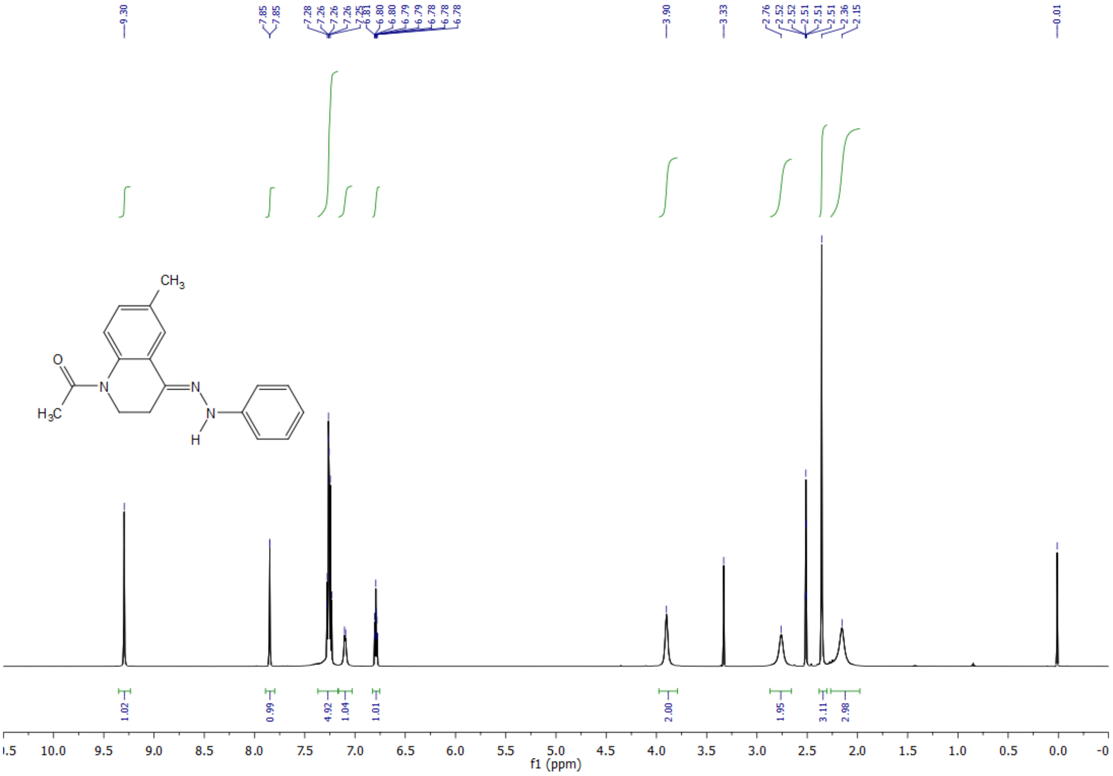

^{13}C NMR spectrum of **7c**

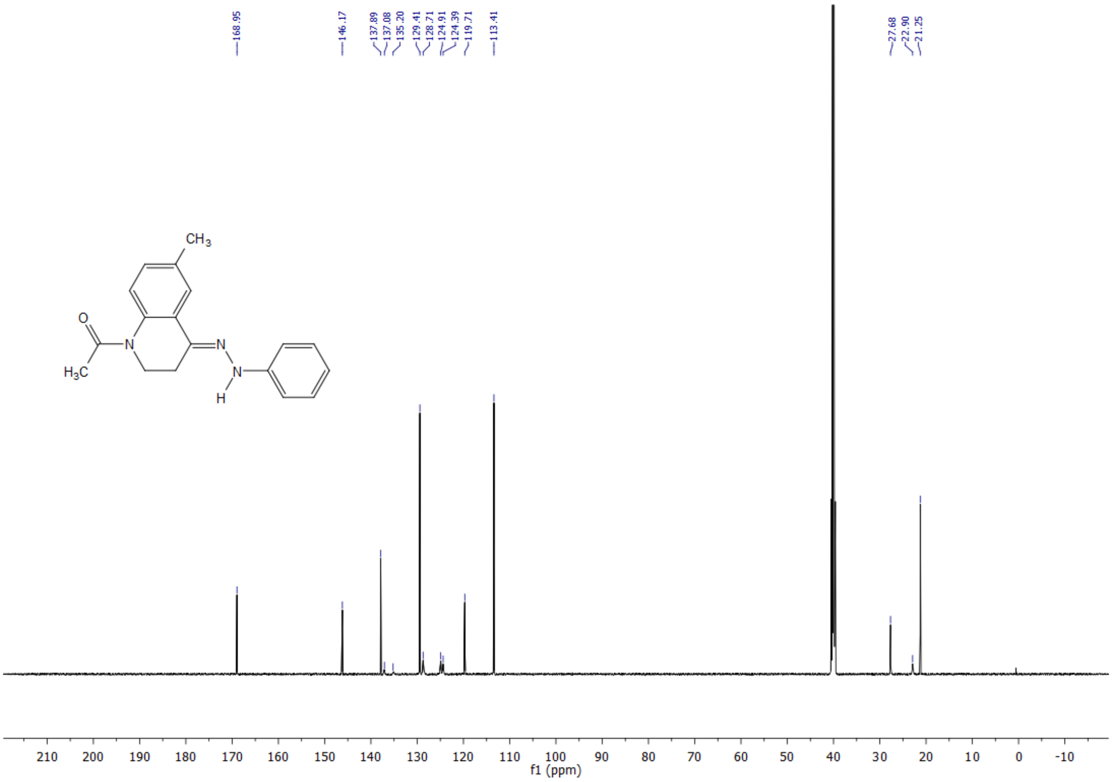

^{1}H NMR spectrum of **7d**

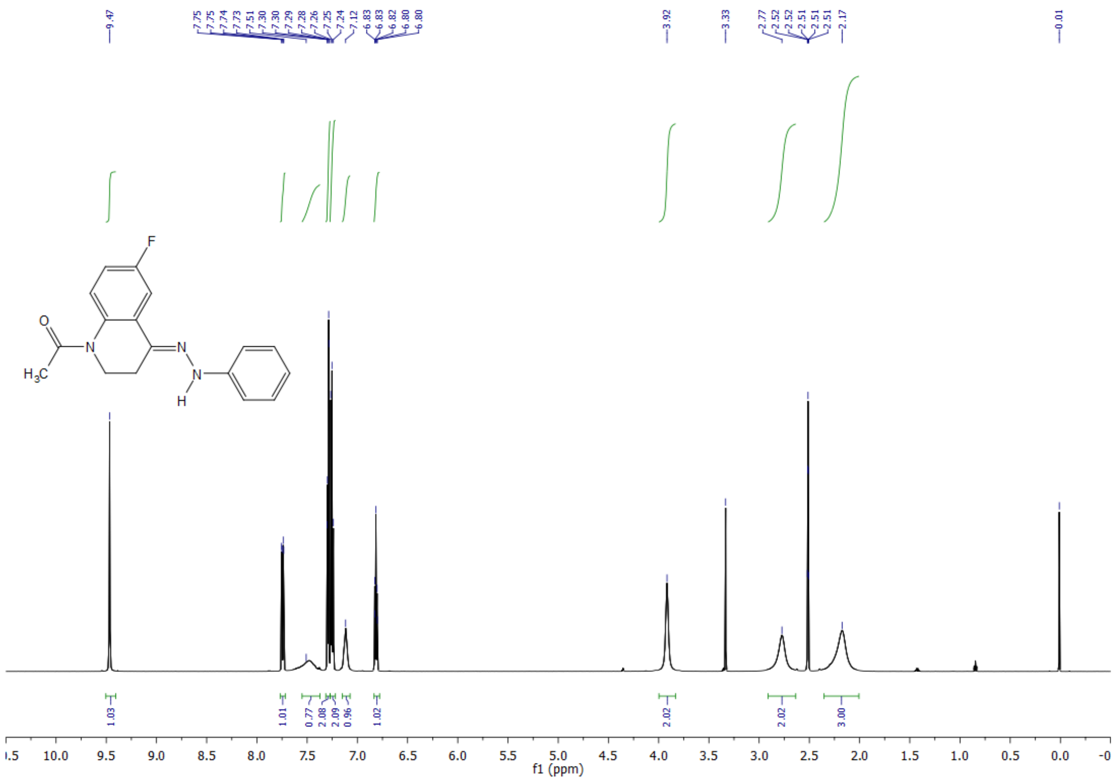

^{13}C NMR spectrum of **7d**

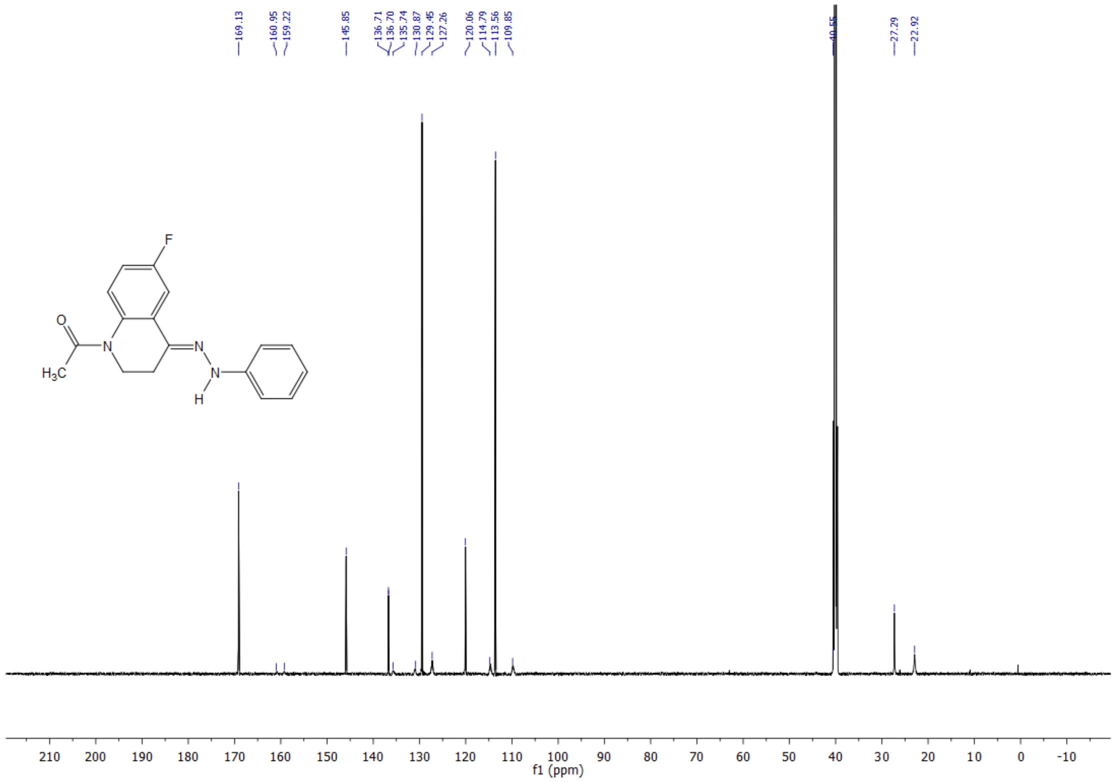

^{1}H NMR spectrum of **7e**

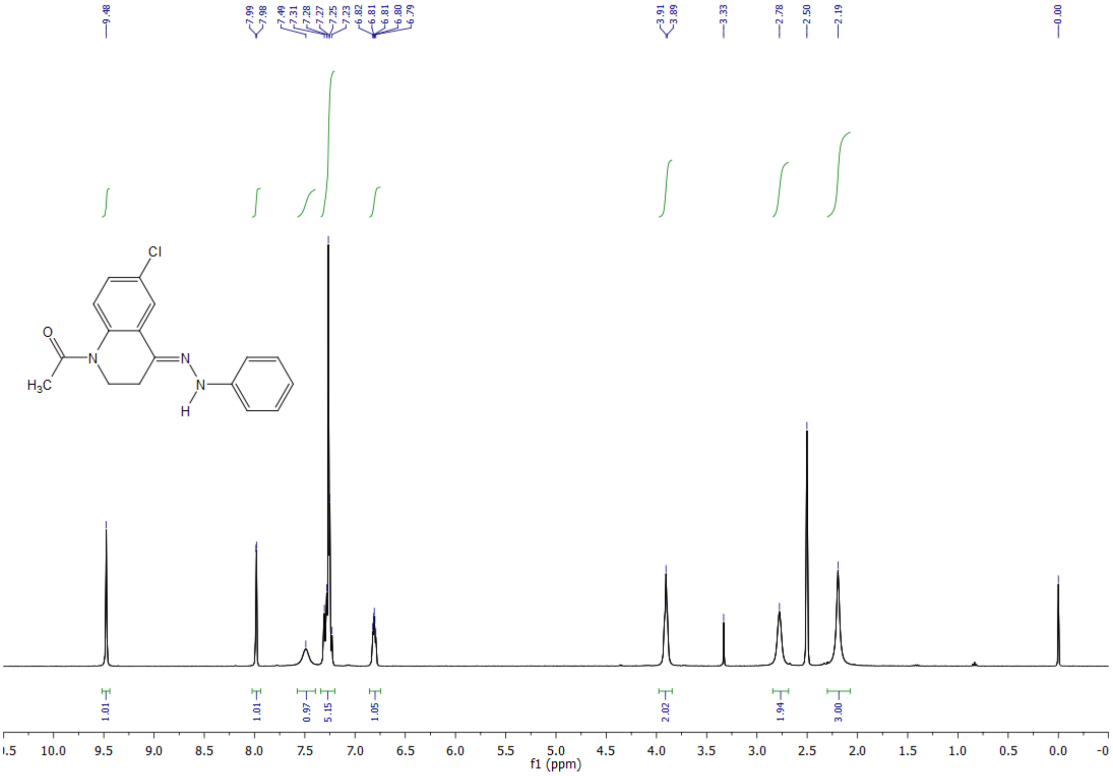

^{13}C NMR spectrum of **7e**

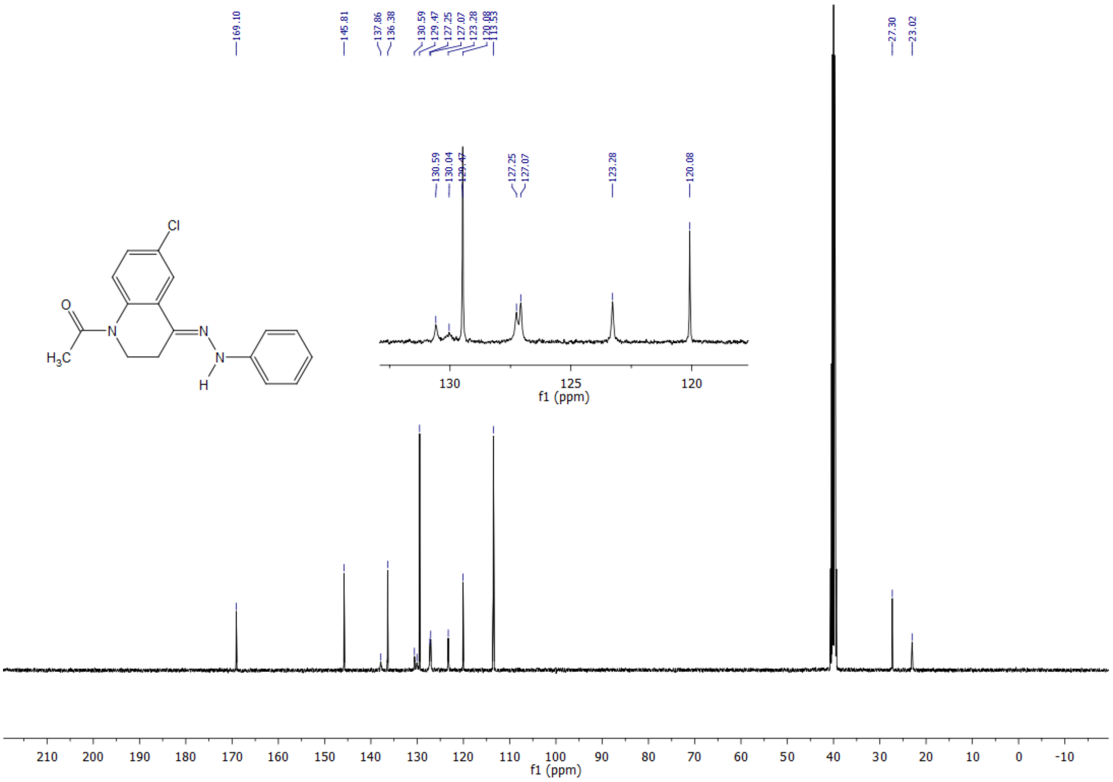

^{1}H NMR spectrum of **7f**

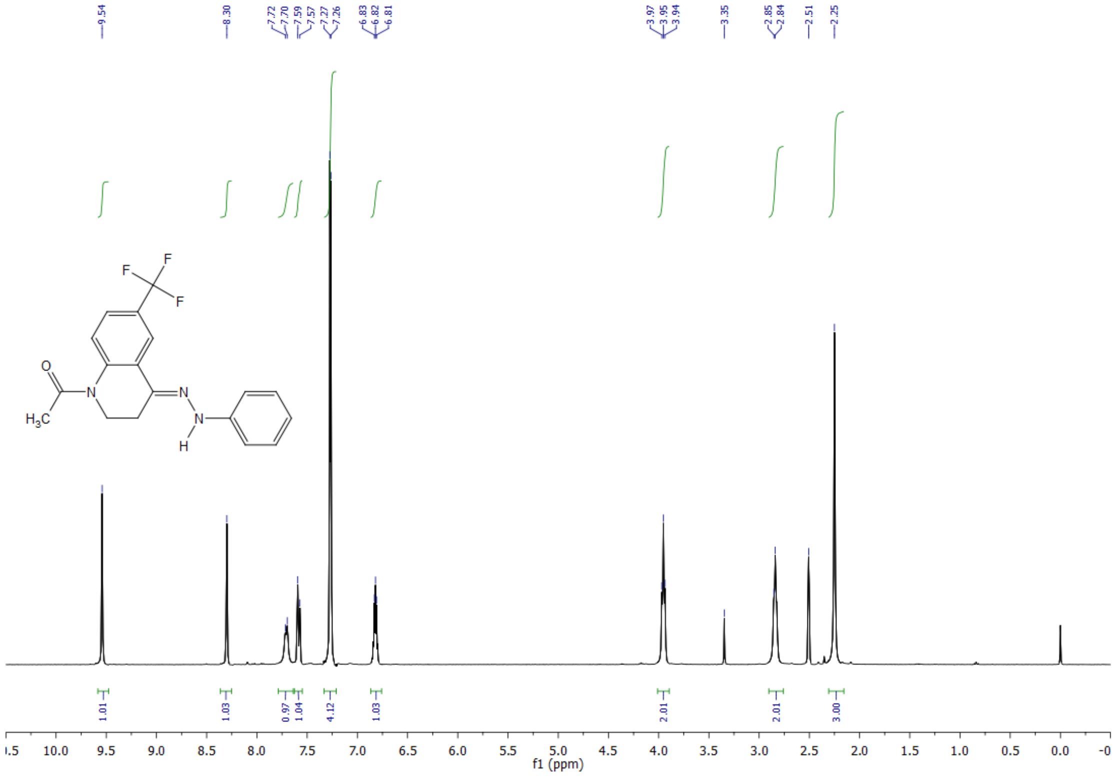

^{13}C NMR spectrum of **7f**

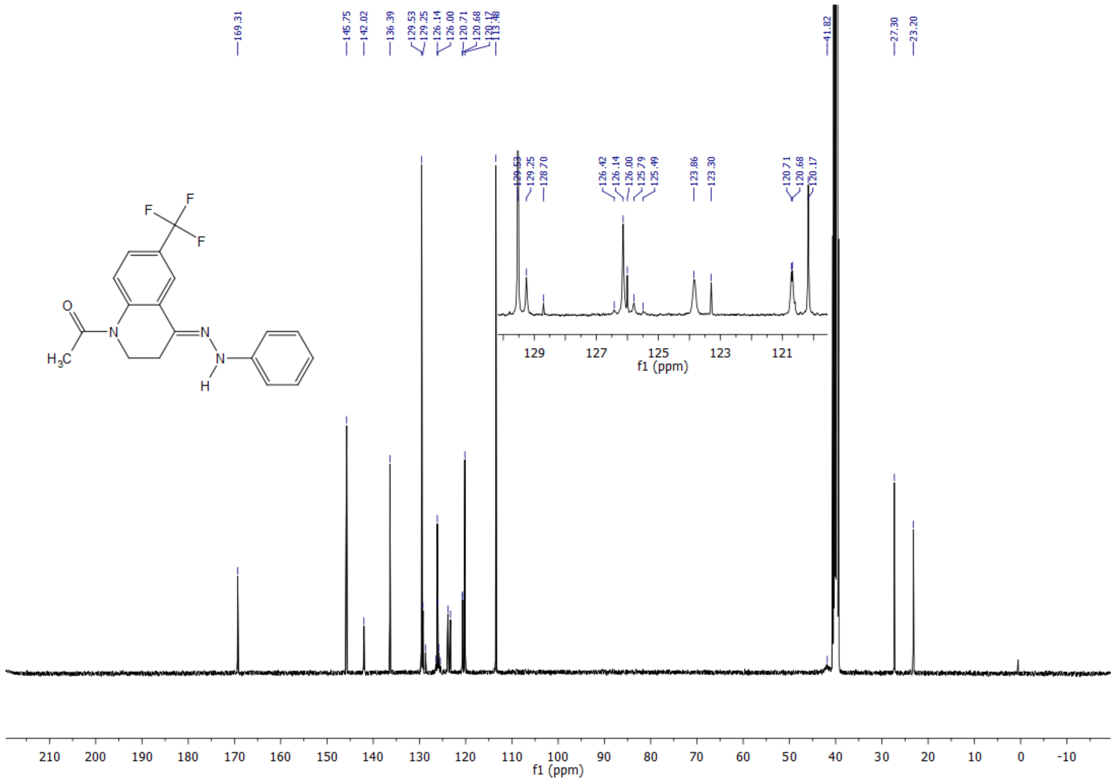

^{1}H NMR spectrum of **7g**

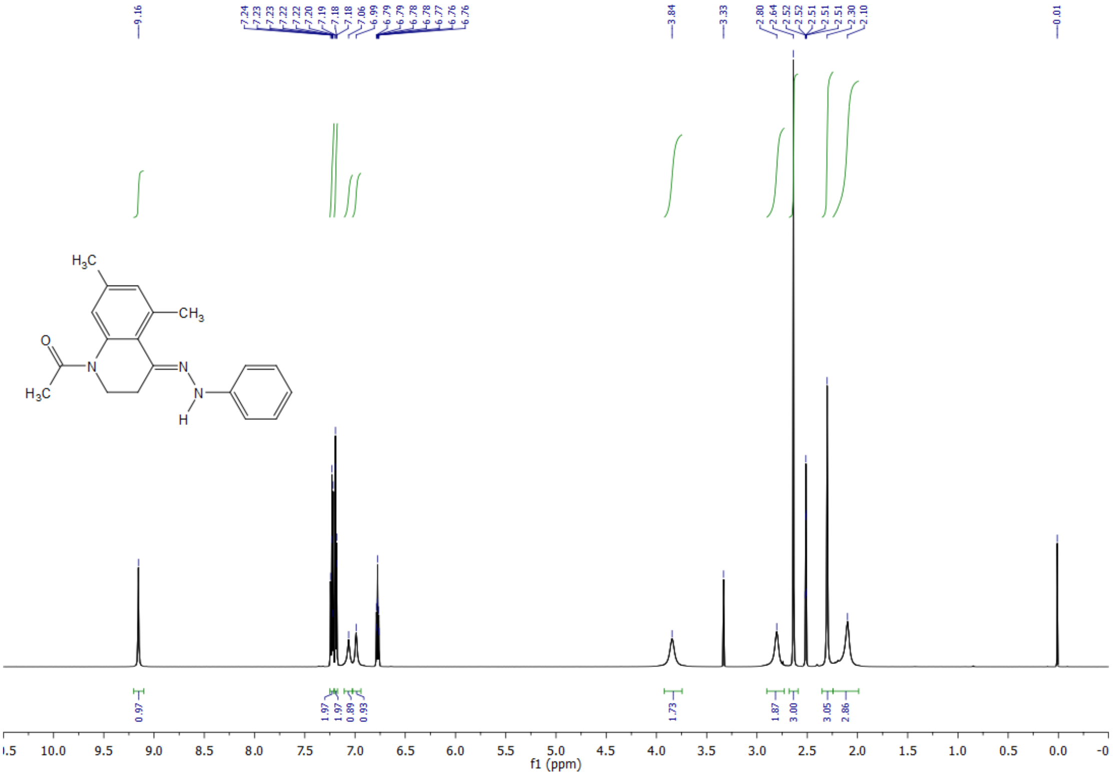

^{13}C NMR spectrum of **7g**

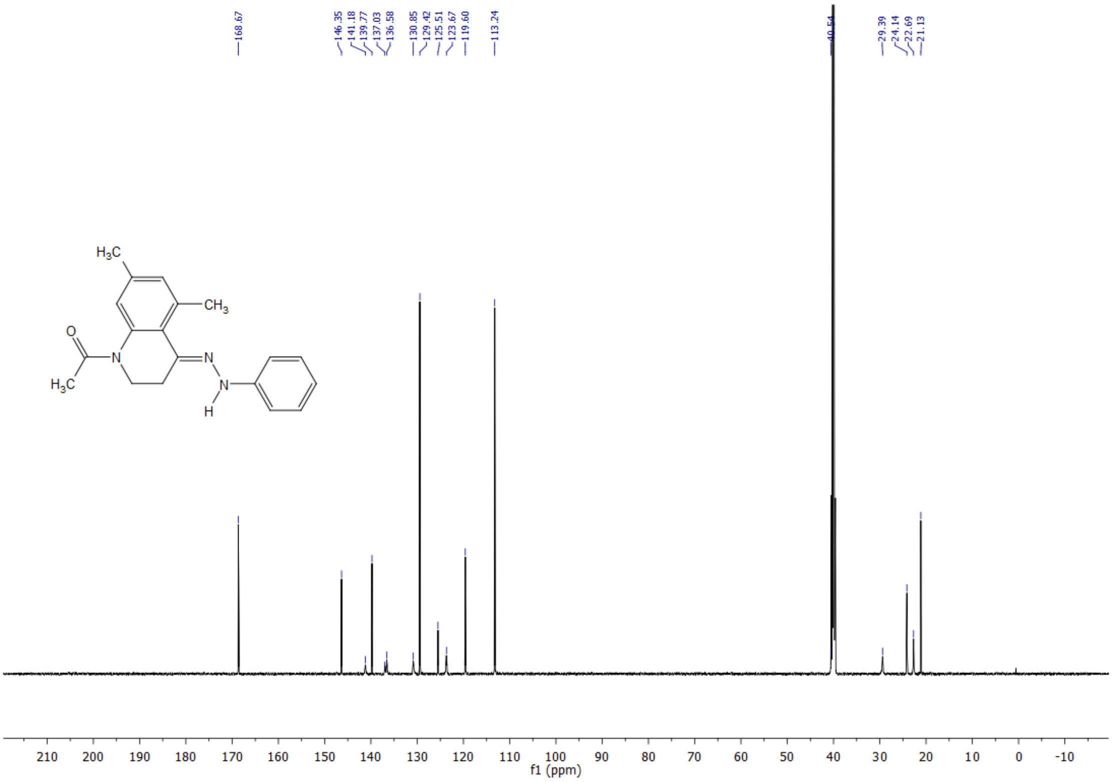

^{1}H NMR spectrum of **8a**

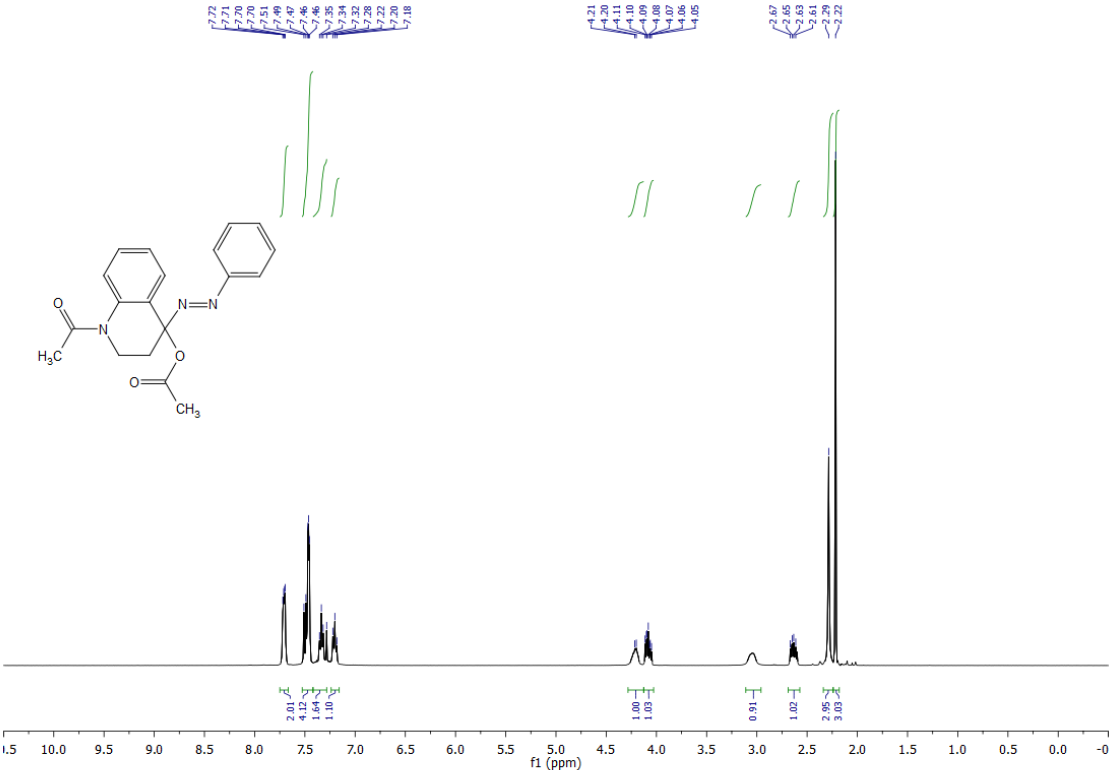

^{13}C NMR spectrum of **8a**

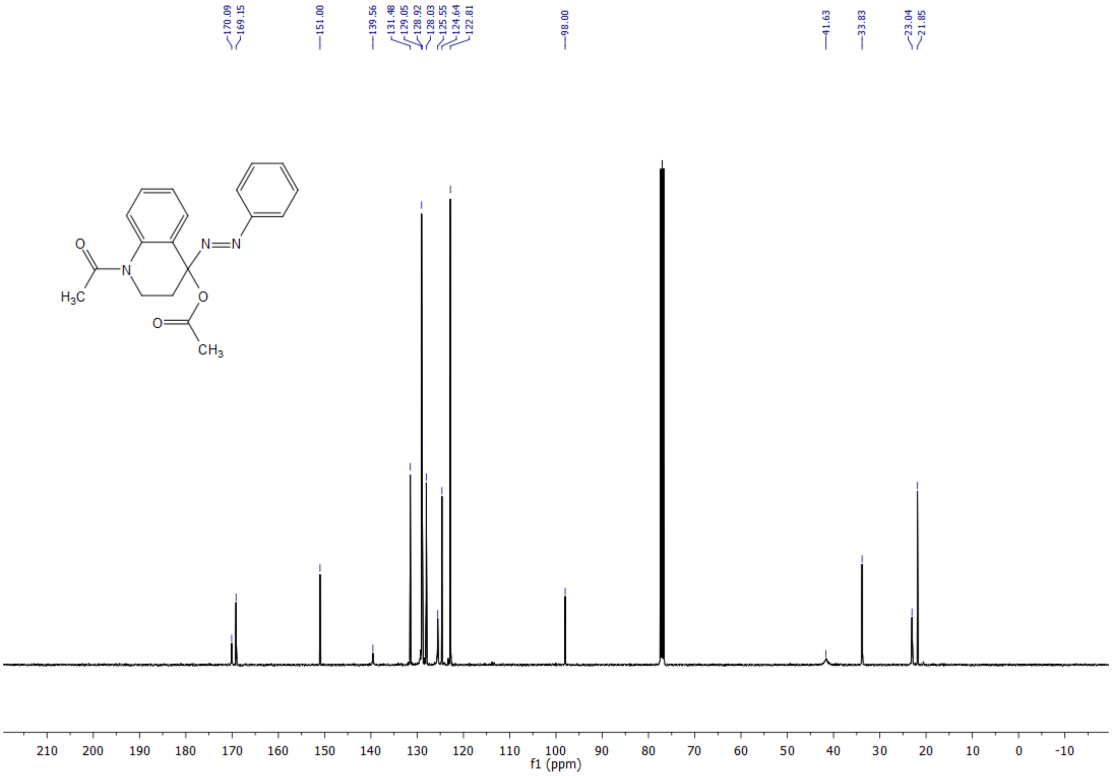

^{1}H NMR spectrum of **8b**

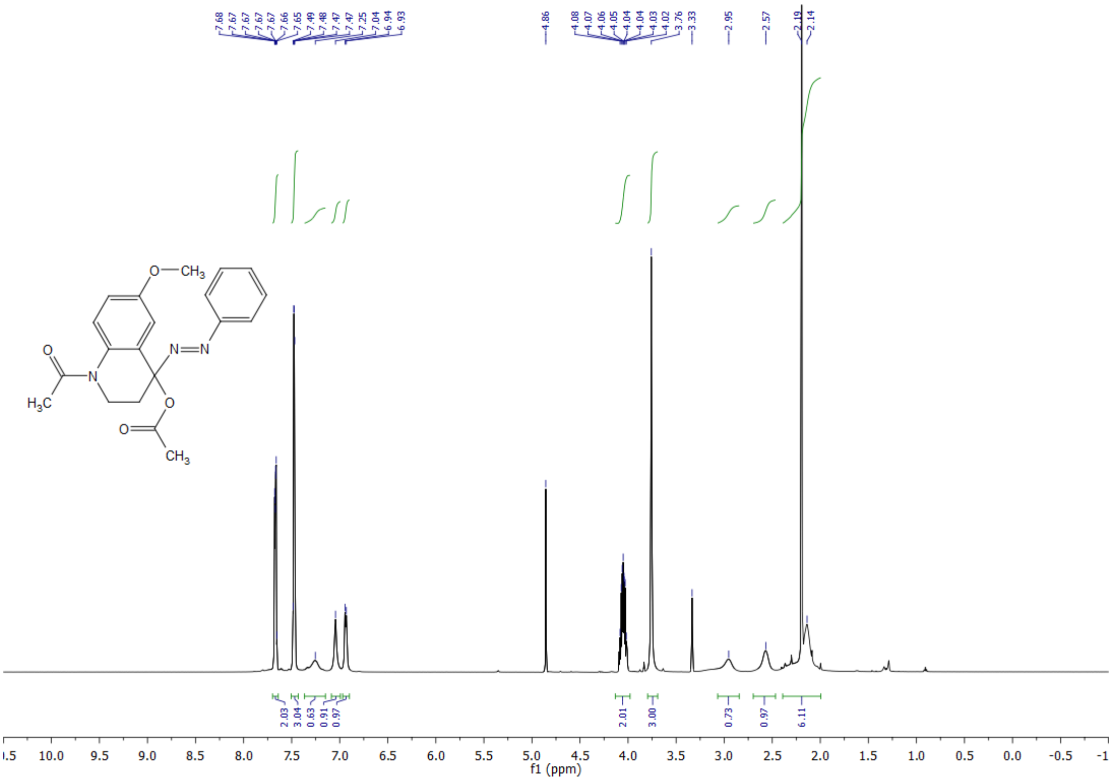

^{13}C NMR spectrum of **8b**

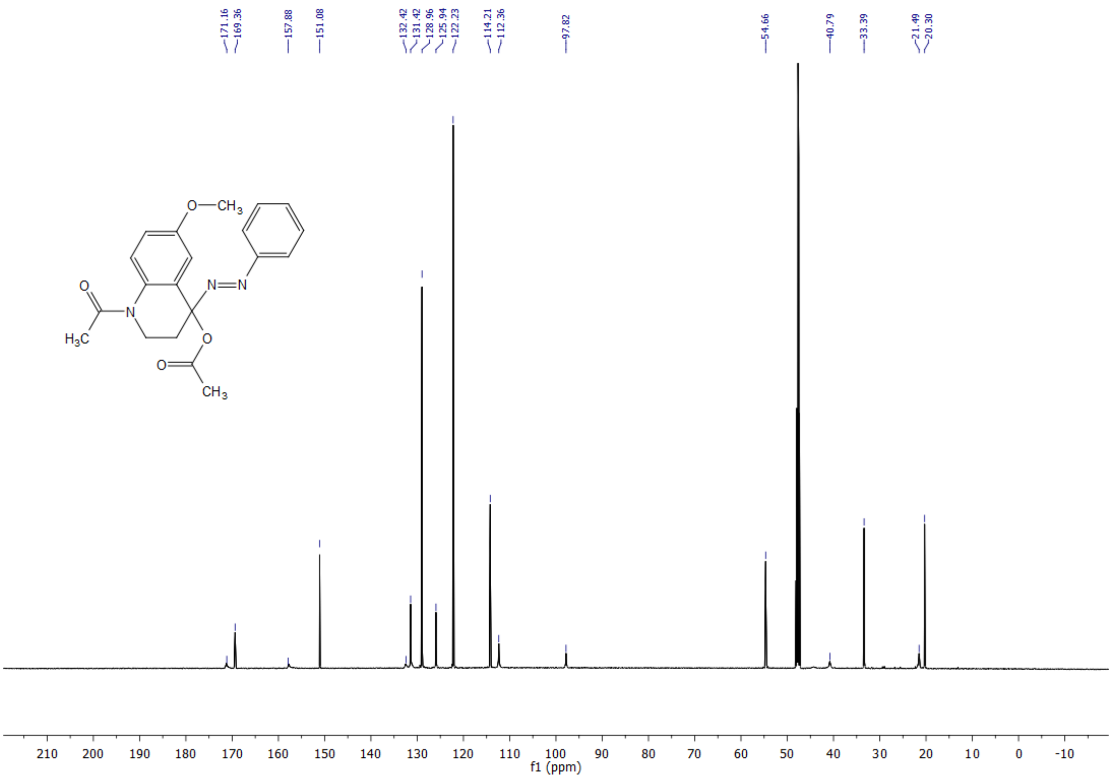

^{1}H NMR spectrum of **8c**

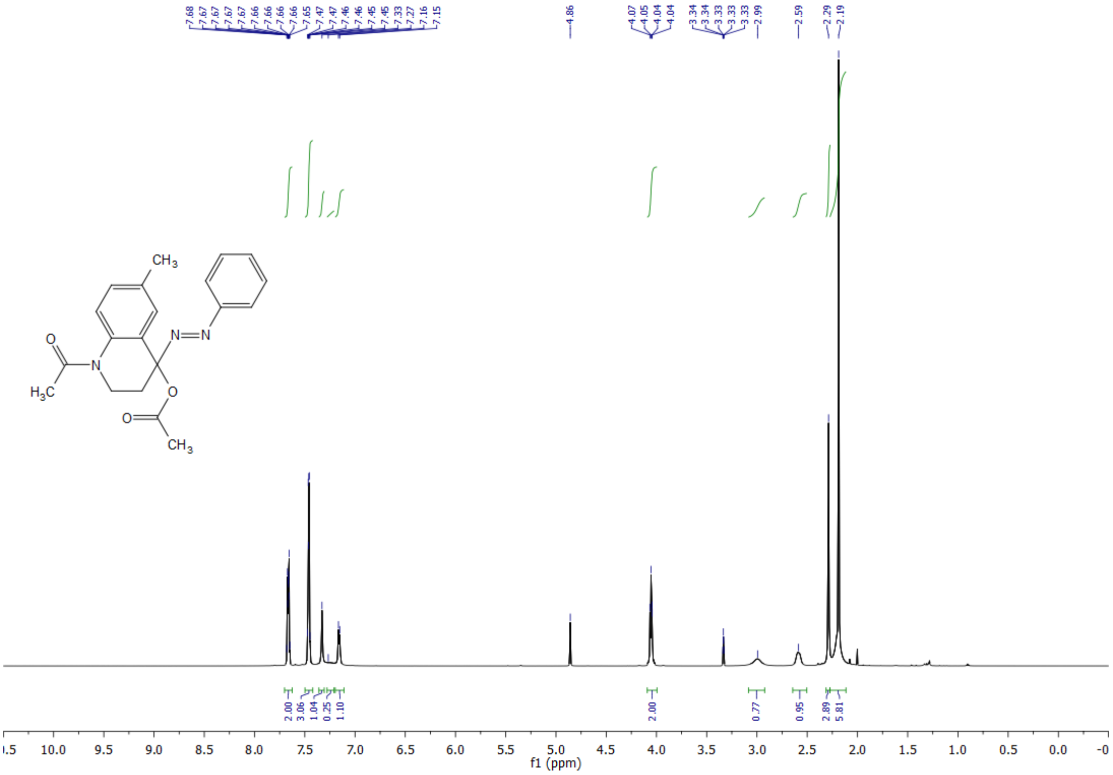

^{13}C NMR spectrum of **8c**

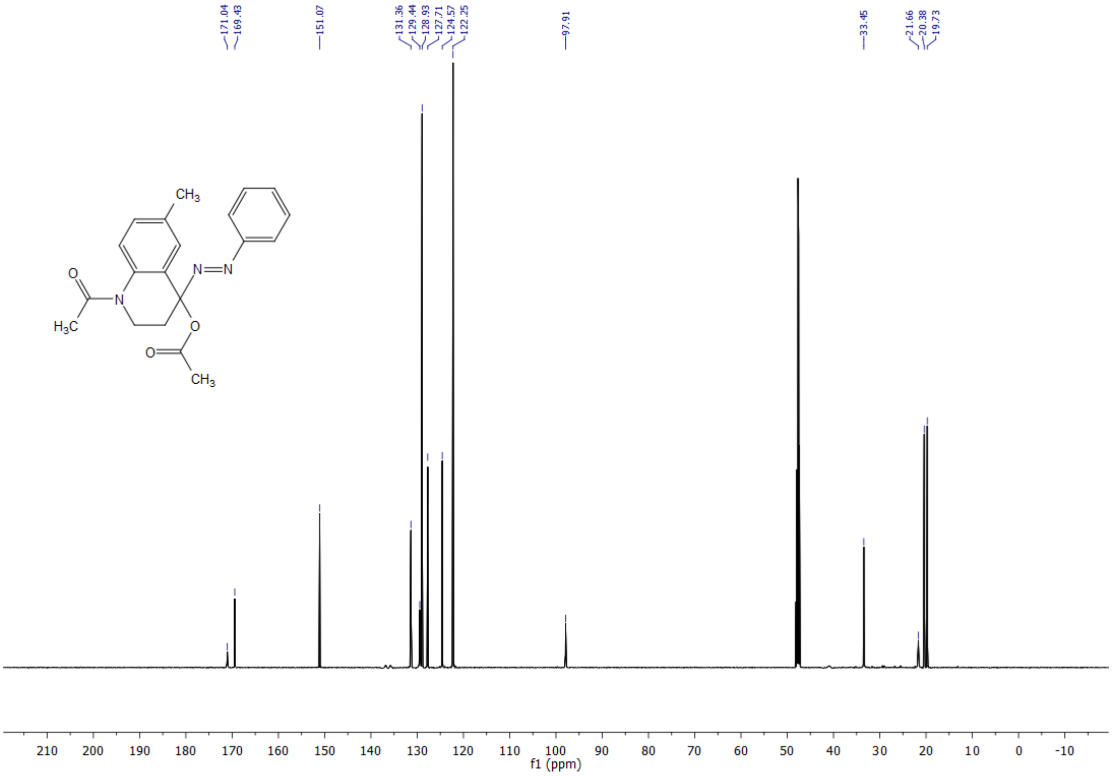

^{1}H NMR spectrum of **8d**

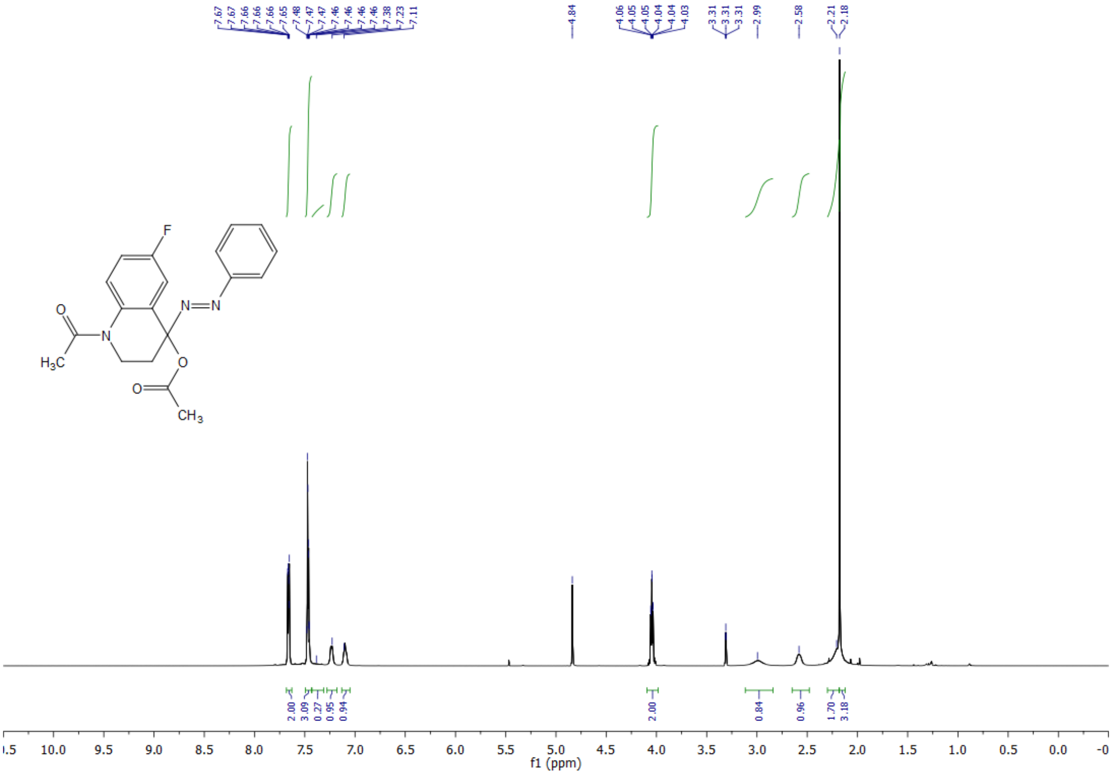

^{13}C NMR spectrum of **8d**

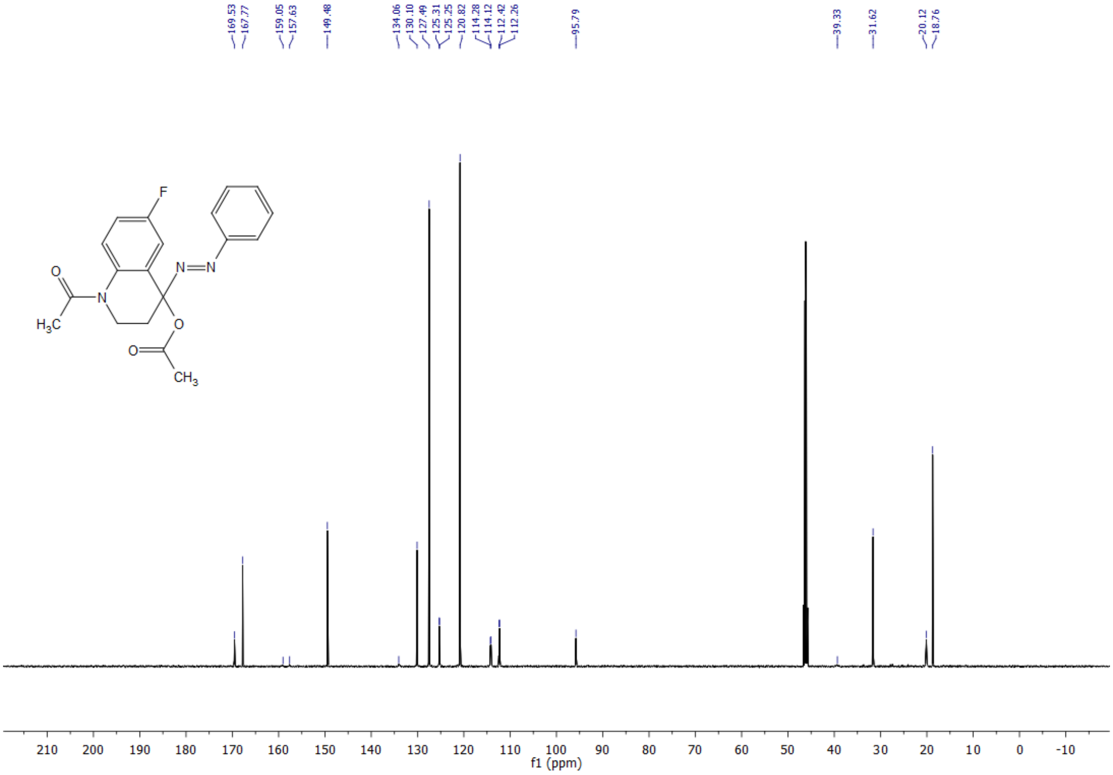

^{1}H NMR spectrum of **8e**

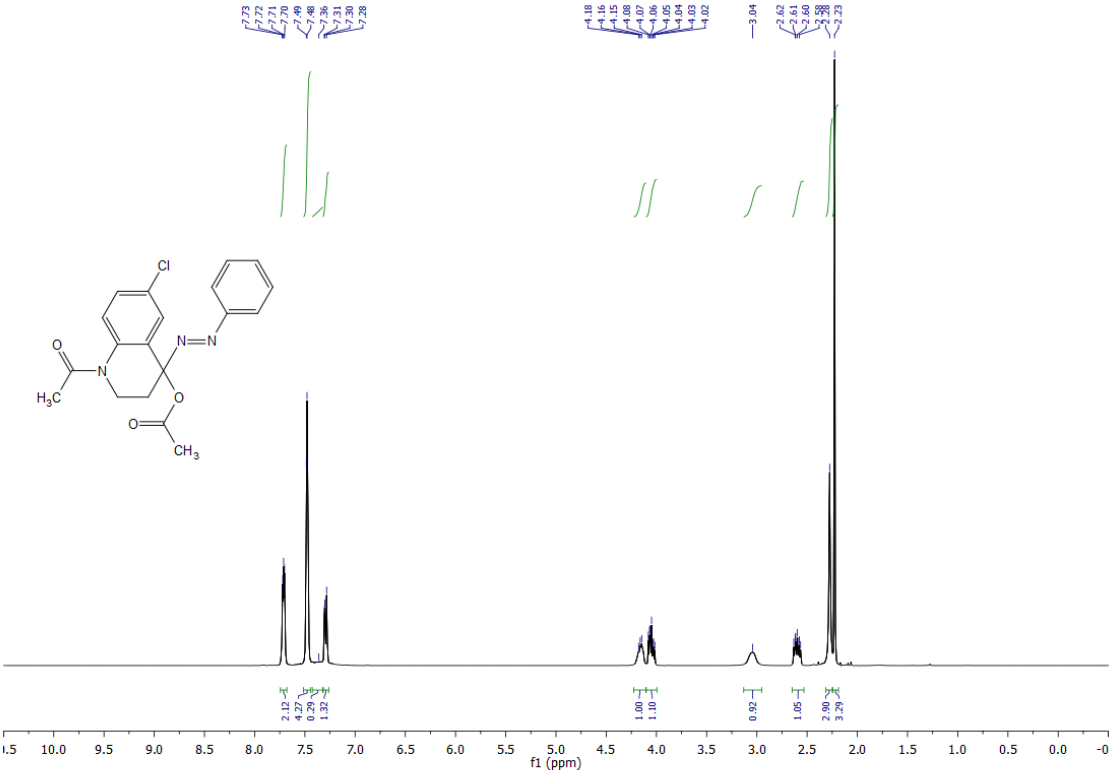

^{13}C NMR spectrum of **8e**

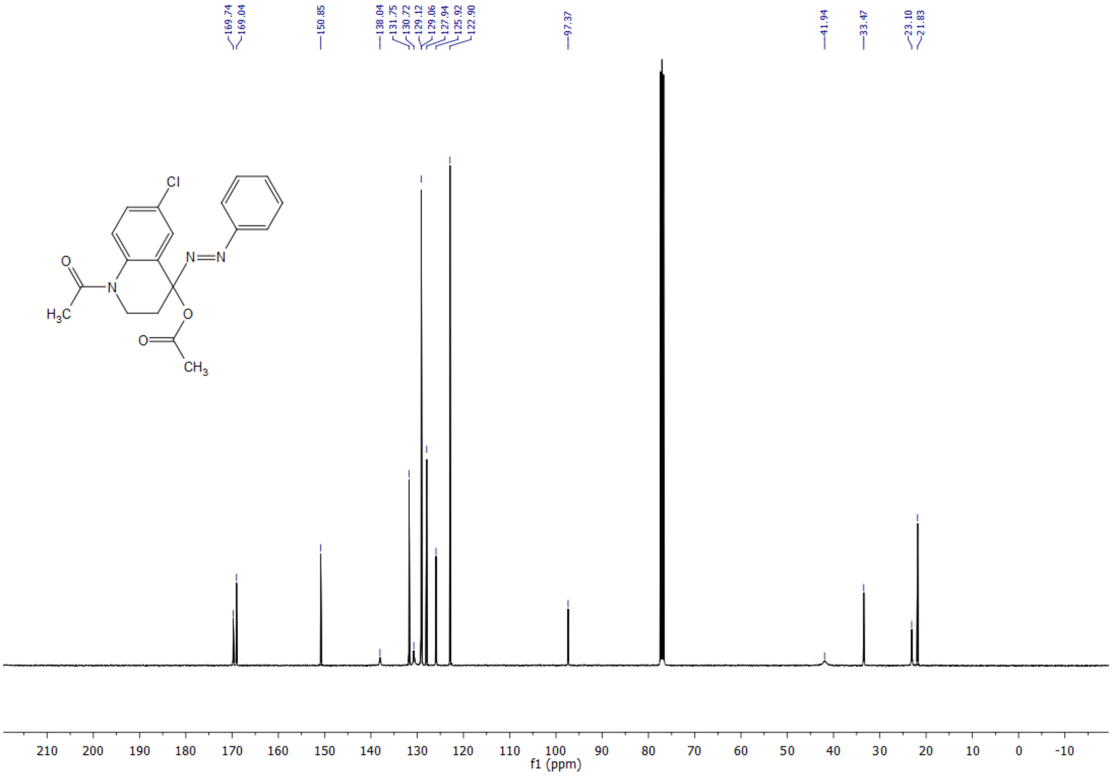

^{1}H NMR spectrum of **8f**

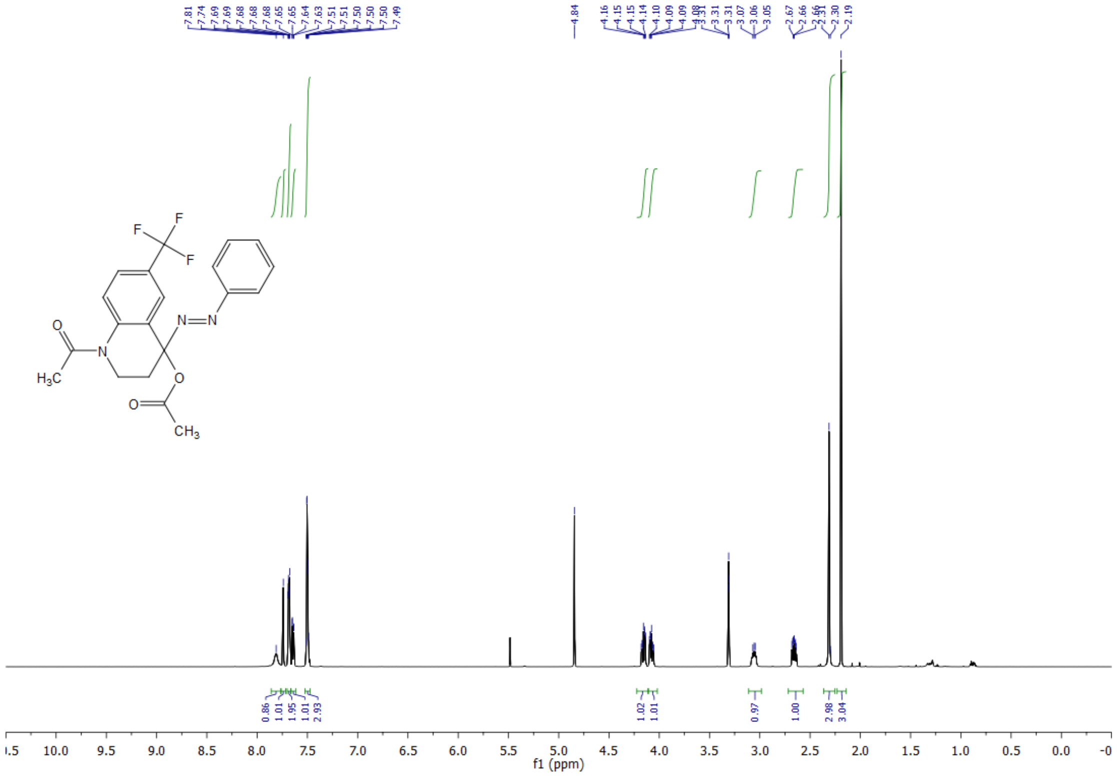

^{13}C NMR spectrum of **8f**

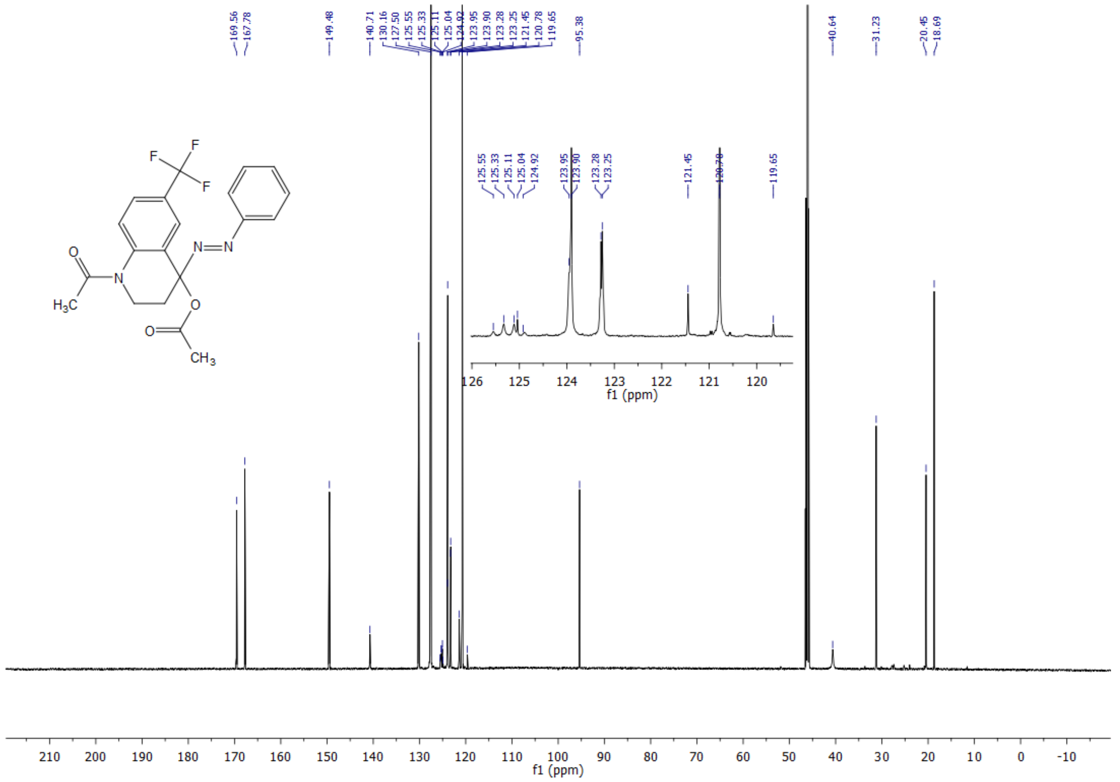

^{1}H NMR spectrum of **8g**

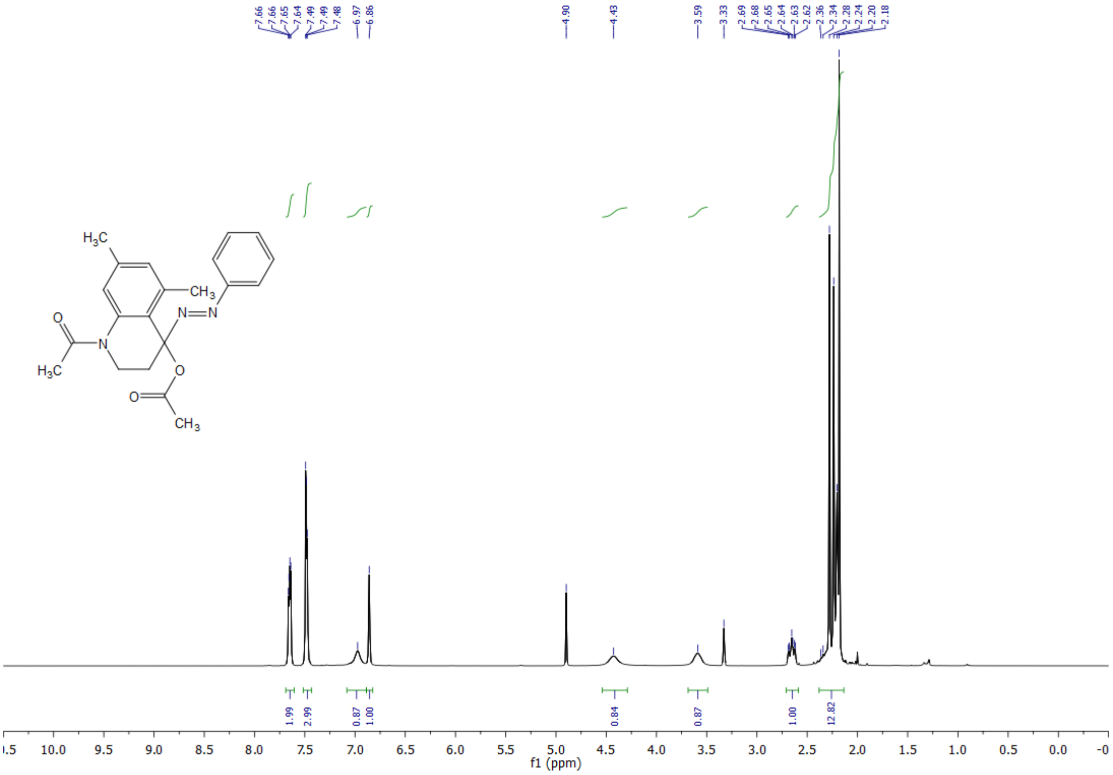

^{13}C NMR spectrum **of 8g**

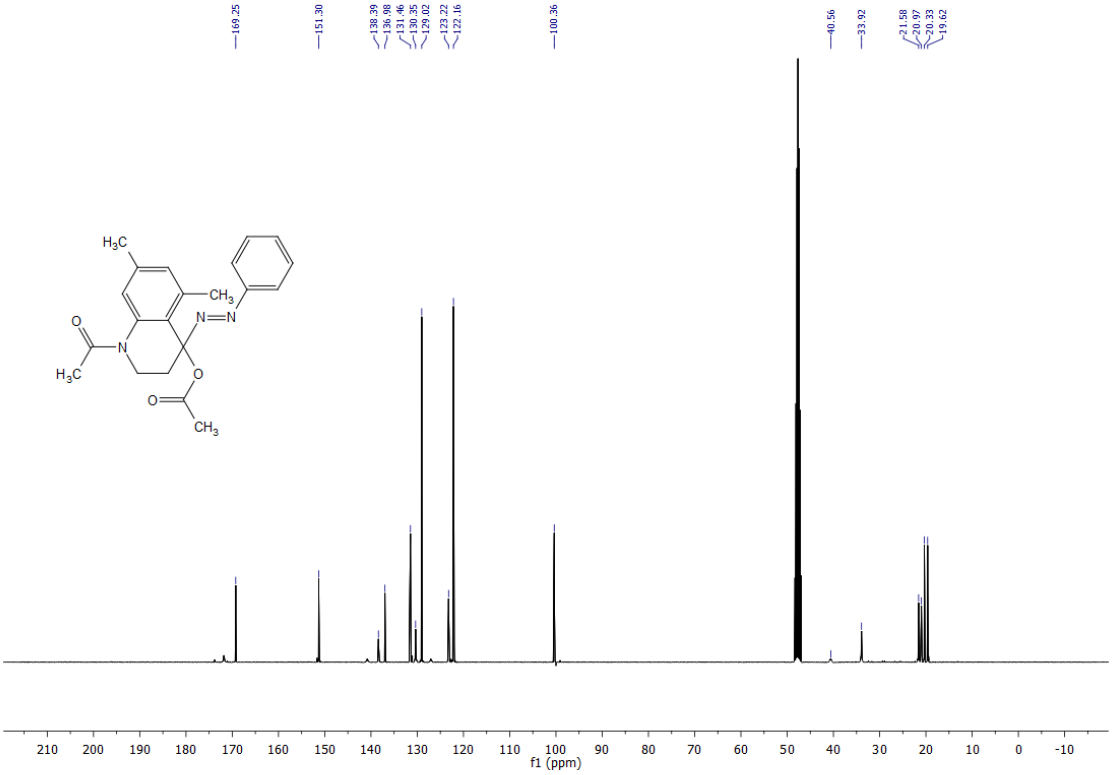

^{1}H NMR spectrum of **10a**

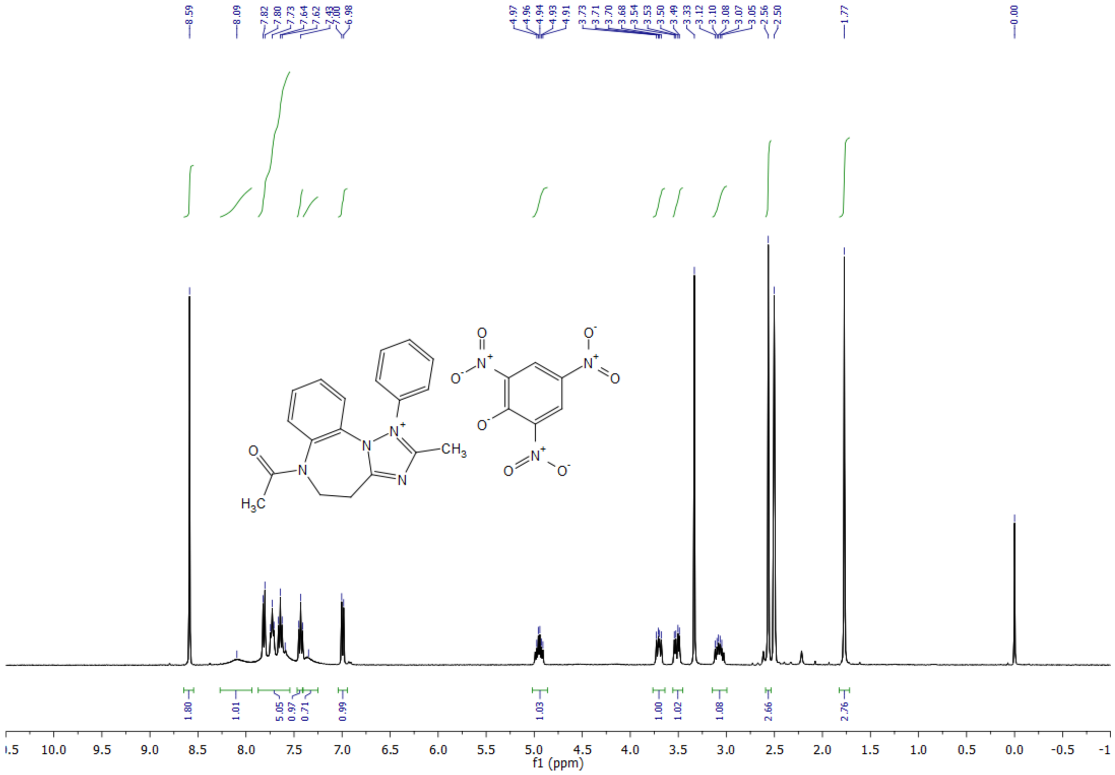

^{13}C NMR spectrum of **10a**

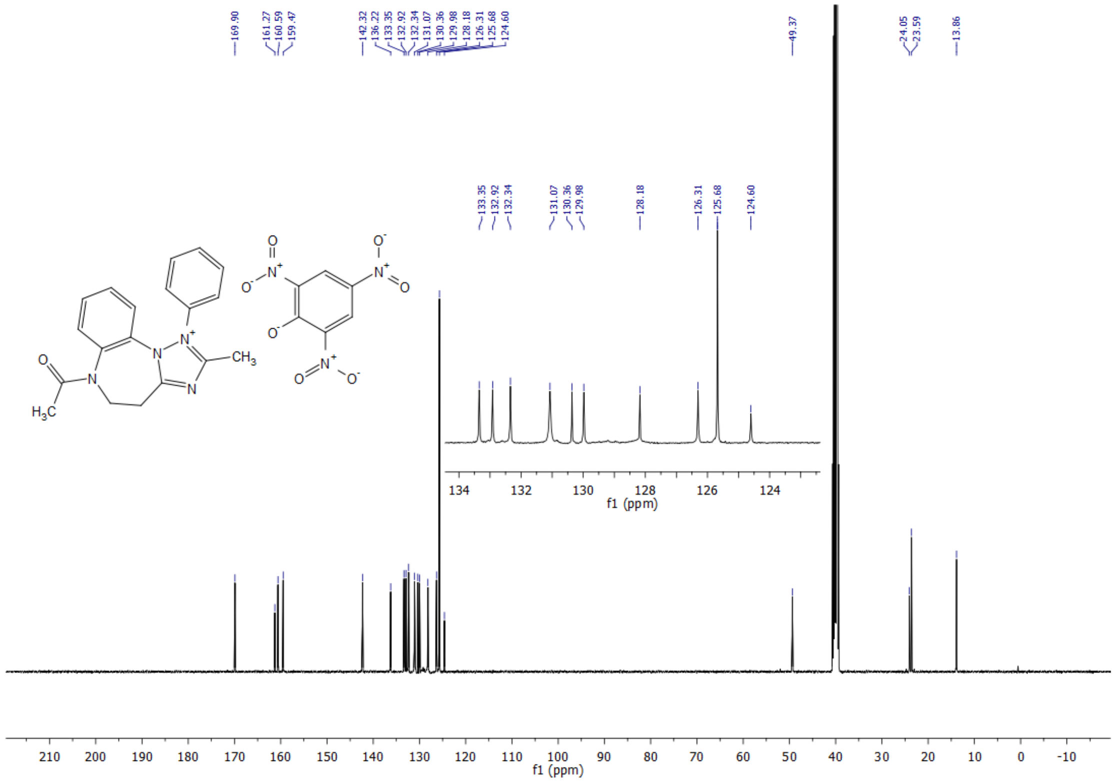

^{1}H NMR spectrum of **10b**

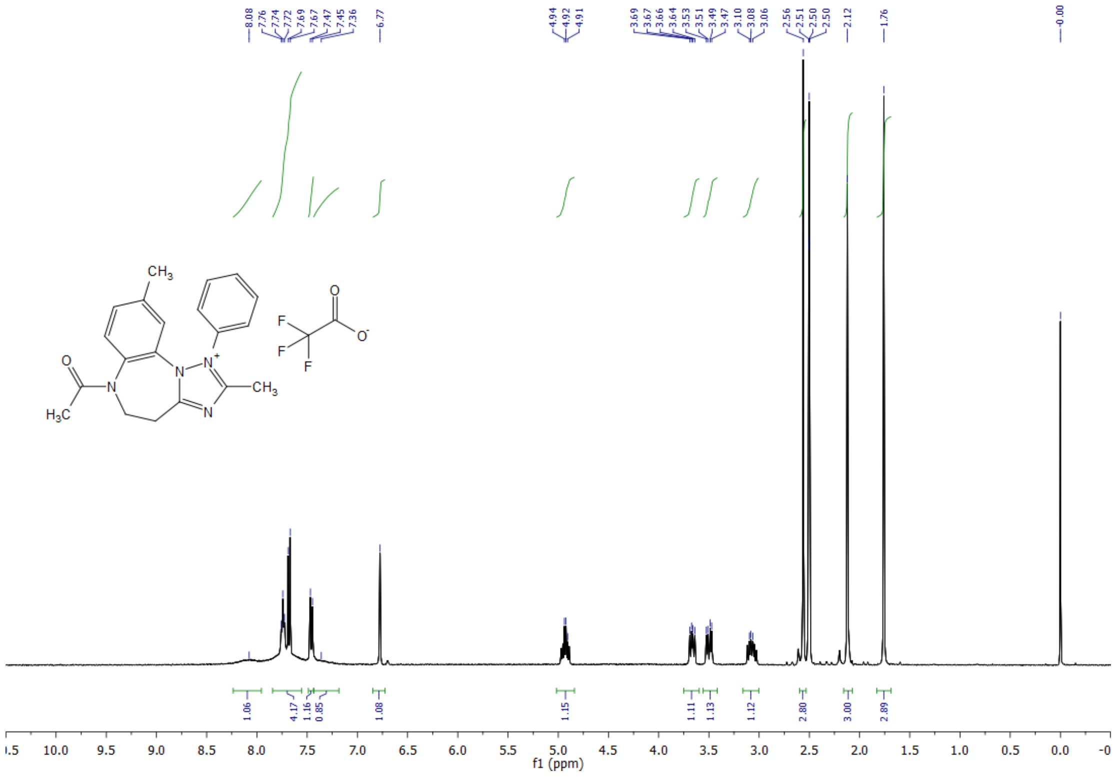

^{13}C NMR spectrum of **10b**

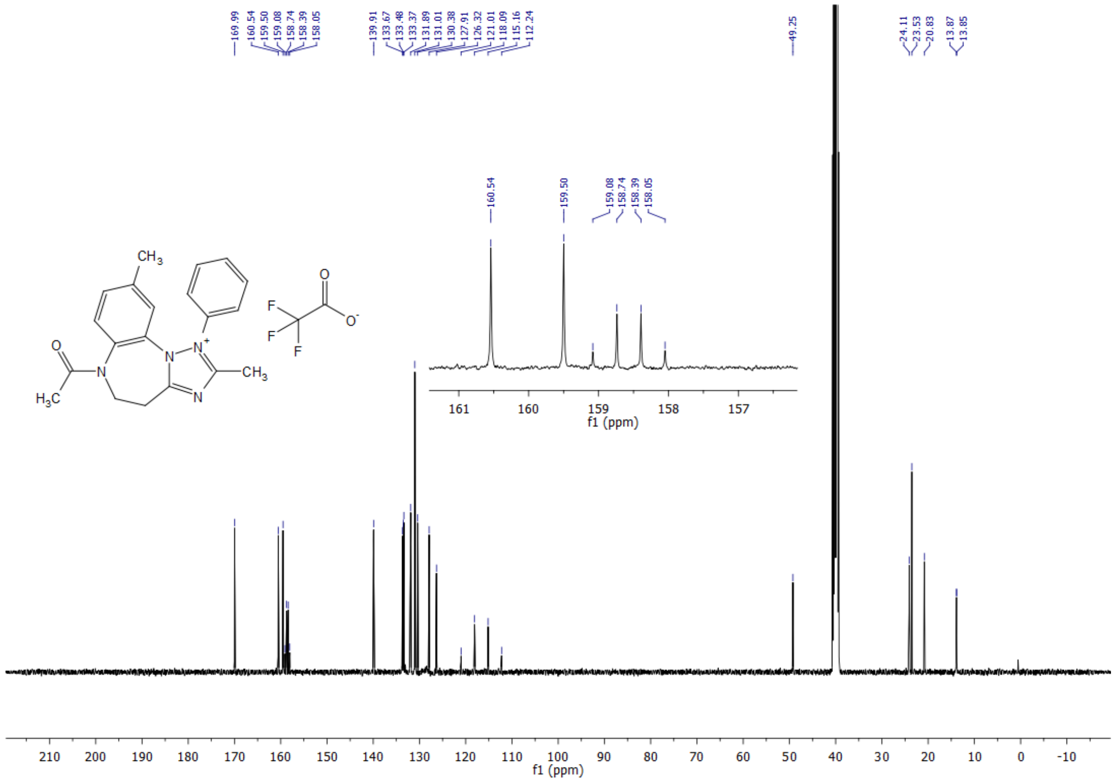

^{1}H NMR spectrum of **10c**

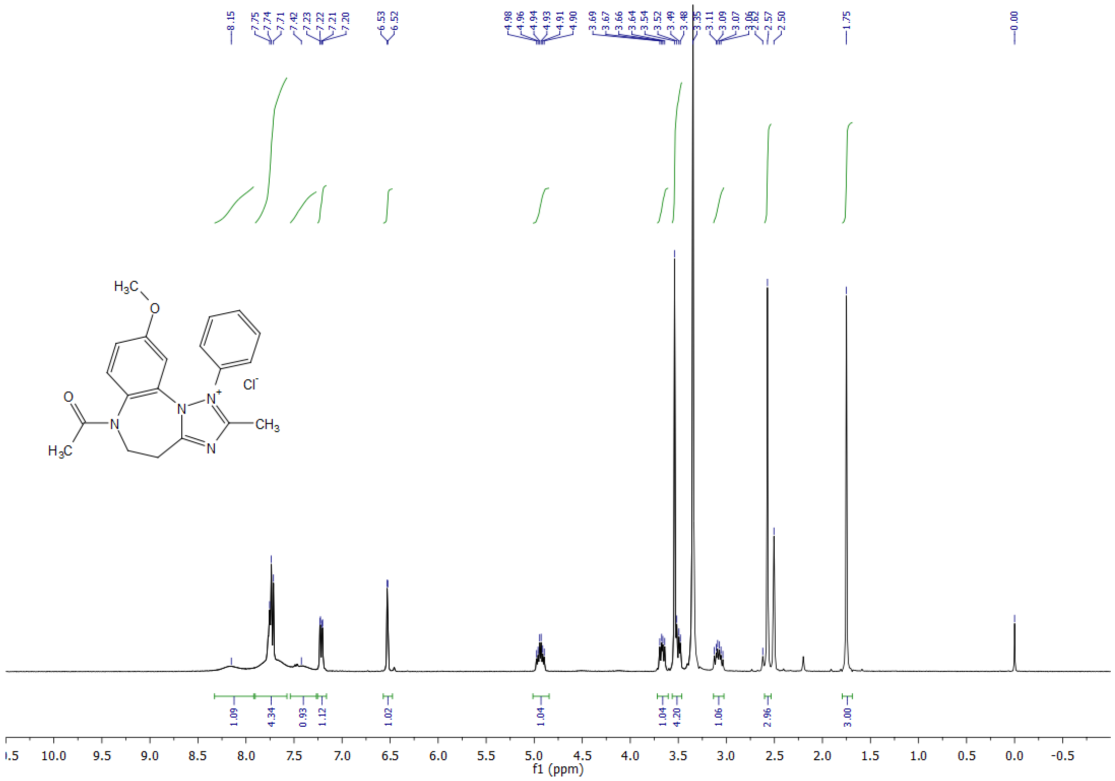

^{13}C NMR spectrum of **10c**

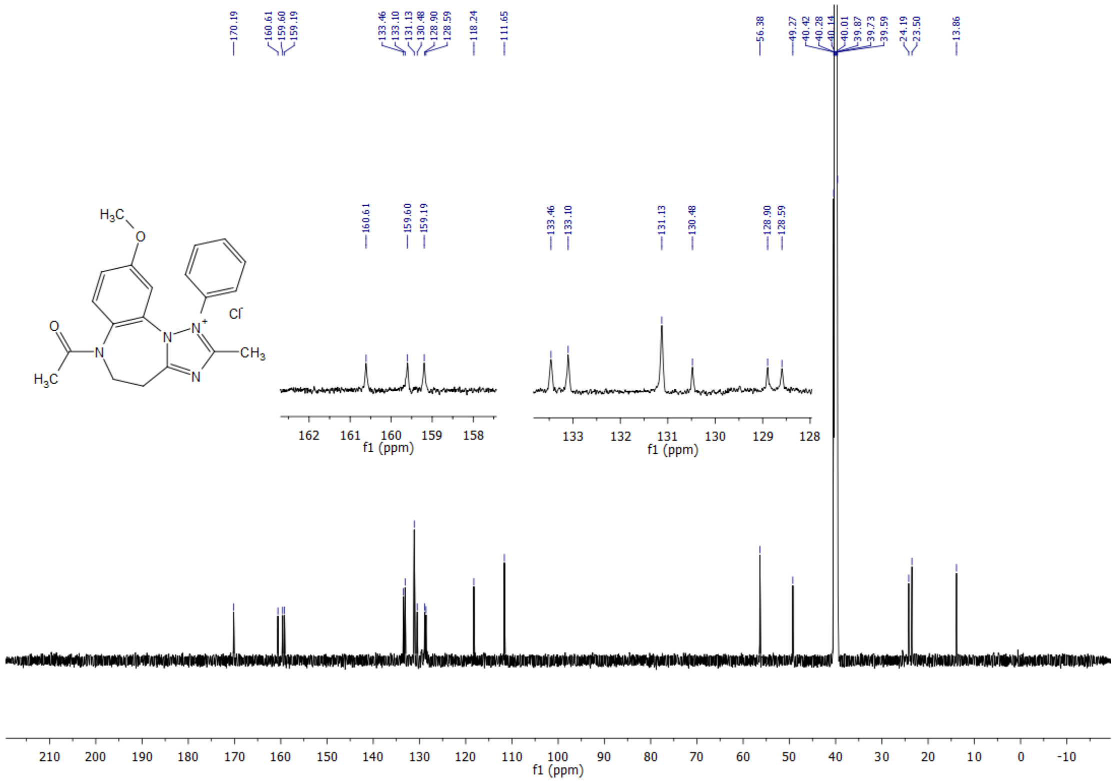

^{1}H NMR spectrum of **10d**

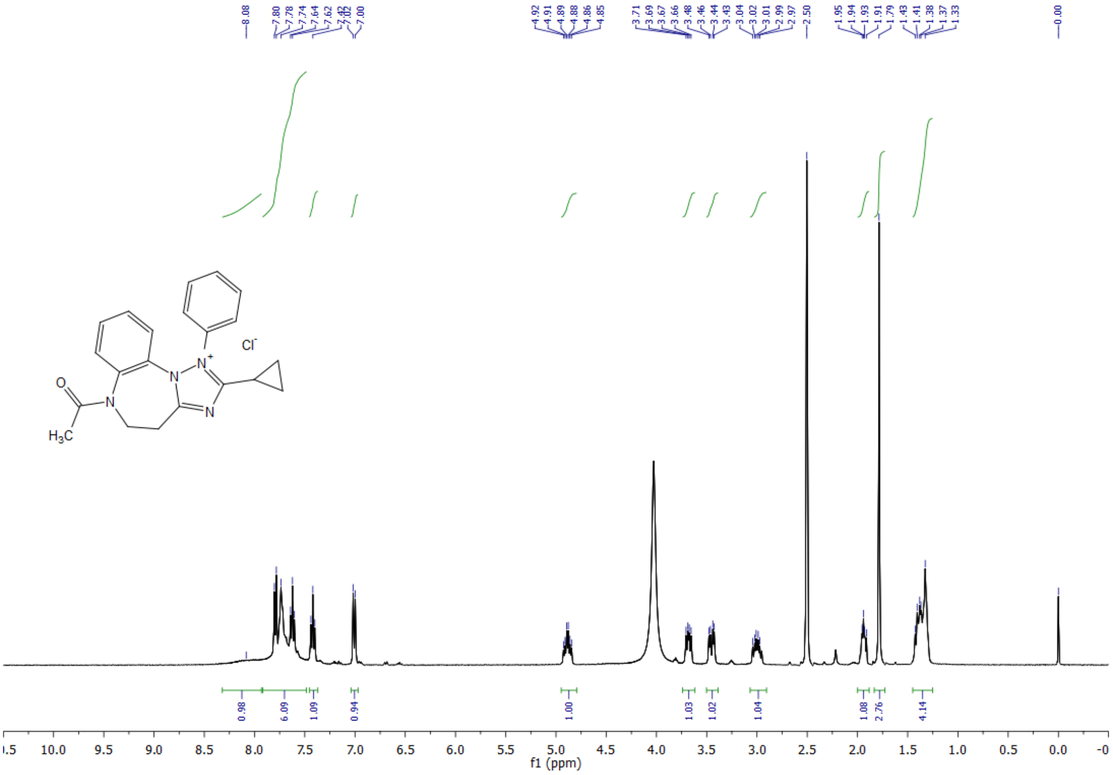

^{13}C NMR spectrum of **10d**

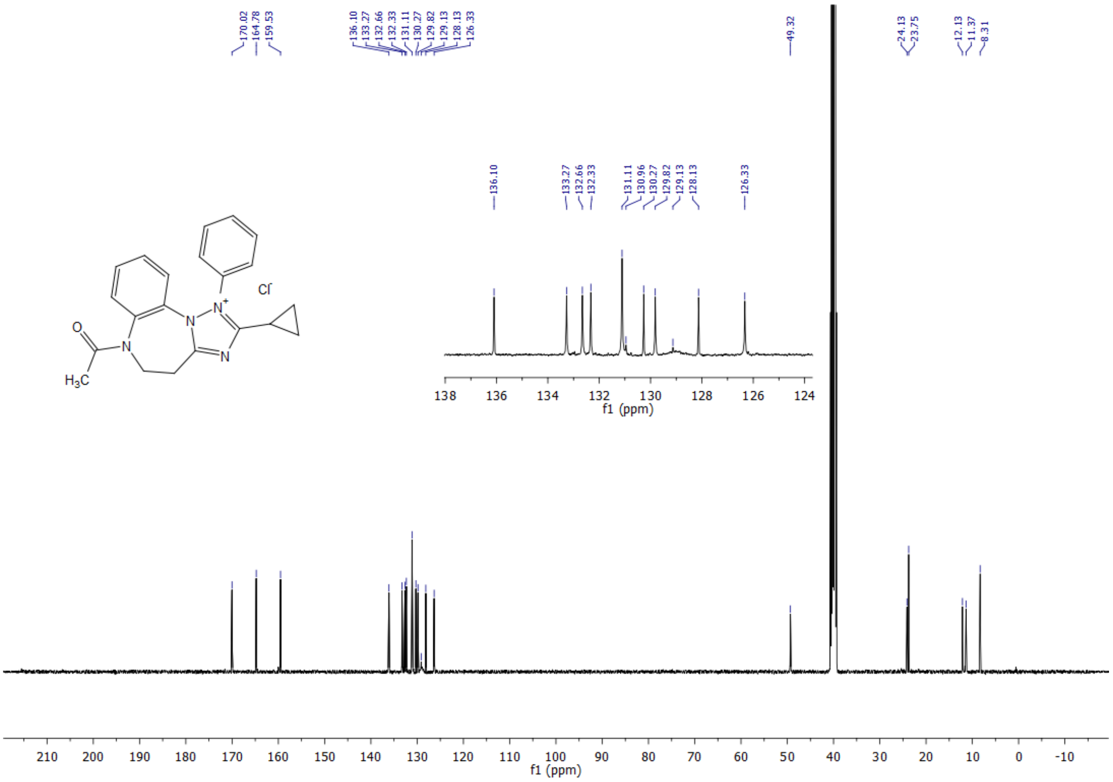

^{1}H NMR spectrum of **10e**

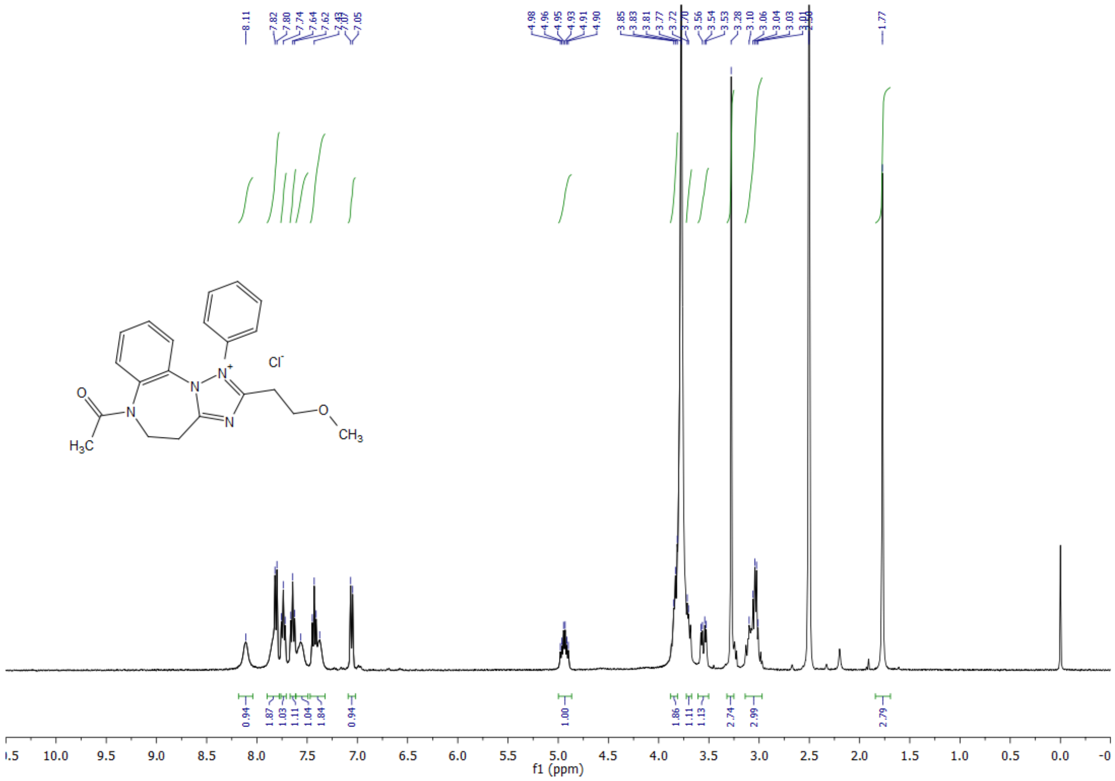

^{13}C NMR spectrum of **10e**

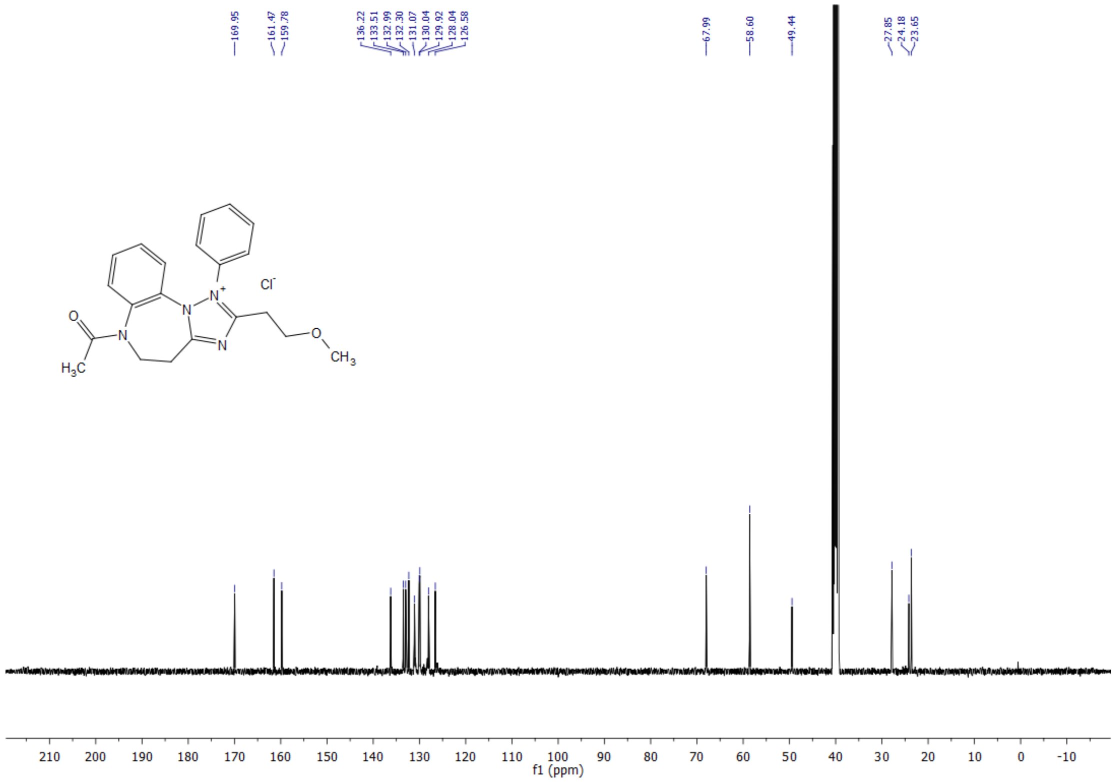

^{1}H NMR spectrum of **10f**

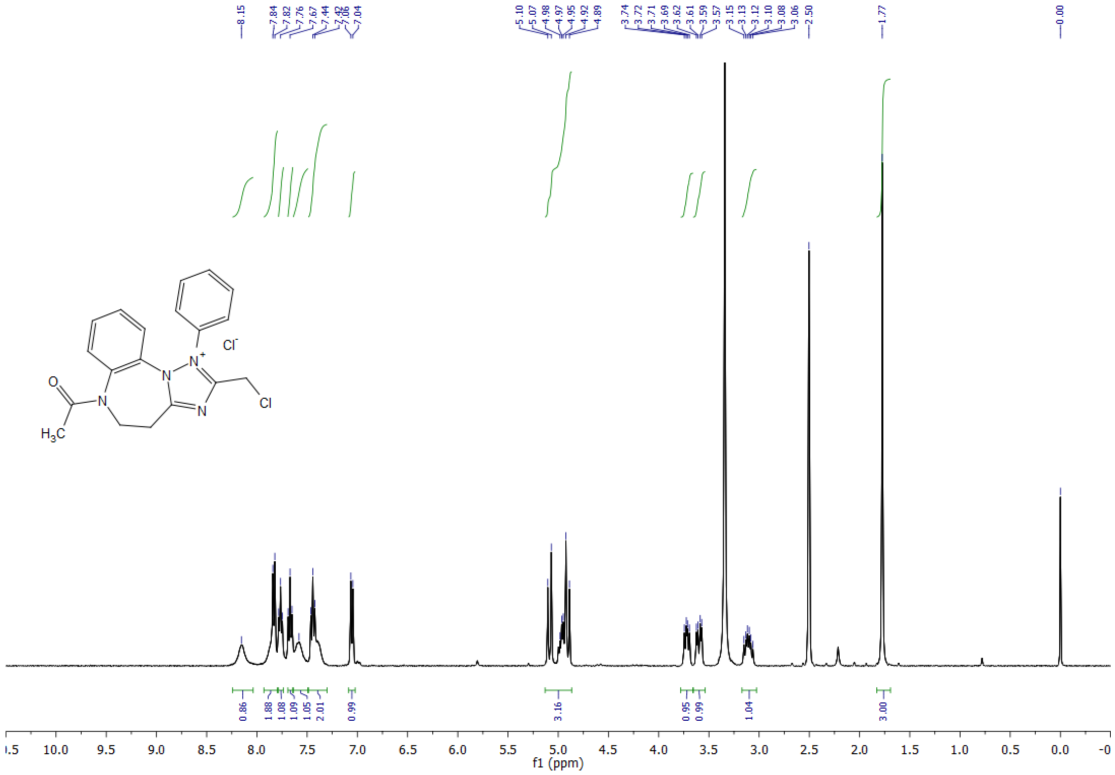

^{13}C NMR spectrum of **10f**

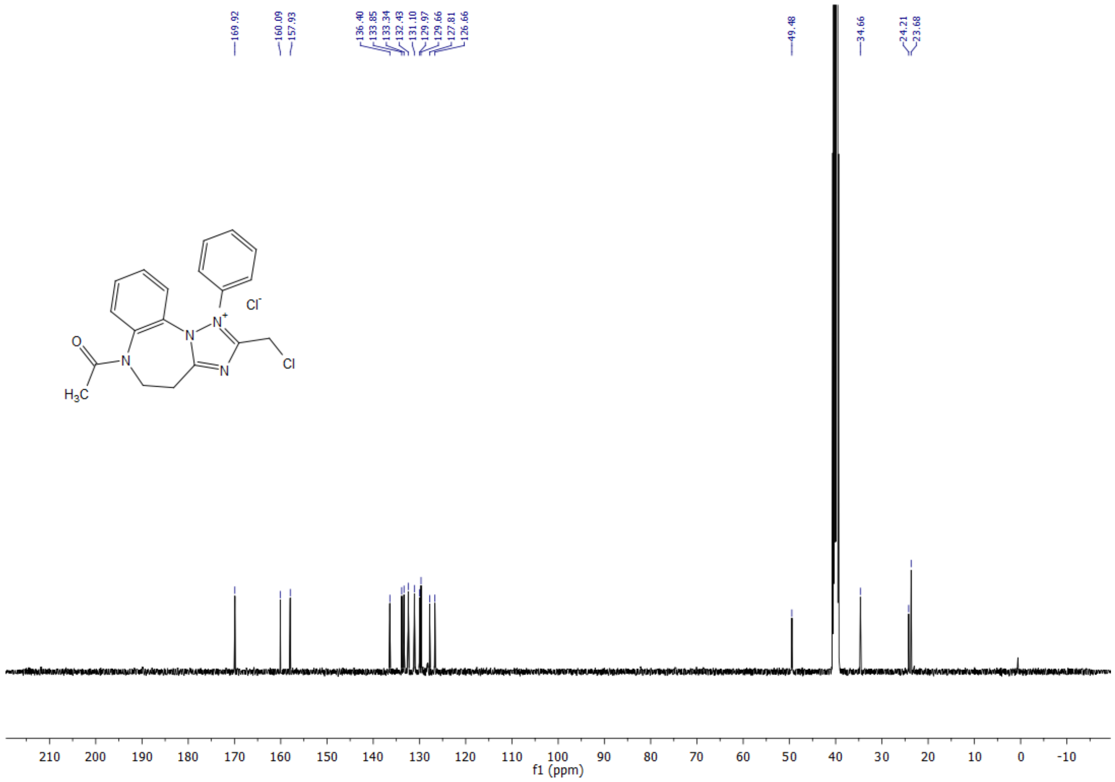

^{1}H NMR spectrum of **10g**

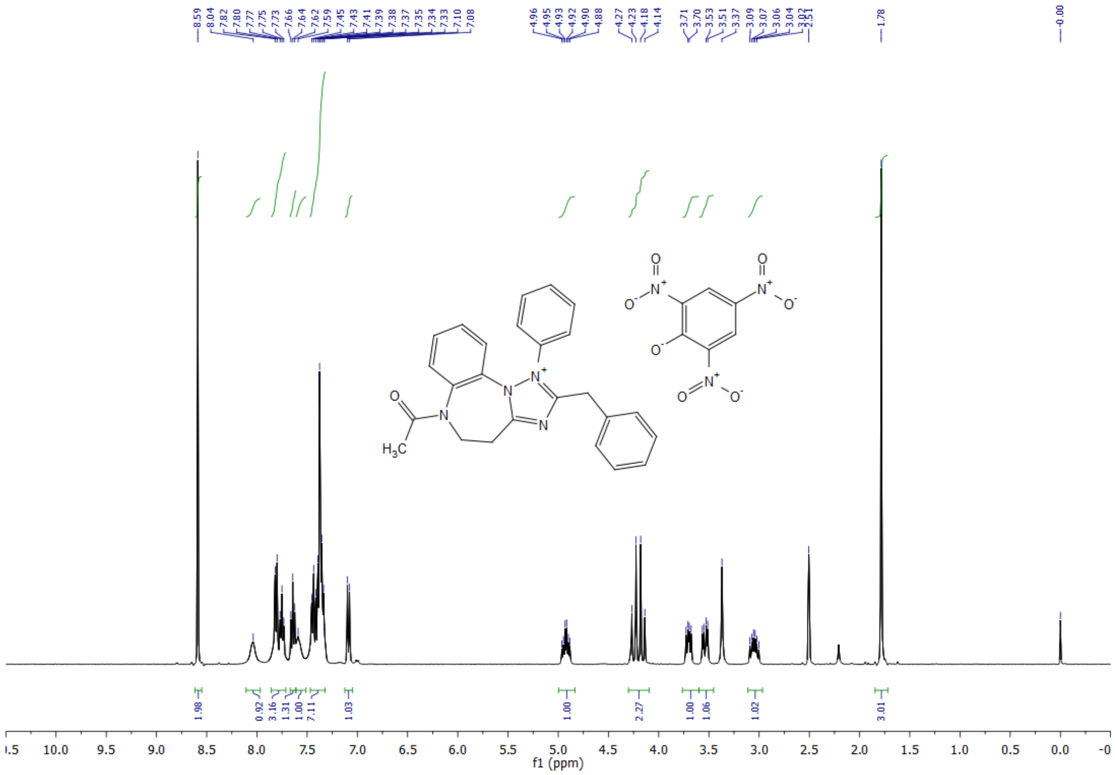

^{13}C NMR spectrum of **10g**

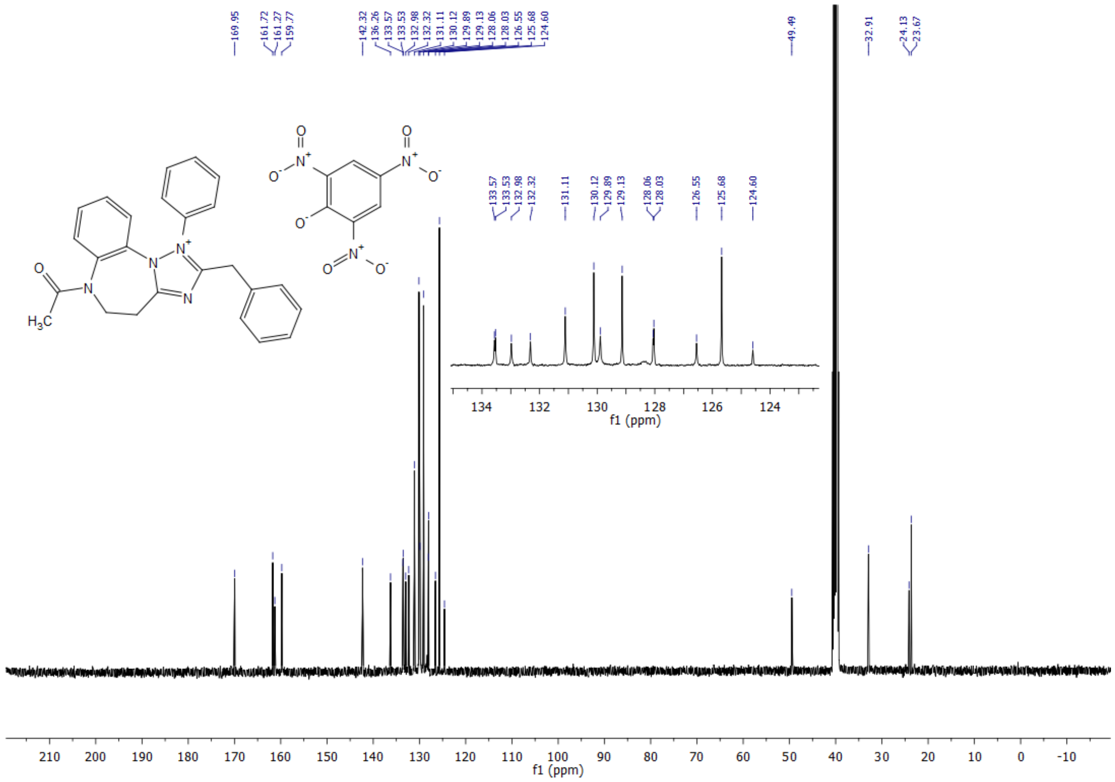

^{1}H NMR spectrum of **10h**

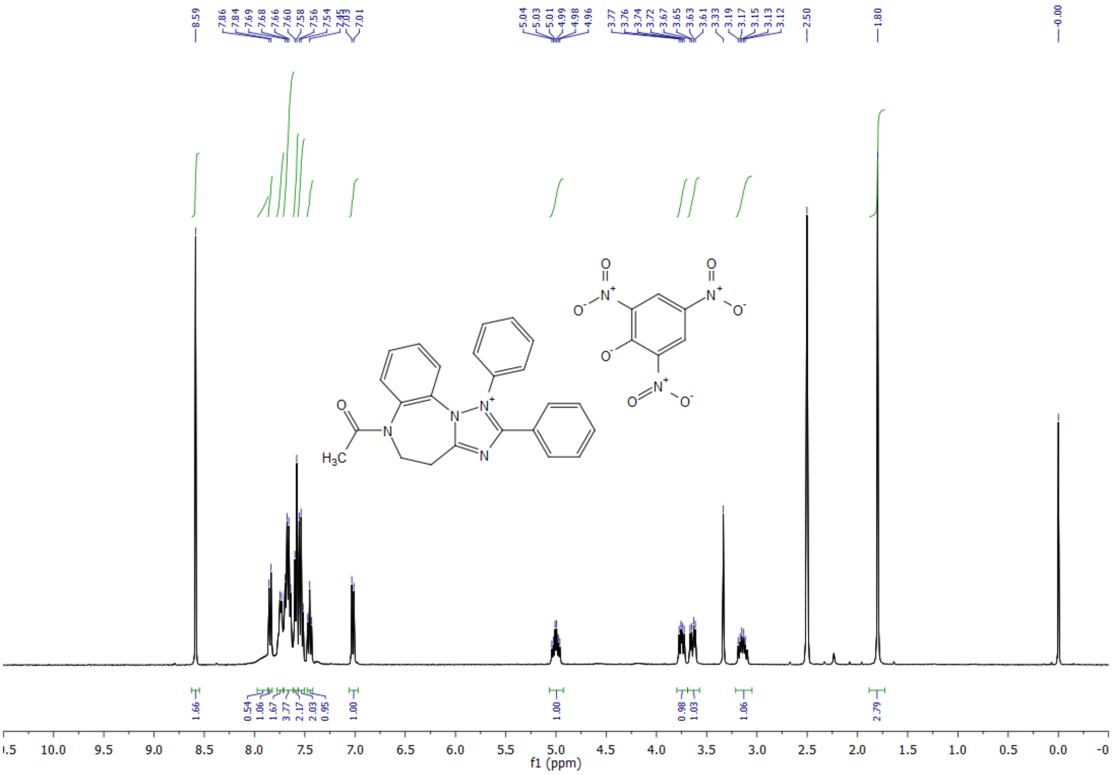

^{13}C NMR spectrum of **10h**

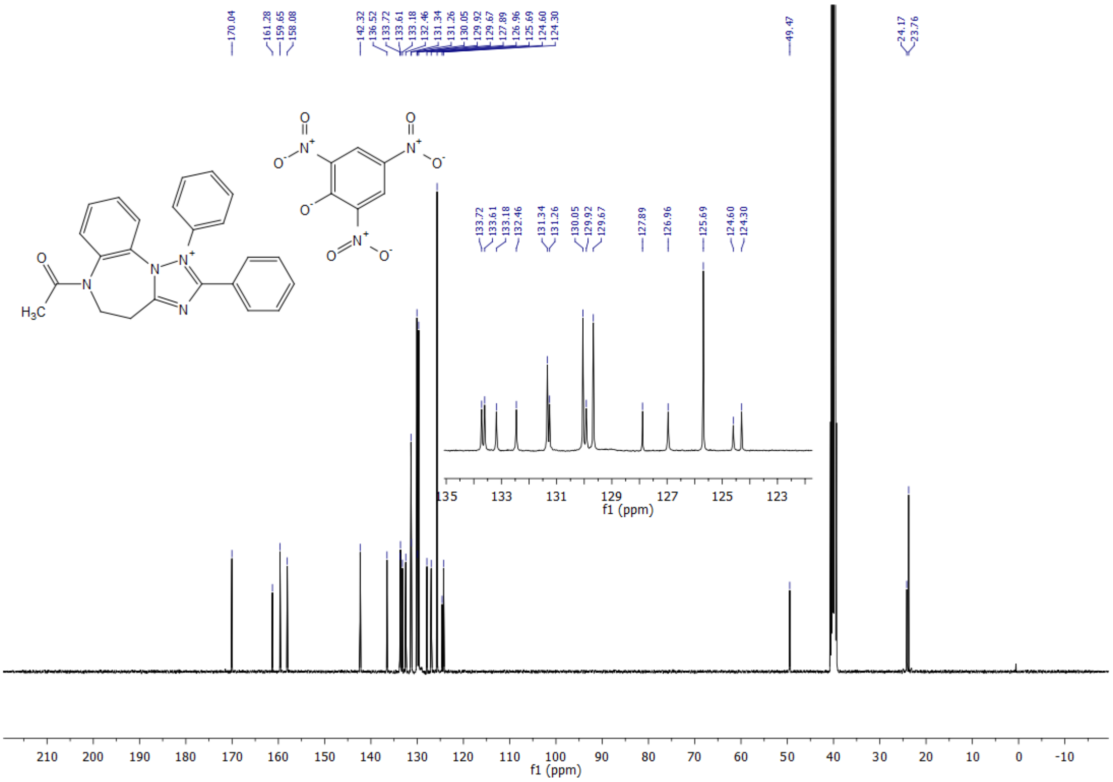

^{1}H NMR spectrum of **10i**

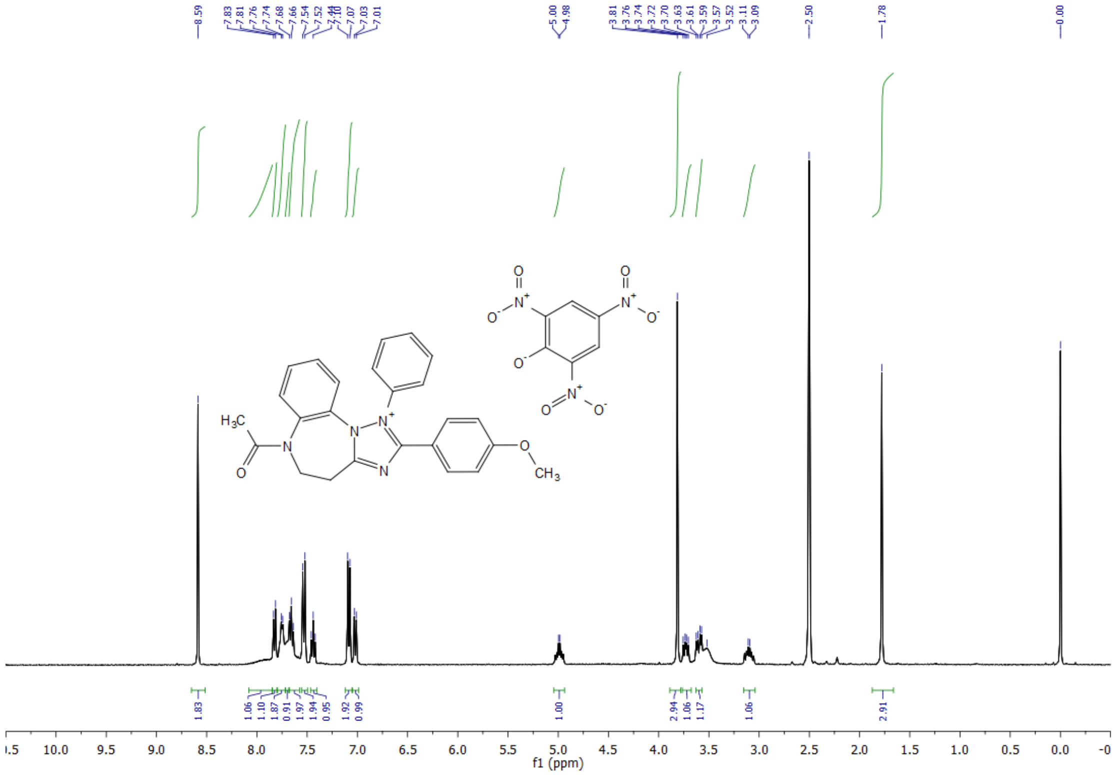

^{13}C NMR spectrum of **10i**

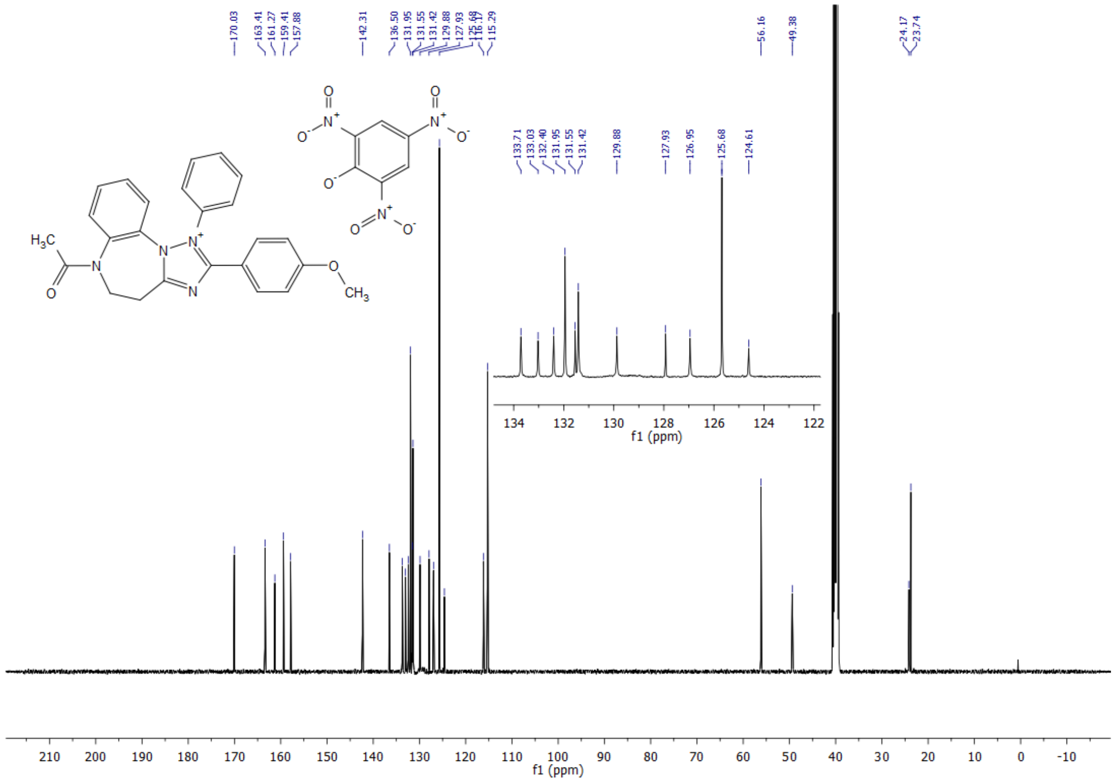

^{1}H NMR spectrum of **10j**

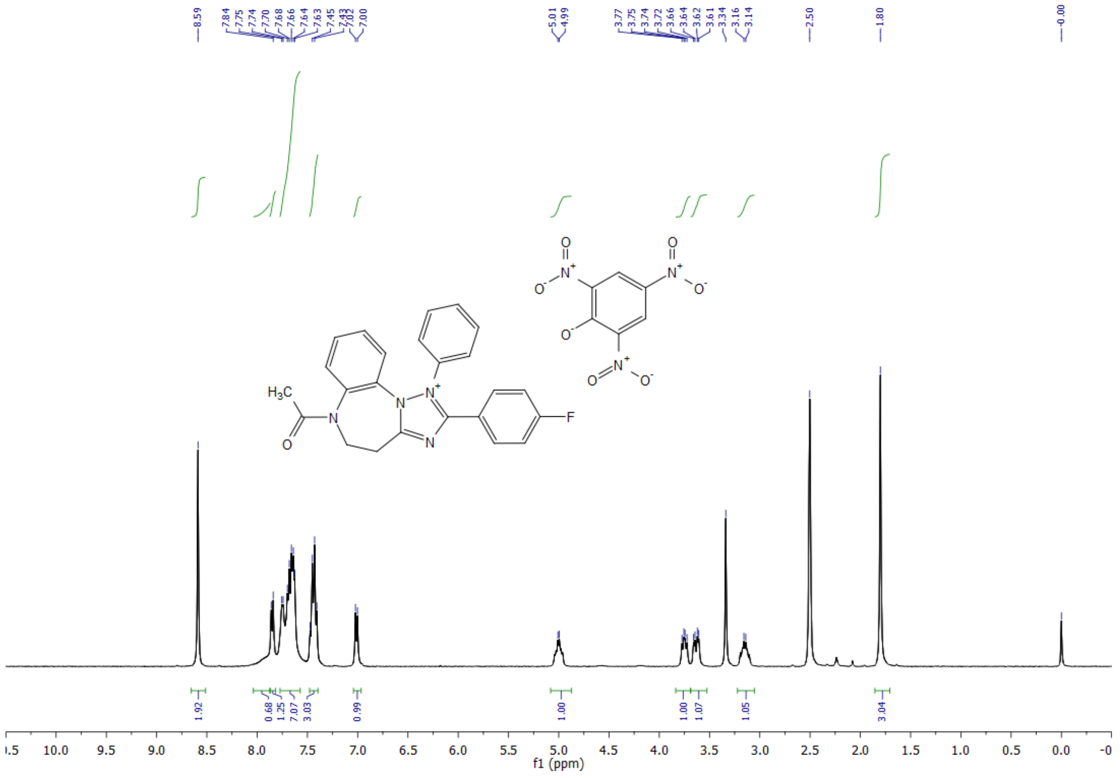

^{13}C NMR spectrum of **10j**

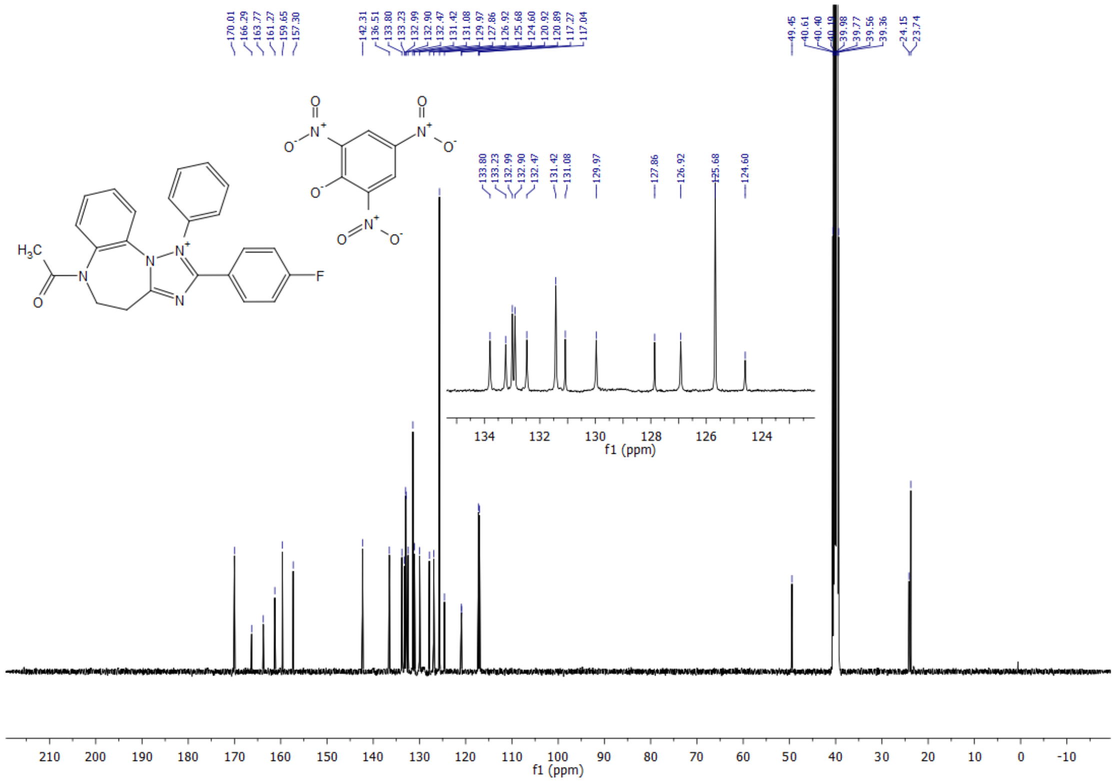

^{1}H NMR spectrum of **10k**

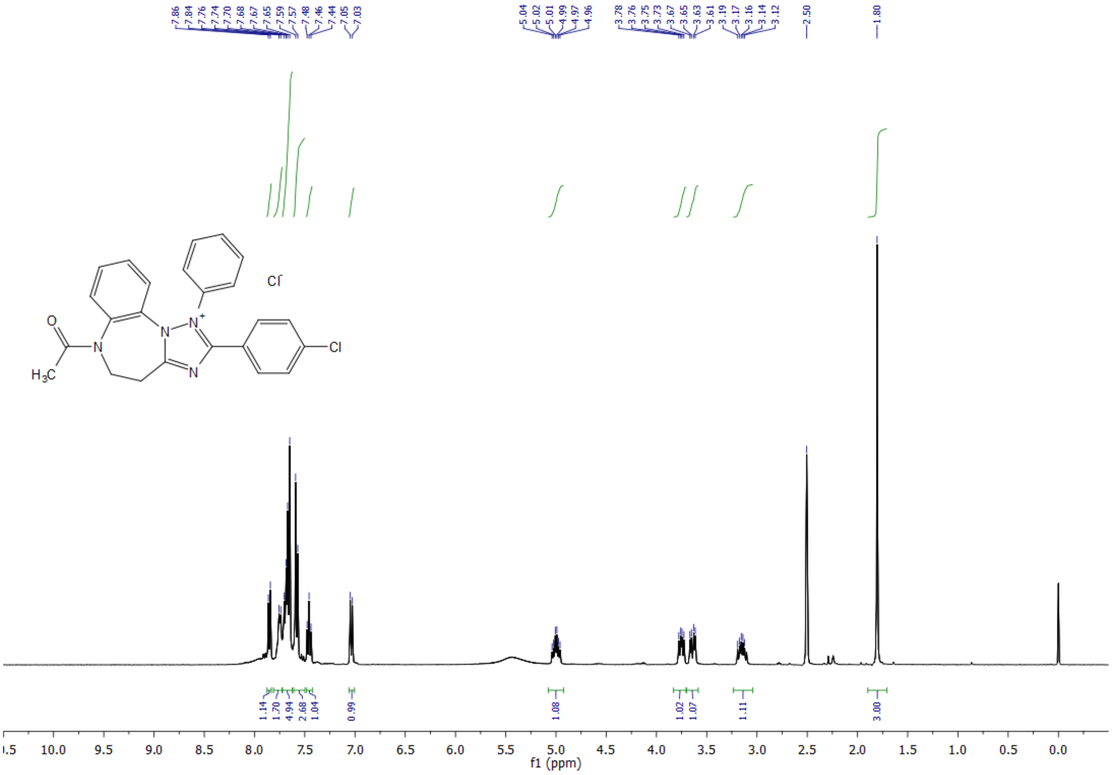

^{13}C NMR spectrum of **10k**

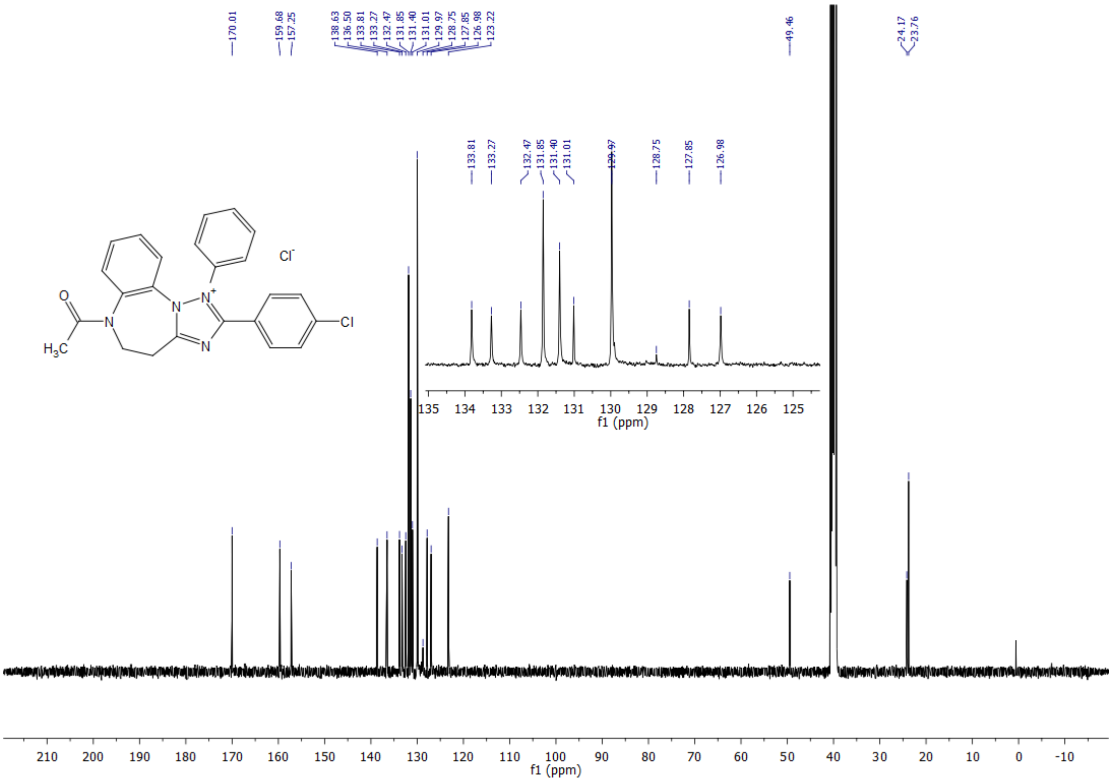

^{1}H NMR spectrum of **10l**

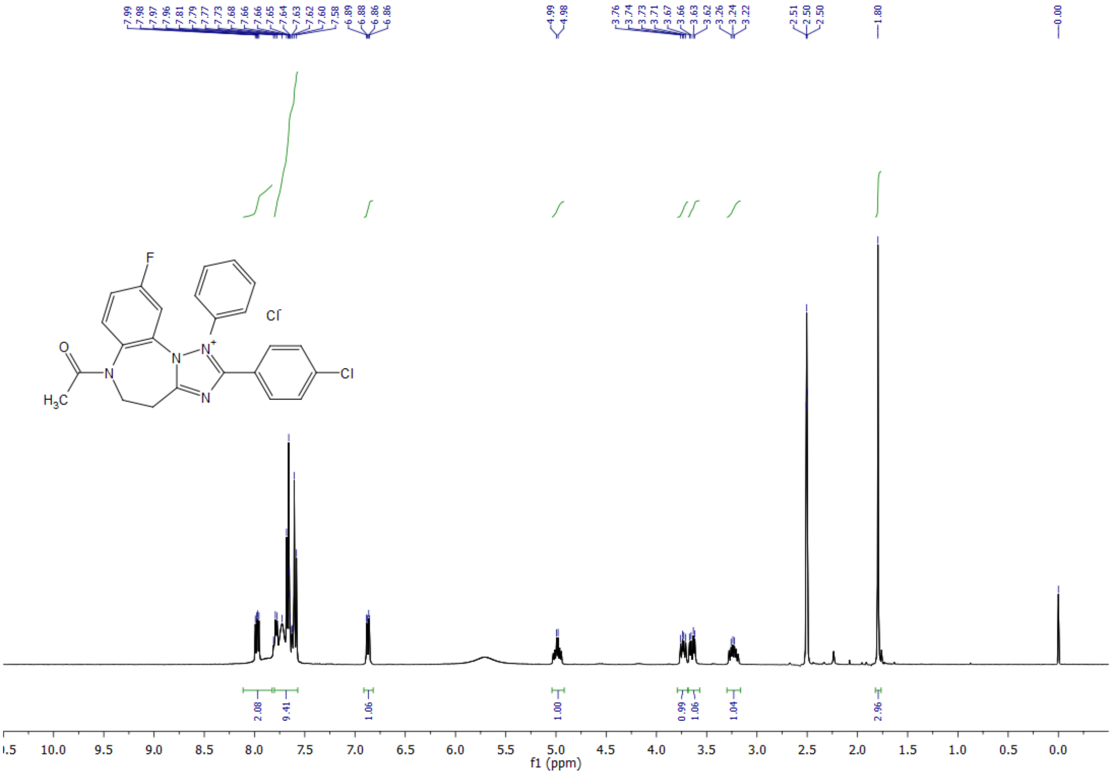

^{13}C NMR spectrum of **10l**

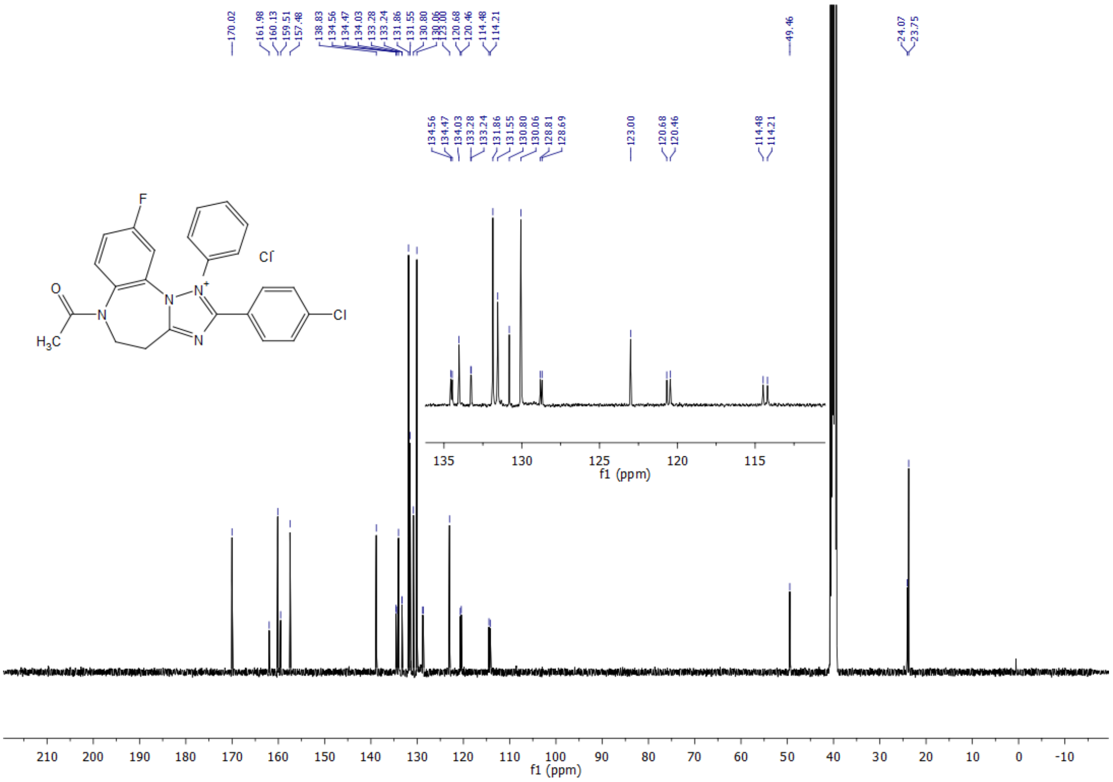

^{1}H NMR spectrum of **10m**

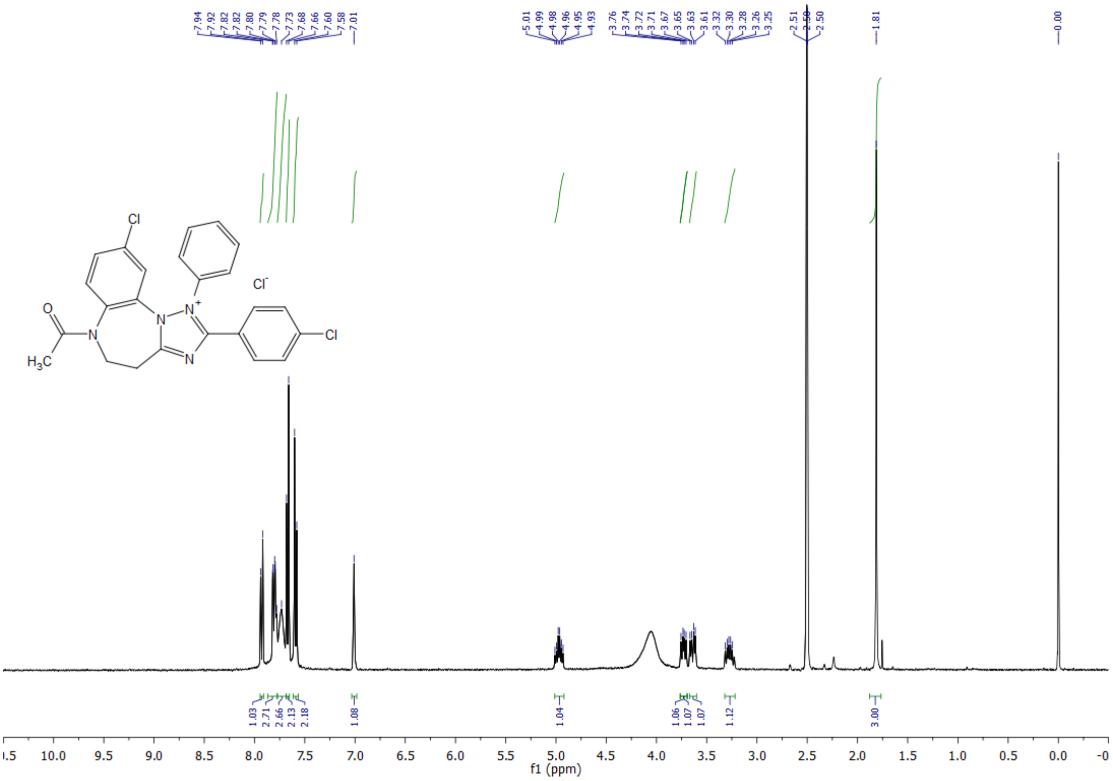

^{13}C NMR spectrum of **10m**

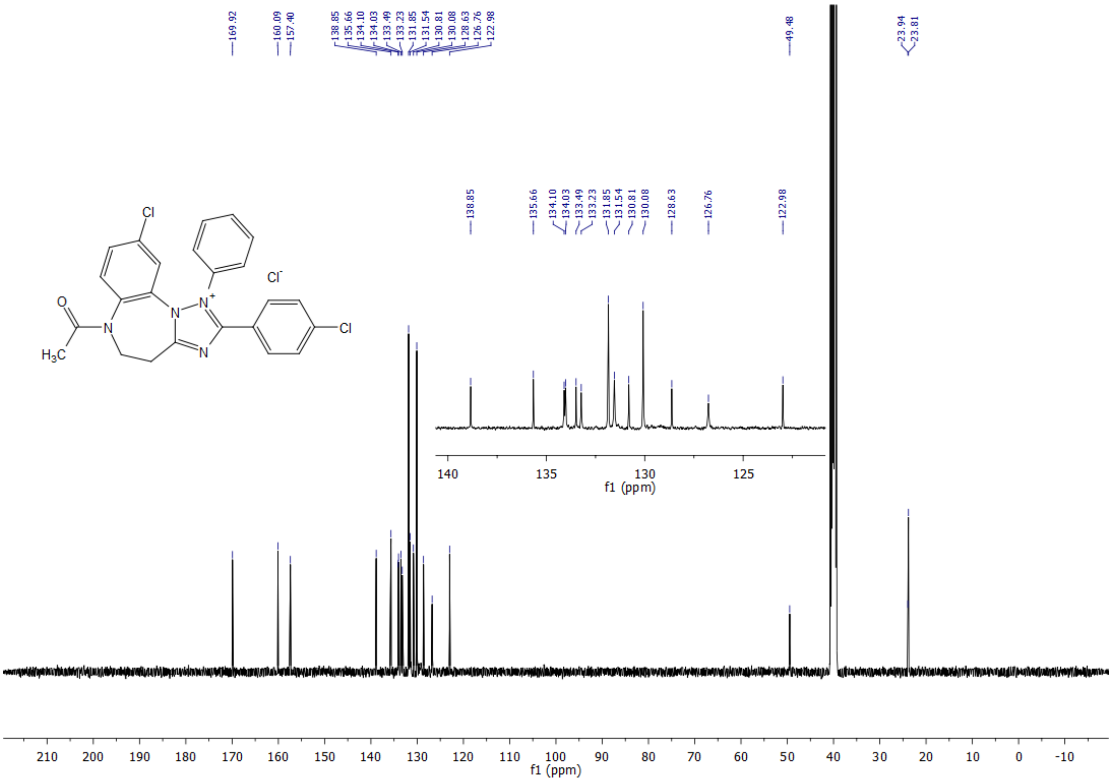

^{1}H NMR spectrum of **10n**

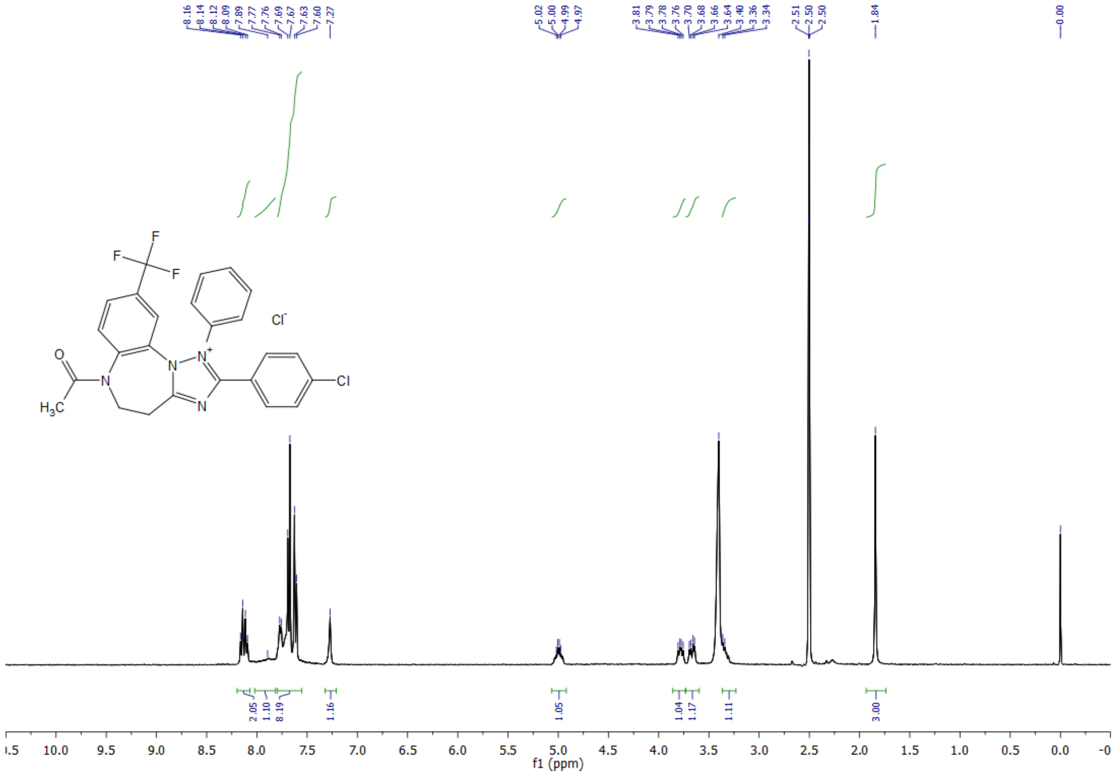

^{13}C NMR spectrum of **10n**

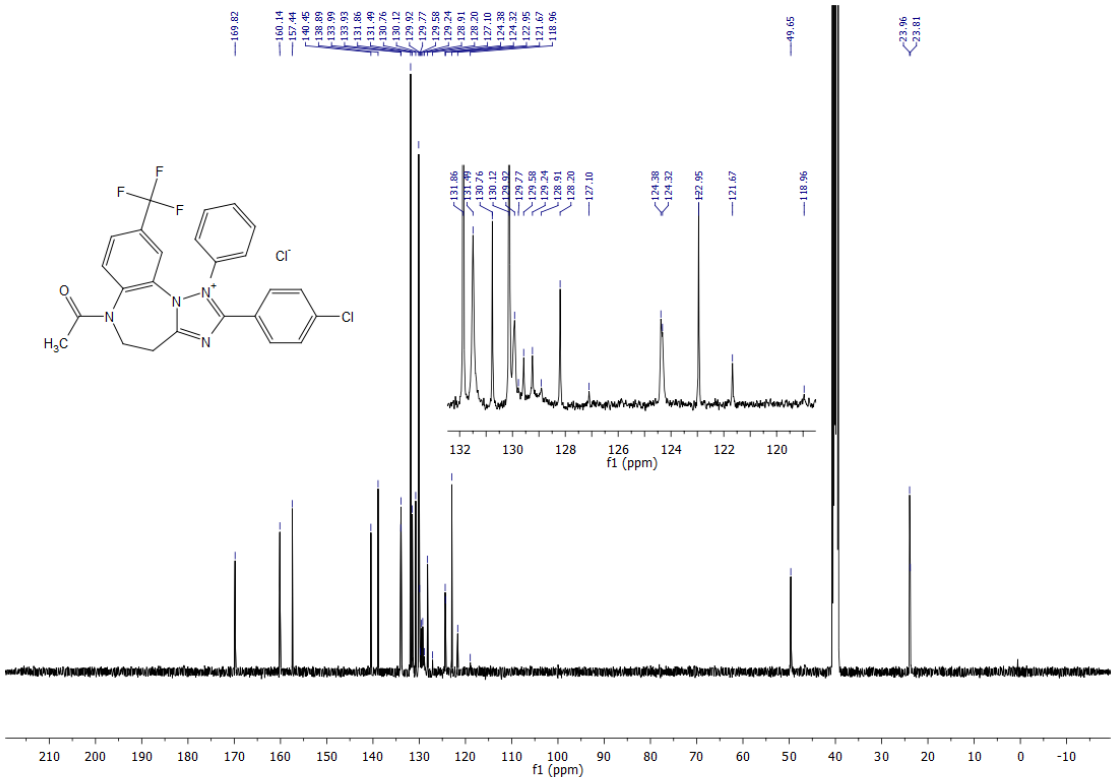

^{1}H NMR spectrum of **10o**

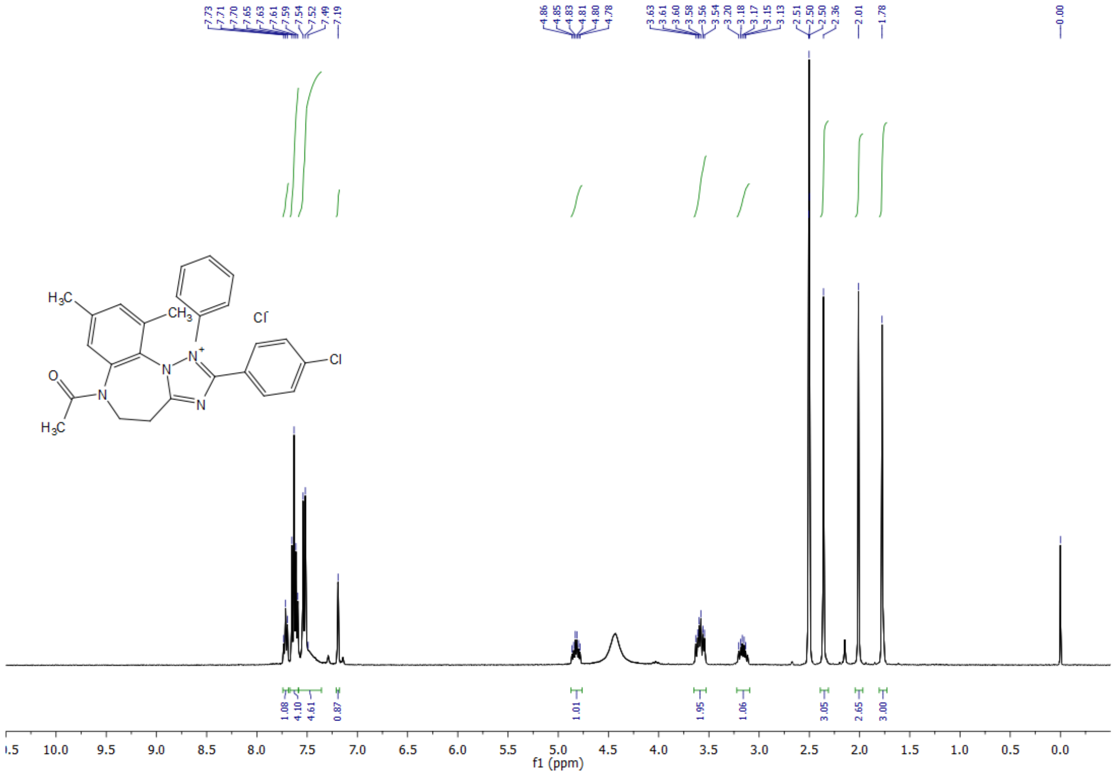

^{13}C NMR spectrum of **10o**

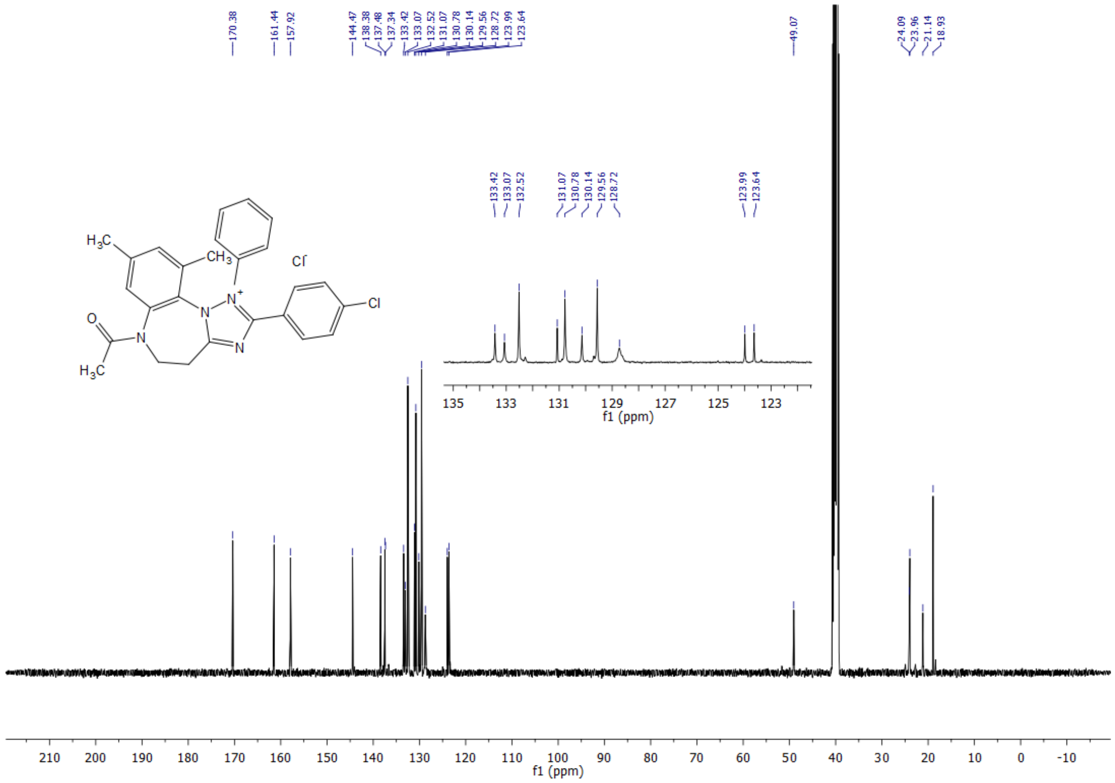

^{1}H NMR spectrum of **10p**

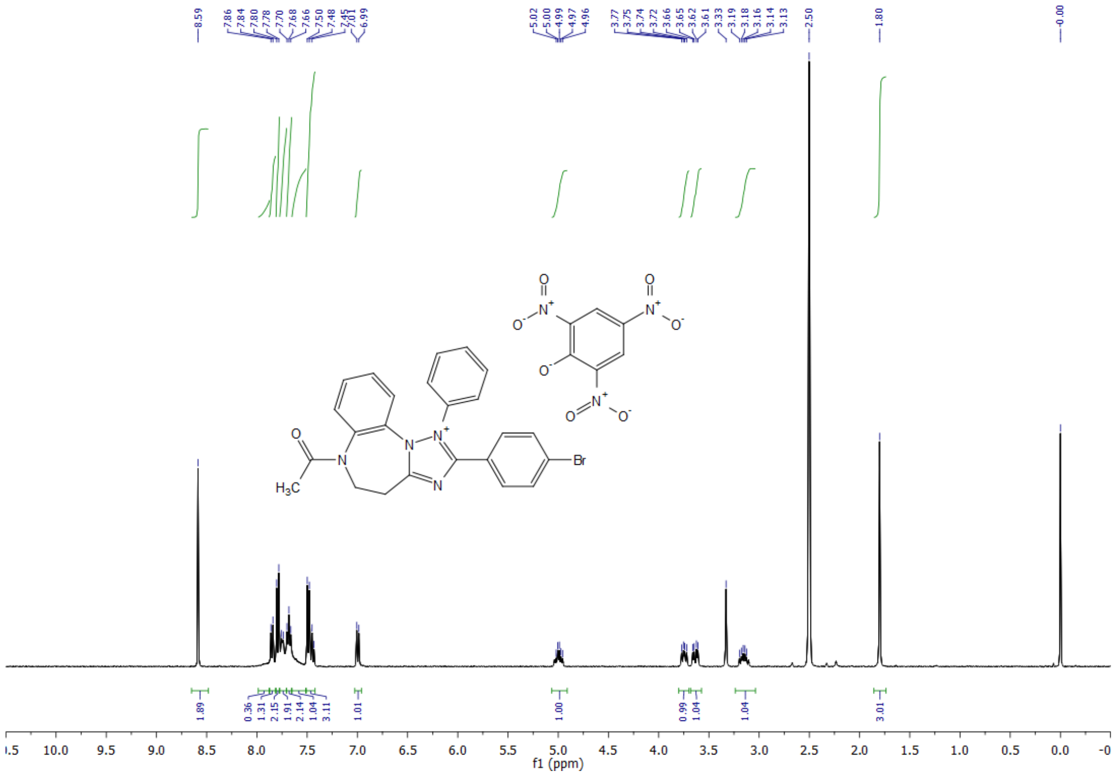

^{13}C NMR spectrum of **10p**

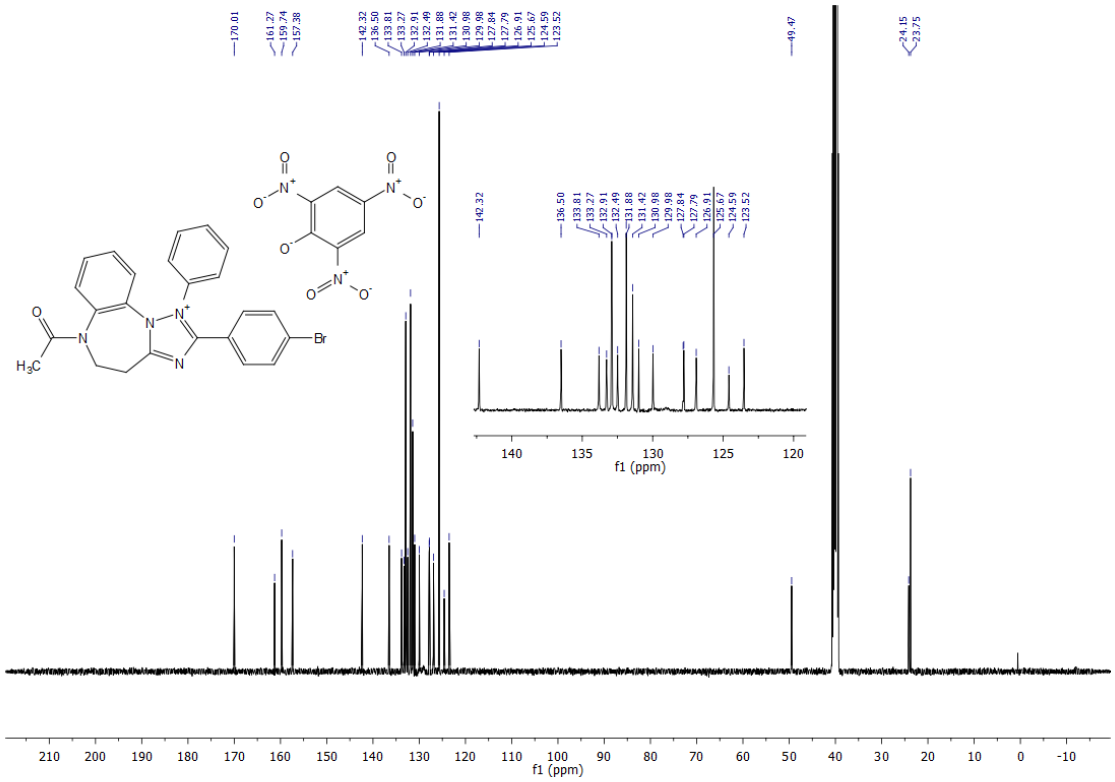

^{1}H NMR spectrum of **10q**

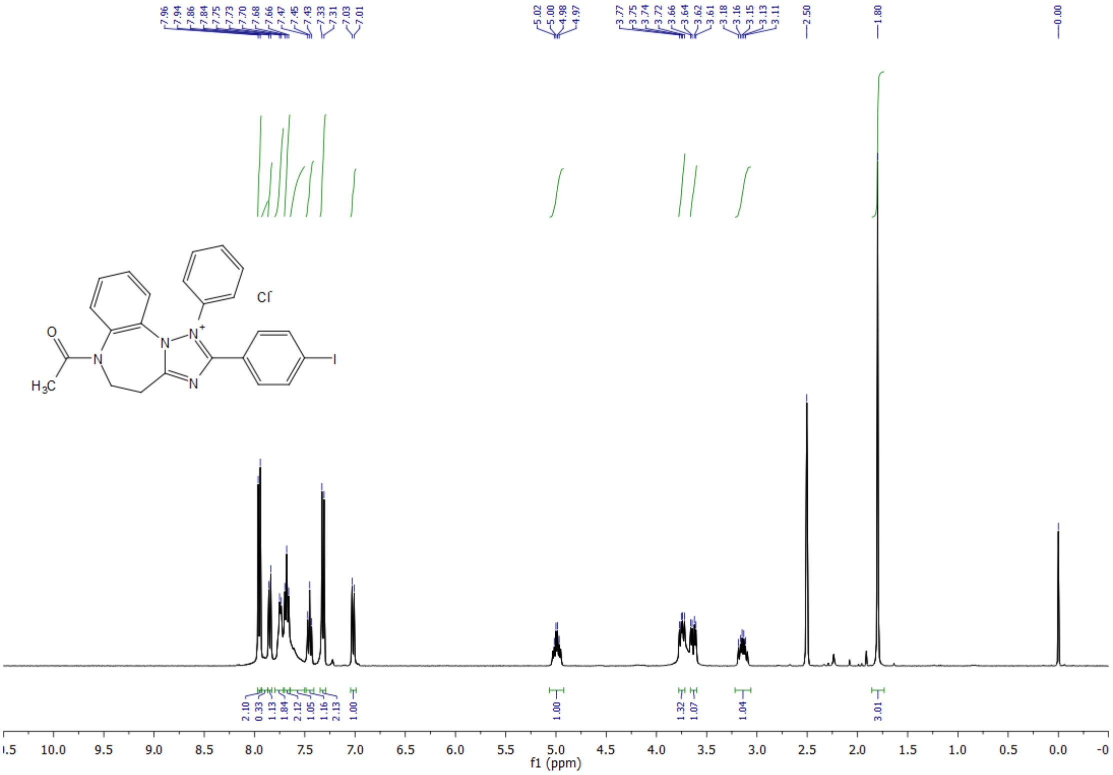

^{13}C NMR spectrum of **10q**

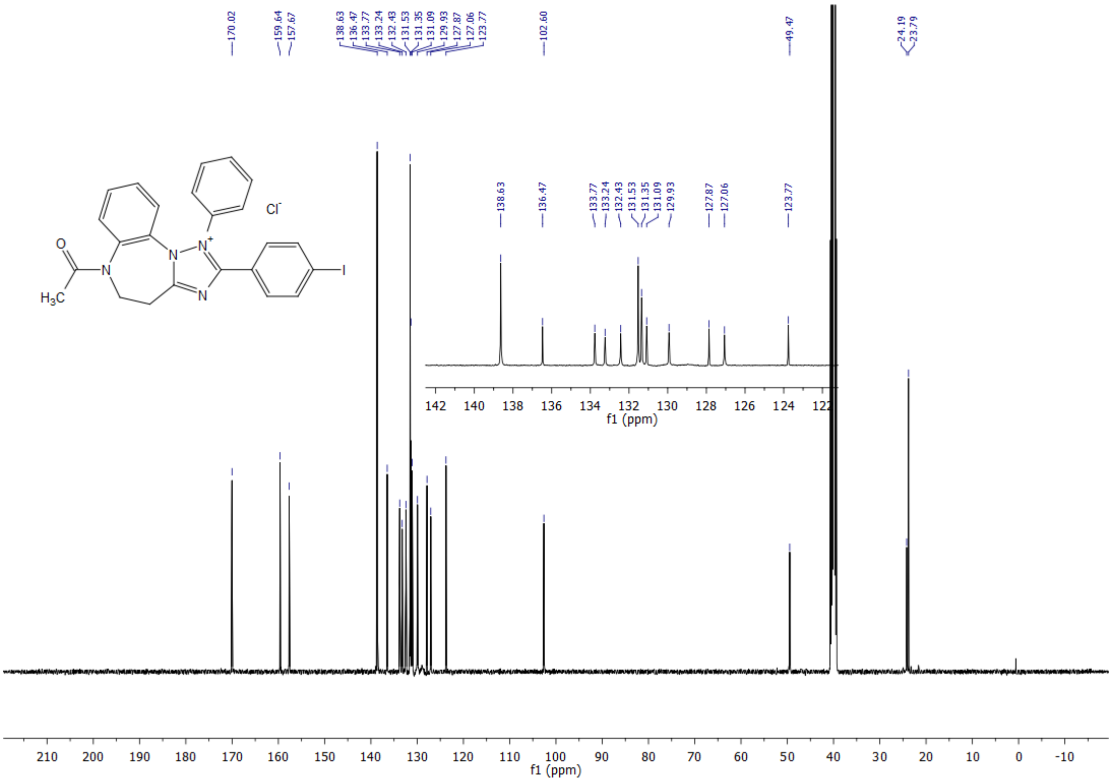

^{1}H NMR spectrum of **10r**

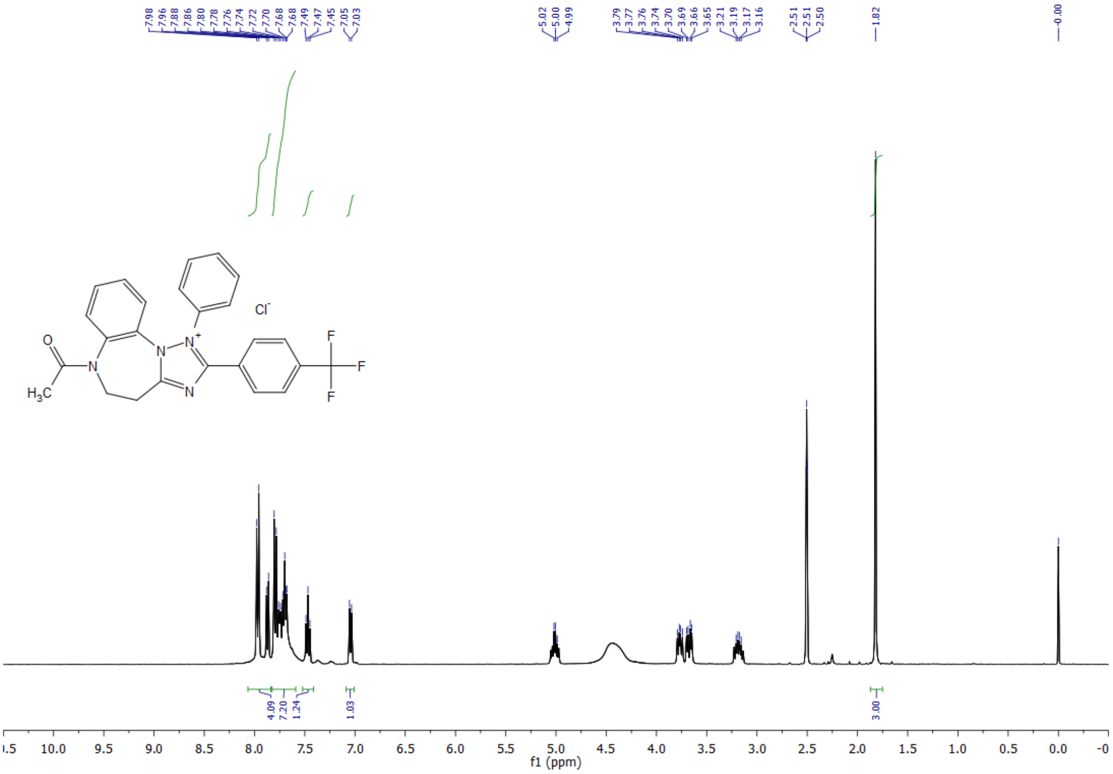

^{13}C NMR spectrum of **10r**

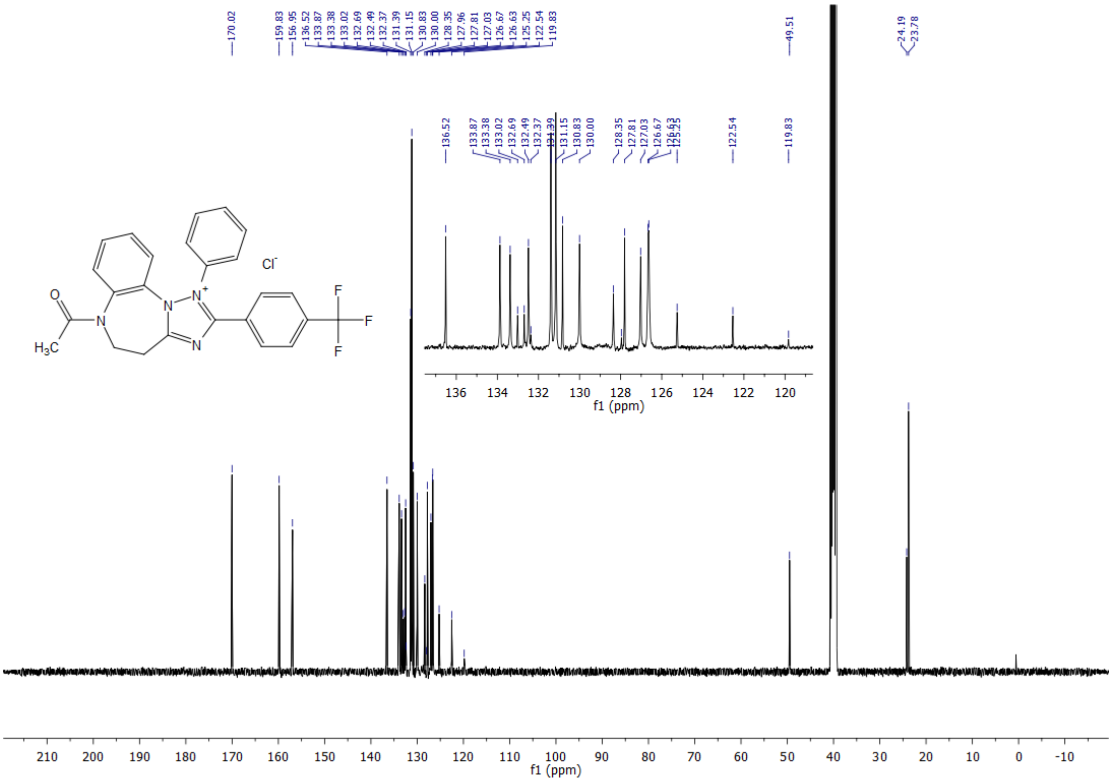

^{1}H NMR spectrum of **10t**

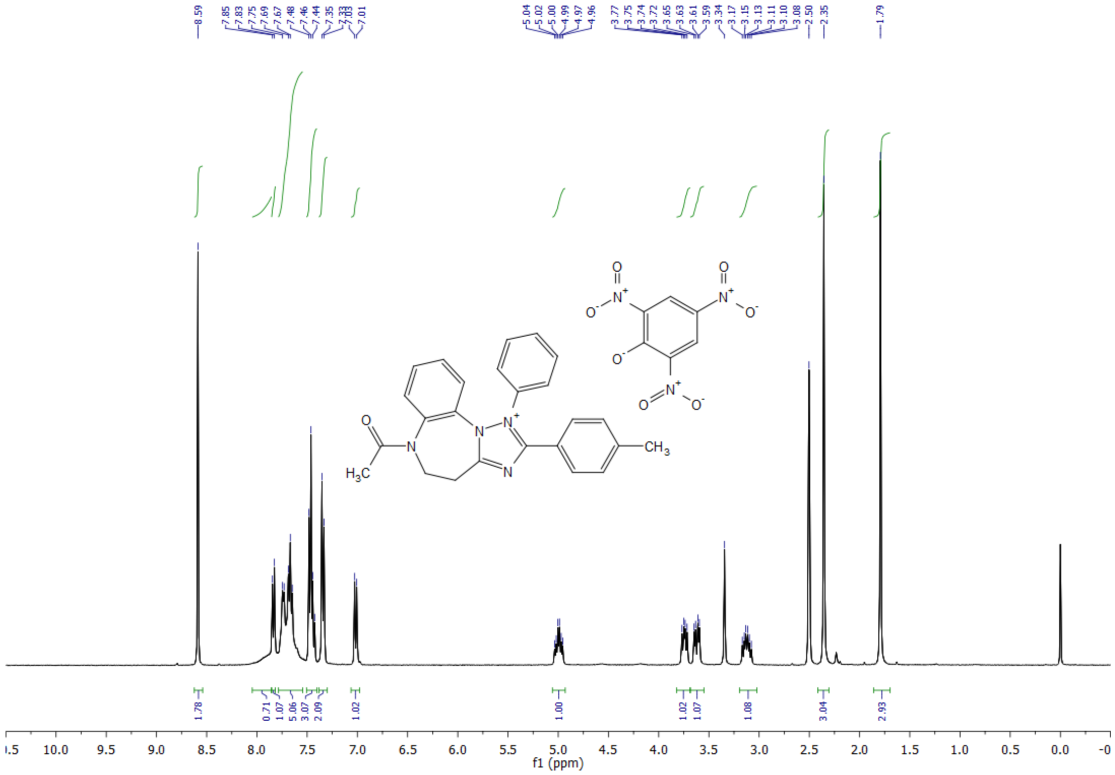

^{13}C NMR spectrum of **10t**

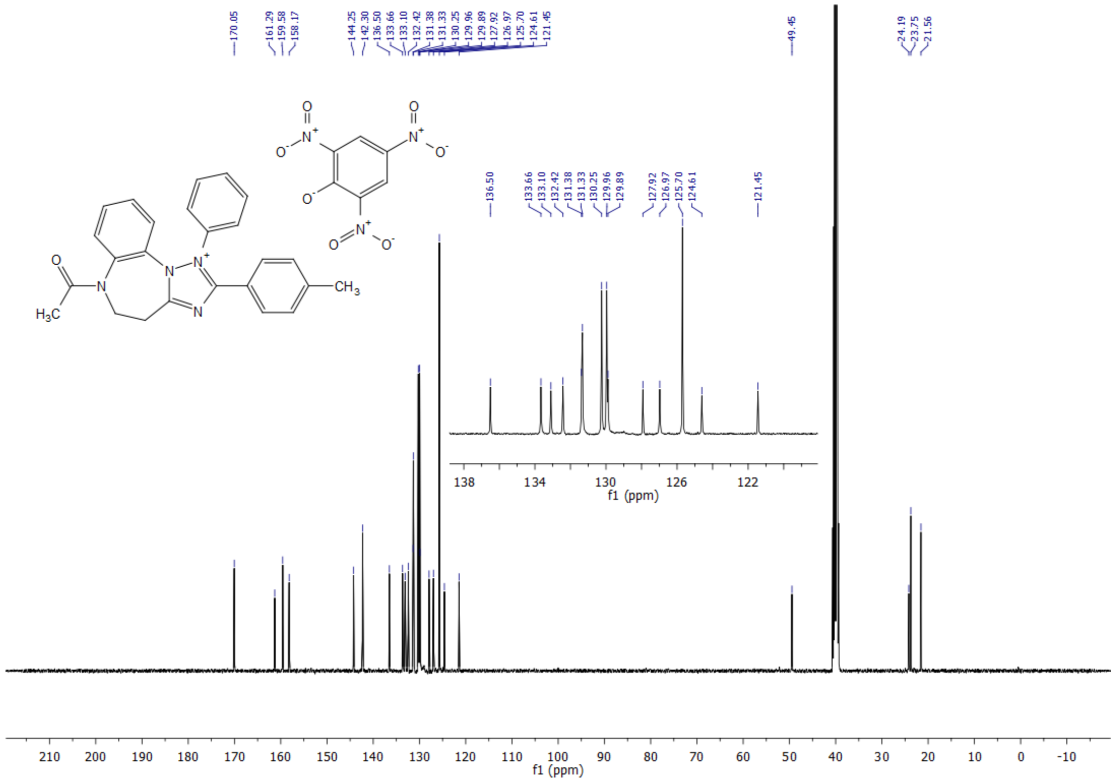

^{1}H NMR spectrum of **10u**

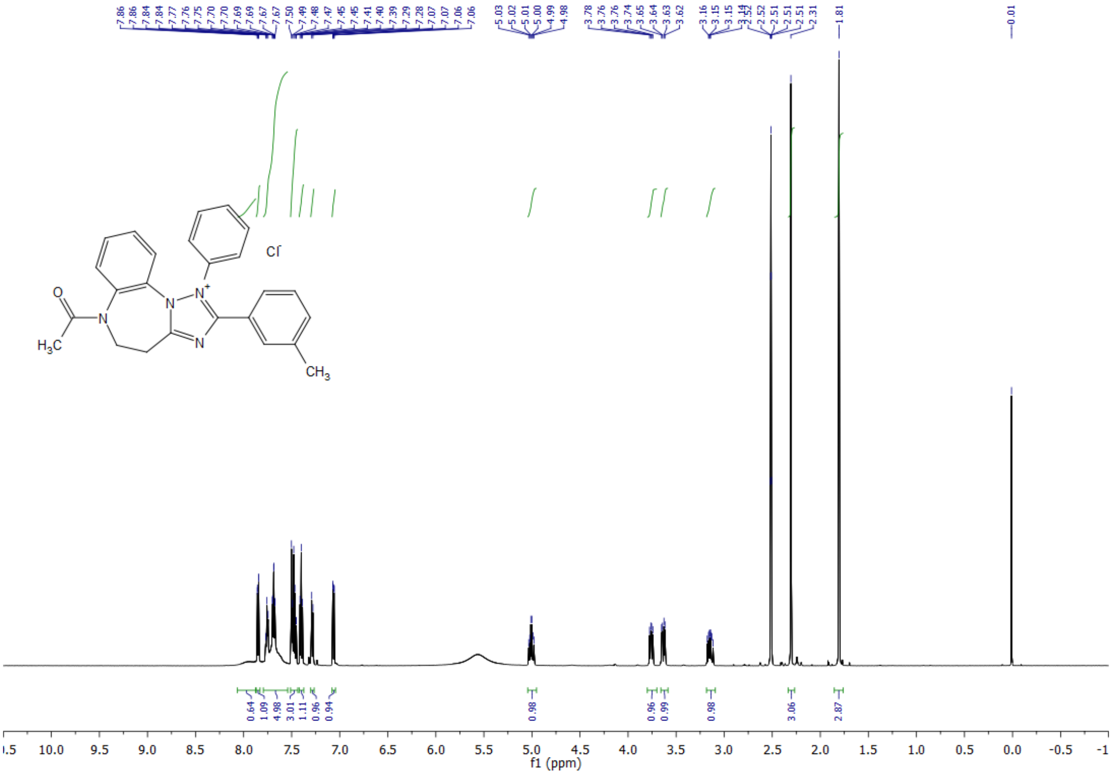

^{13}C NMR spectrum of **10u**

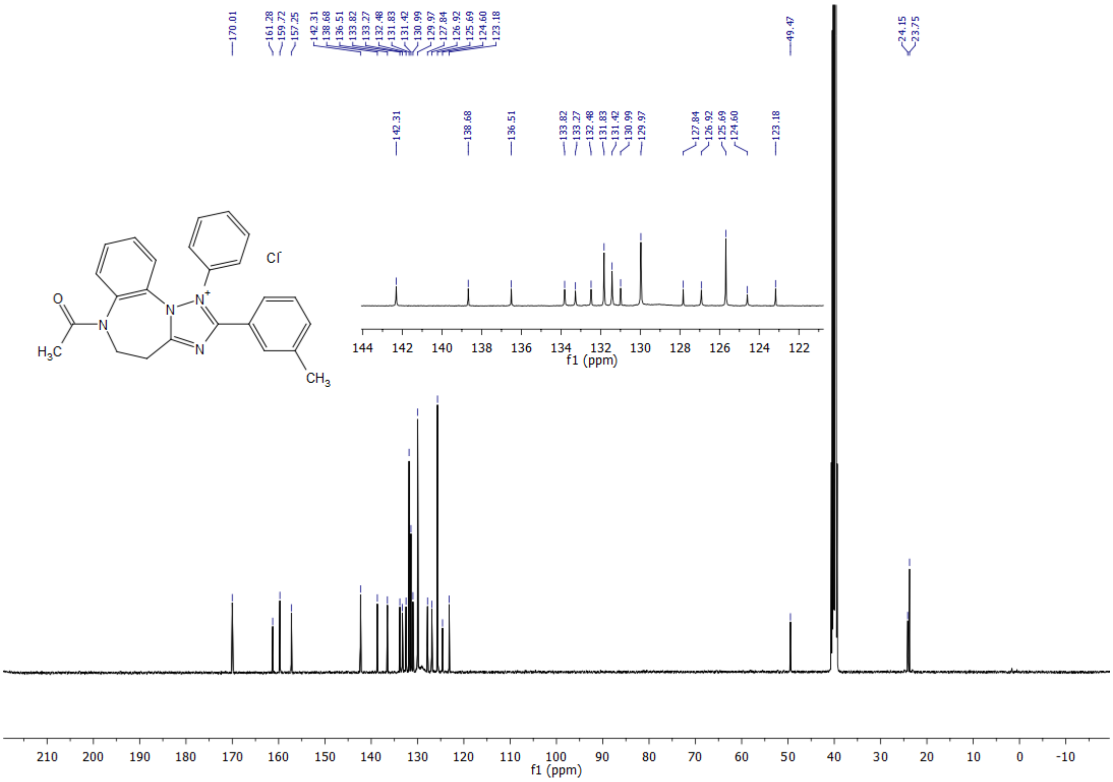

^{1}H NMR spectrum of **10v**

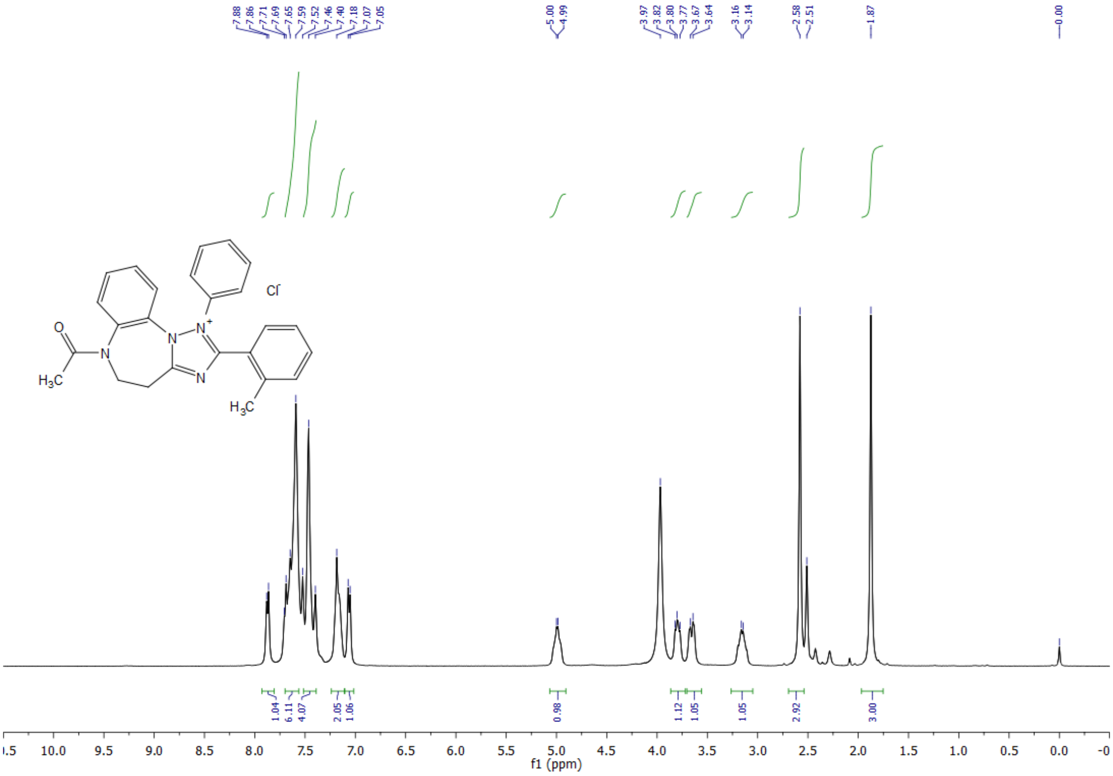

^{13}C NMR spectrum of **10v**

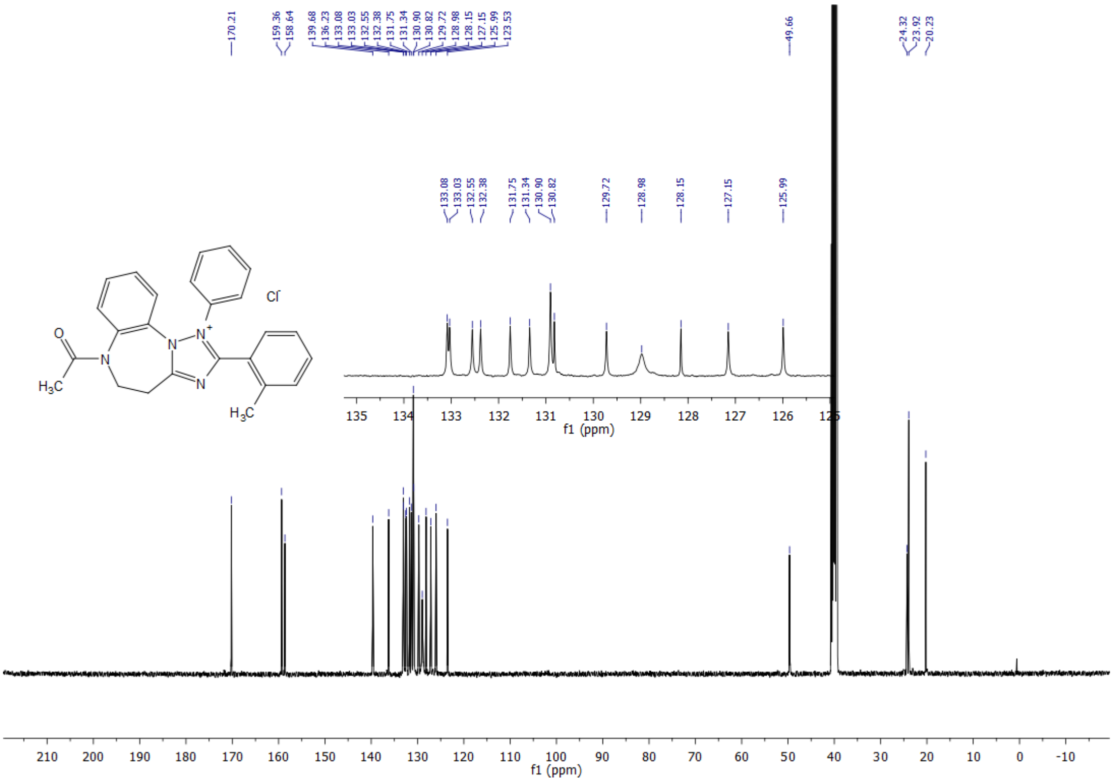

^{1}H NMR spectrum of **10w**

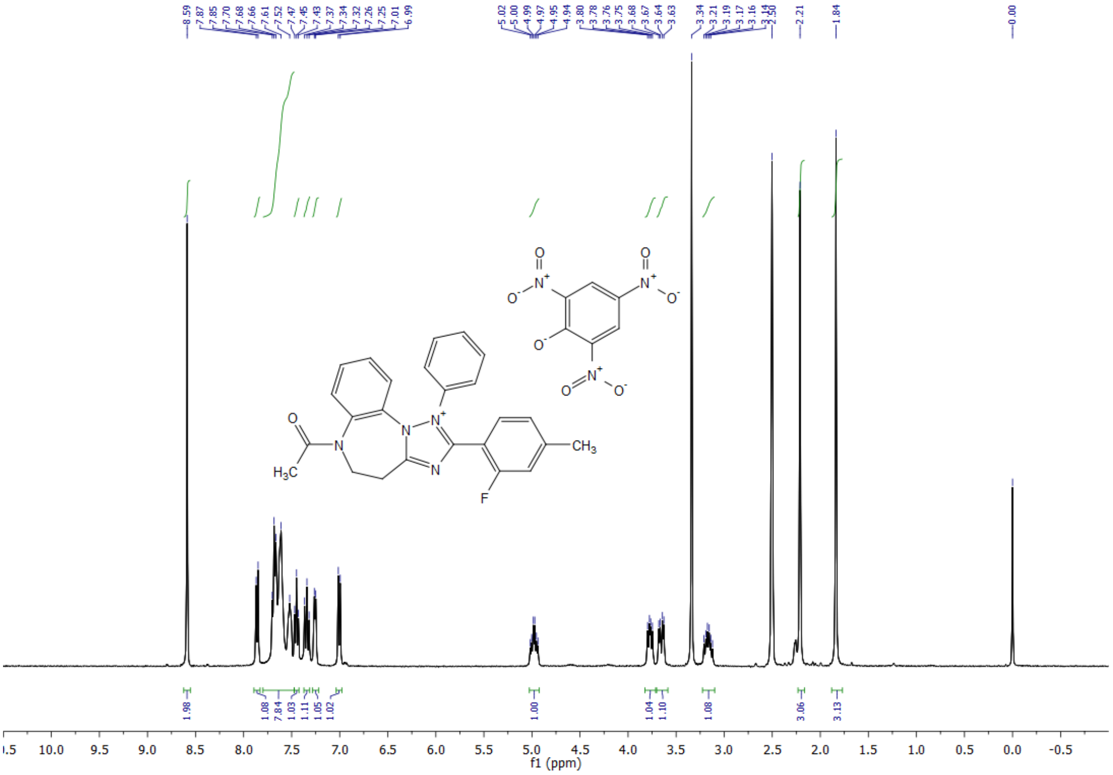

^{13}C NMR spectrum of **10w**

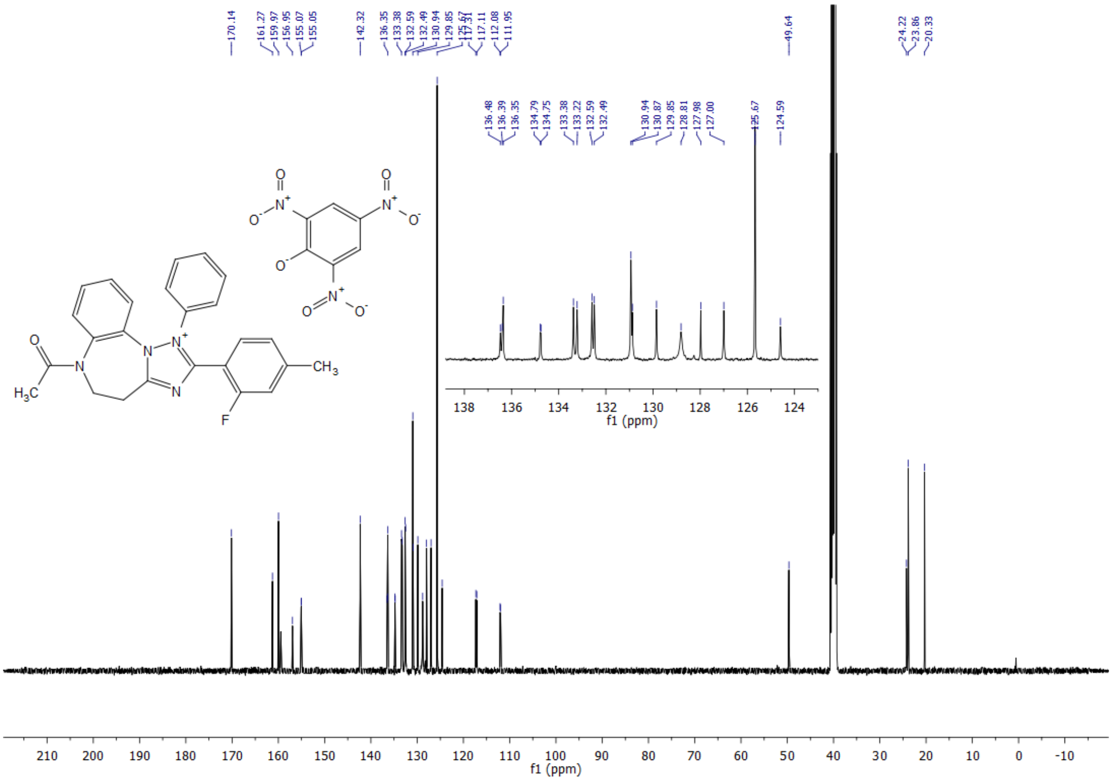

^{1}H NMR spectrum of **10x**

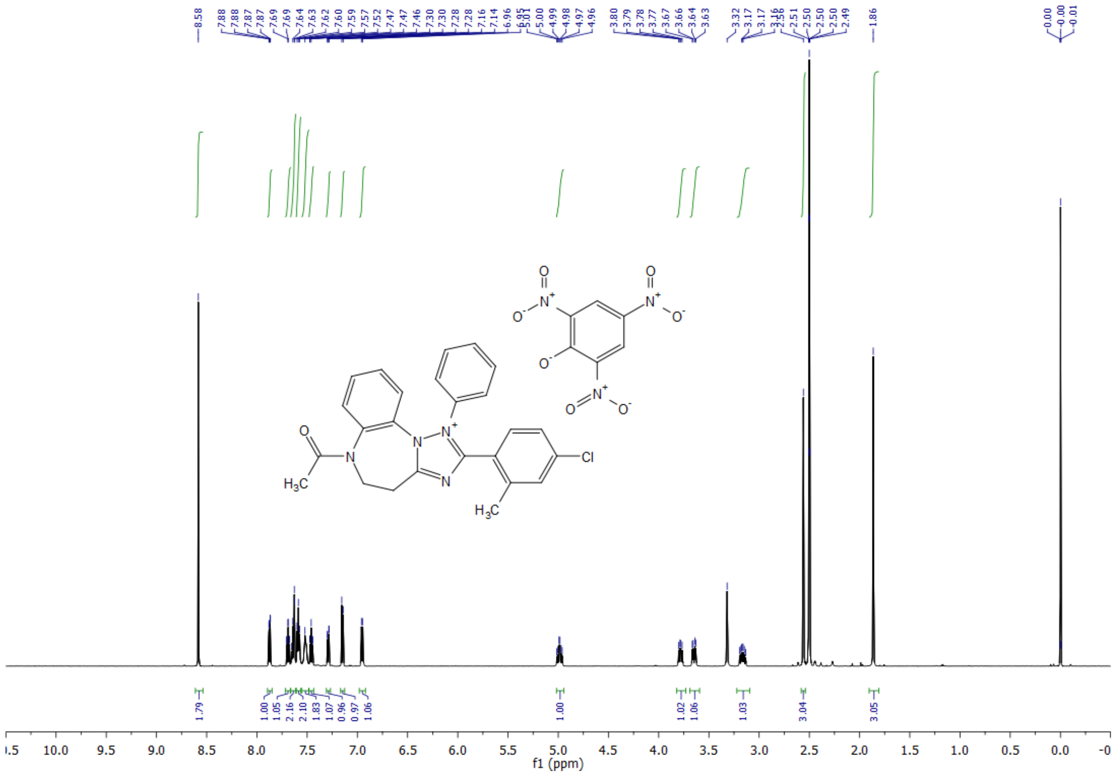

^{13}C NMR spectrum of **10x**

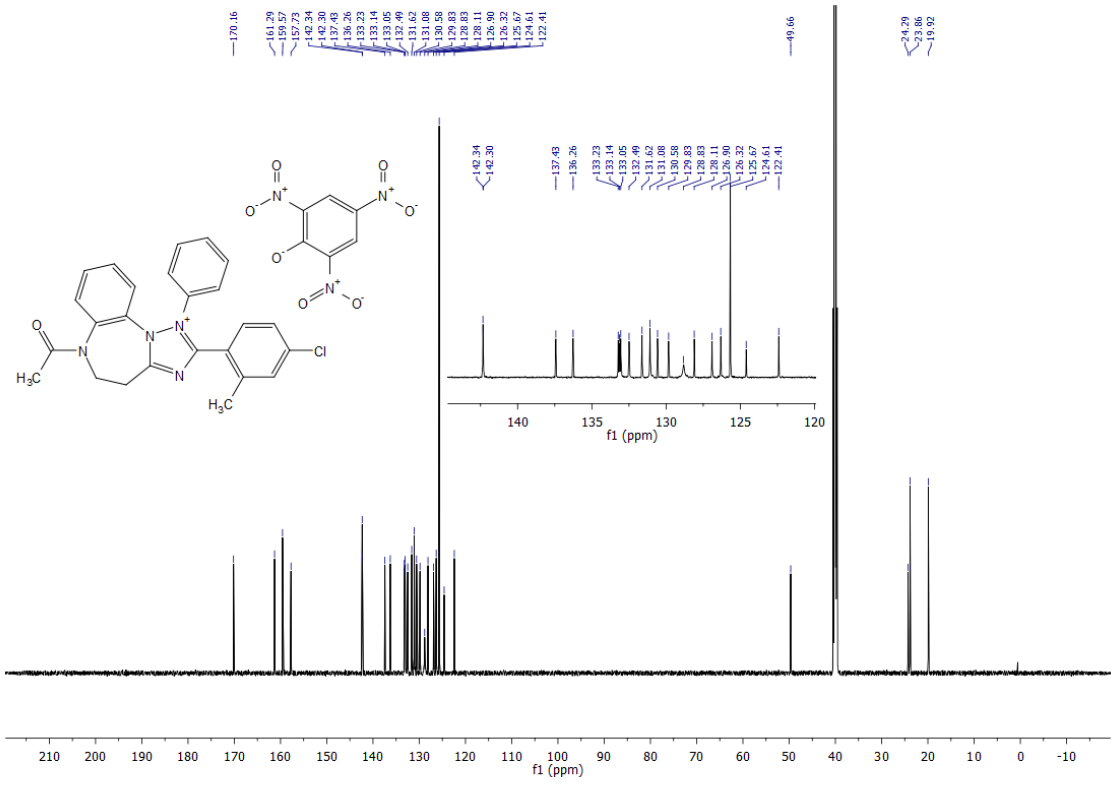

^{1}H NMR spectrum of **11**

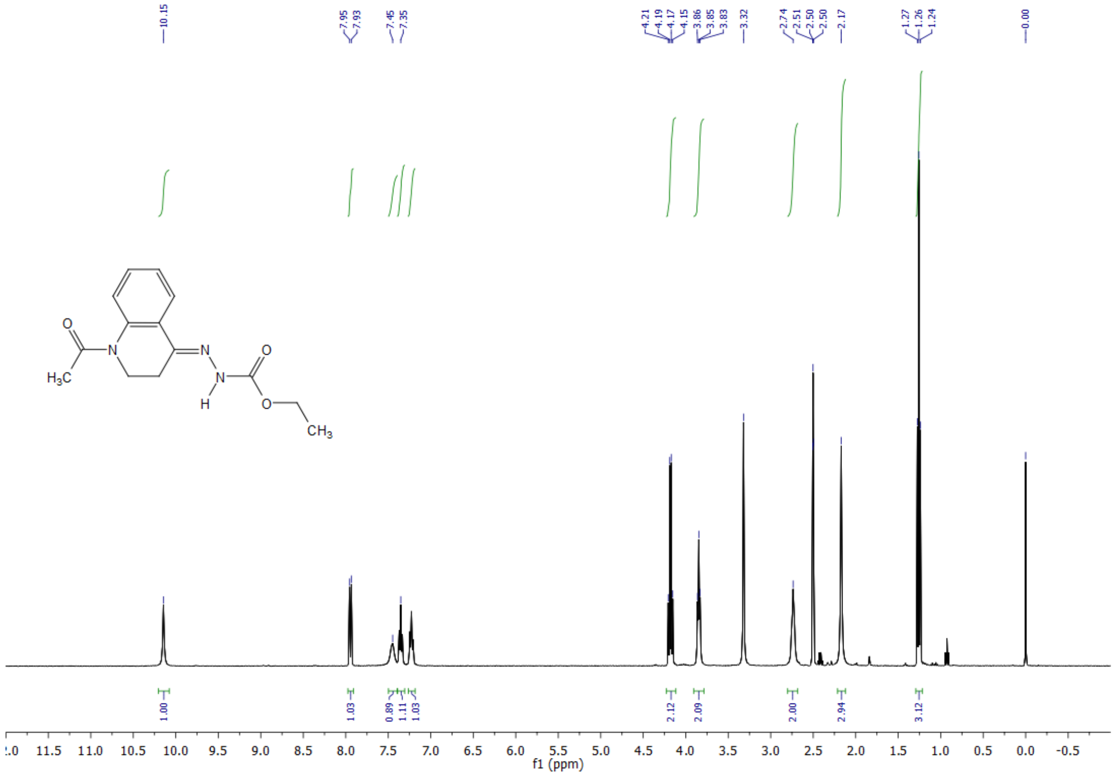

^{13}C NMR spectrum of **11**

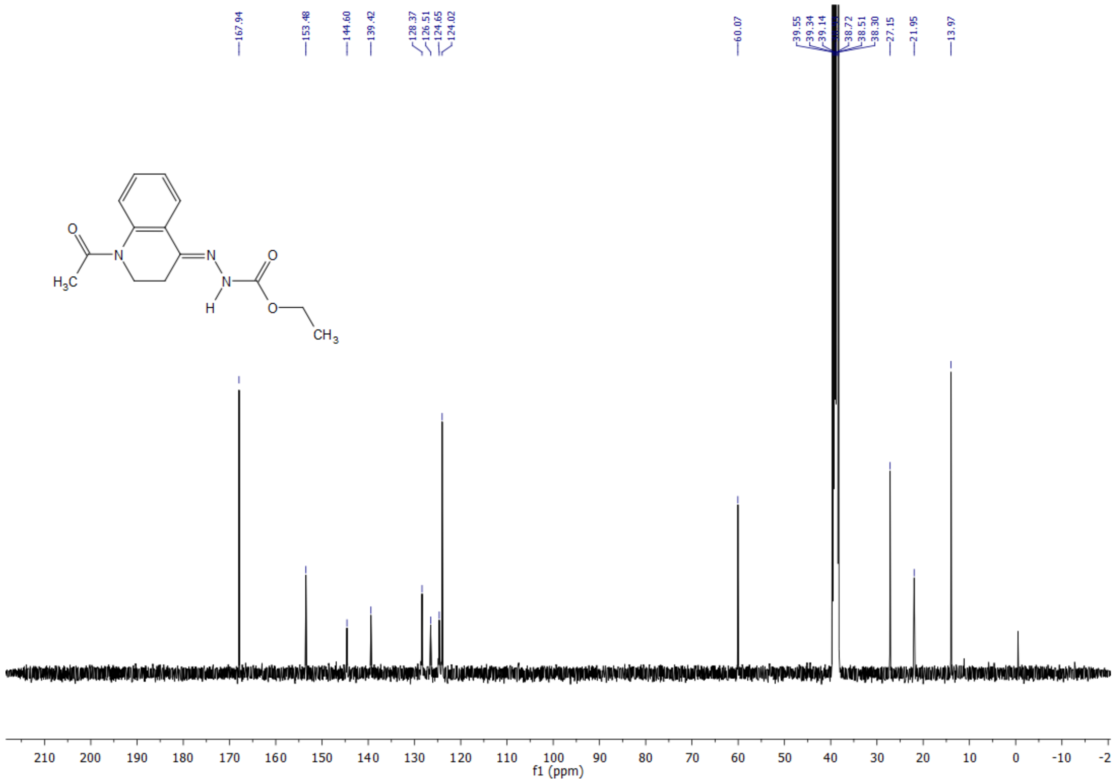

^{1}H NMR spectrum of **13a**

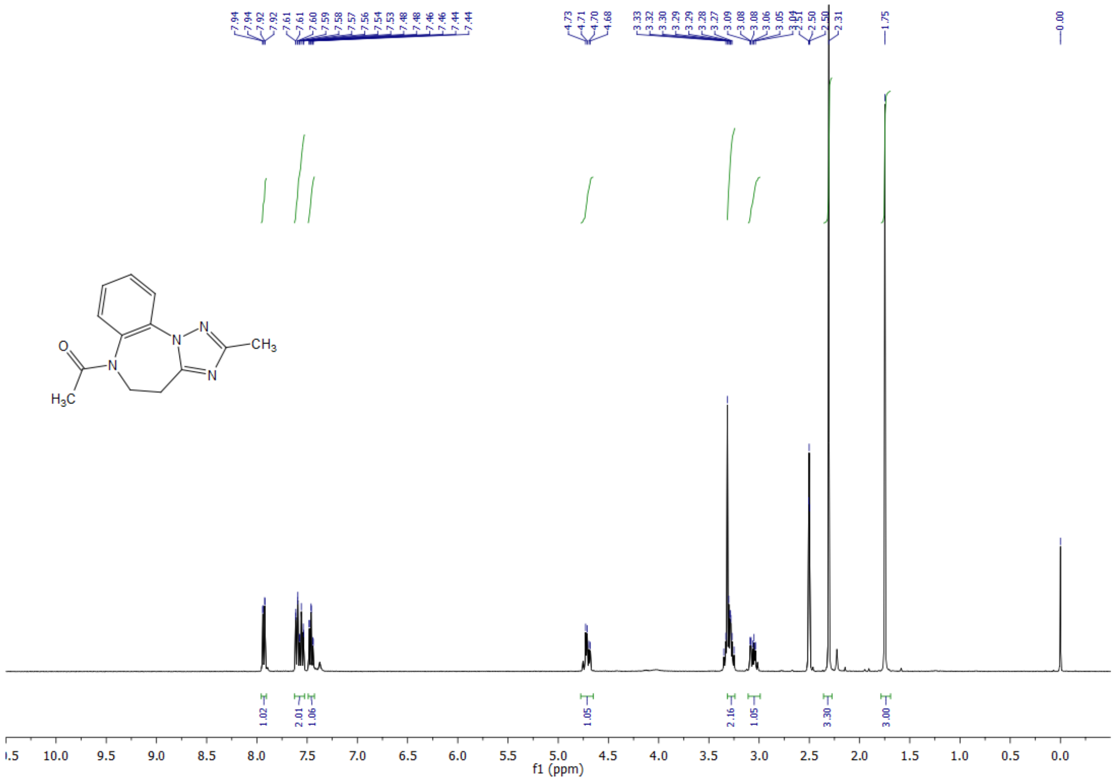

^{13}C NMR spectrum of **13a**

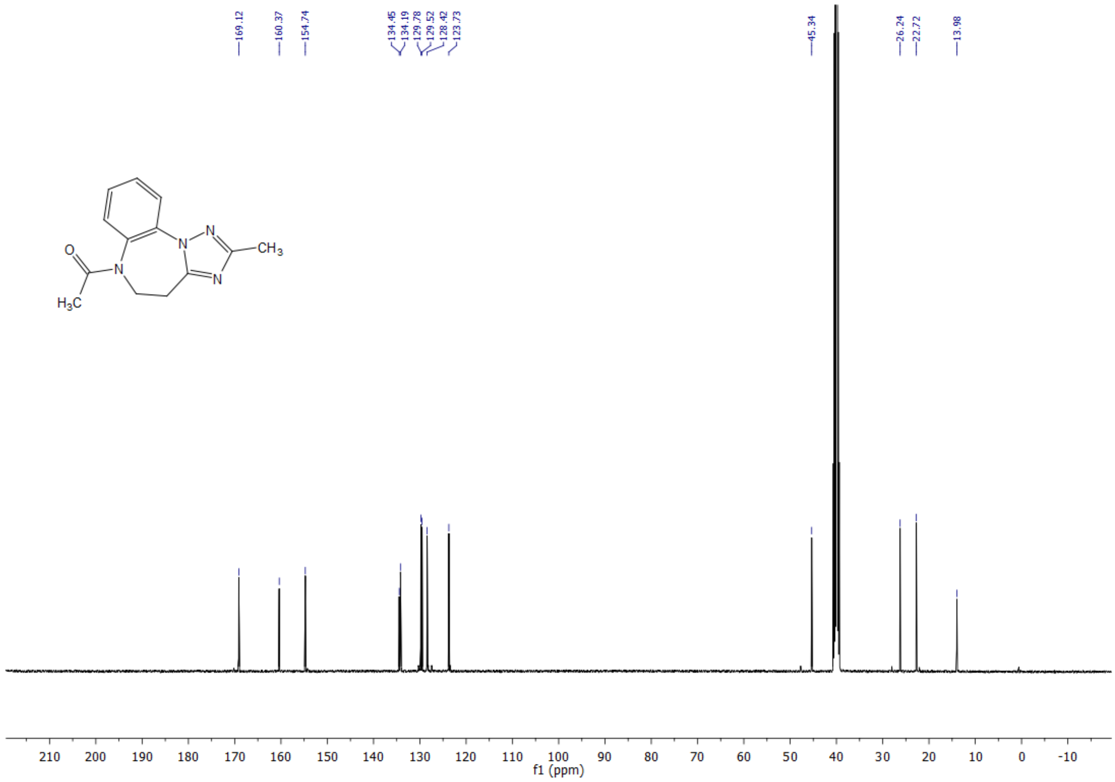

^{1}H NMR spectrum of **13b**

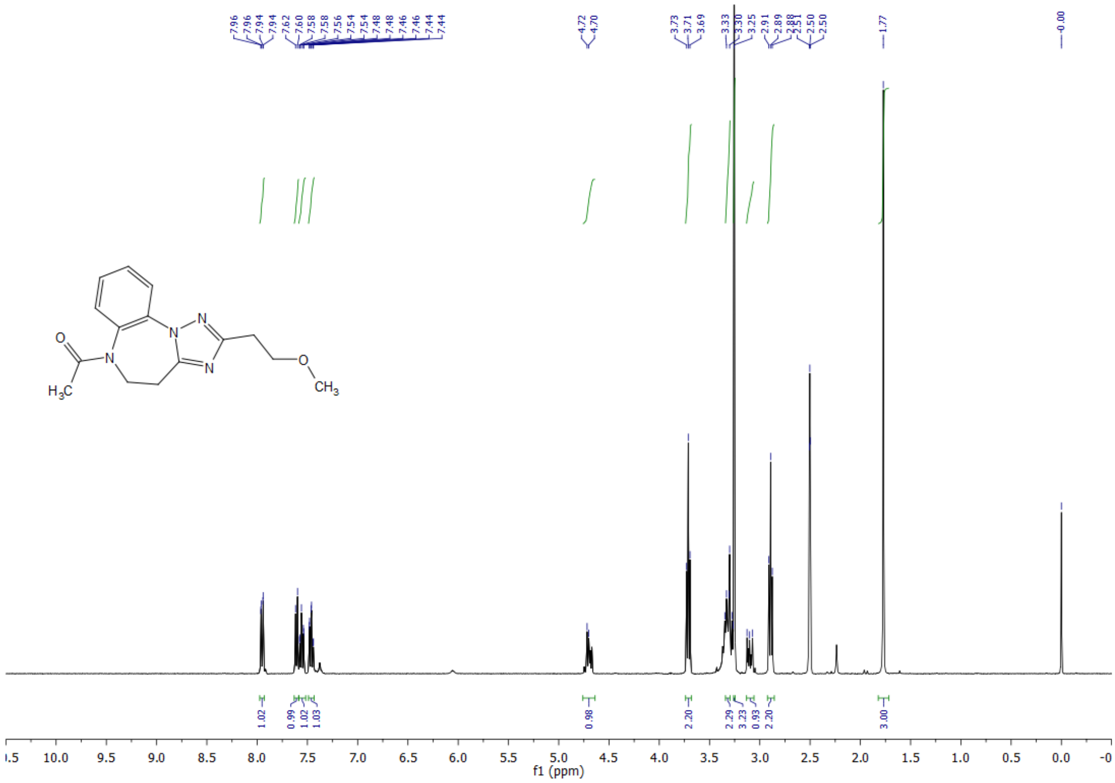

^{13}C NMR spectrum of **13b**

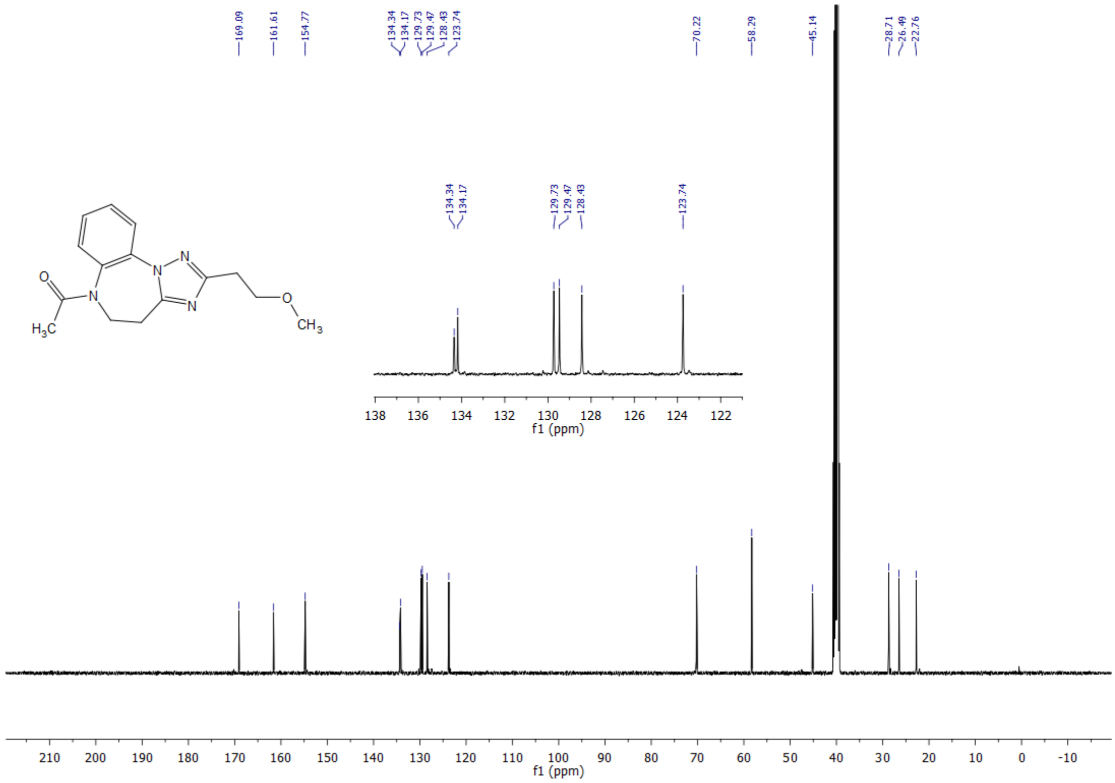

^{1}H NMR spectrum of **13c**

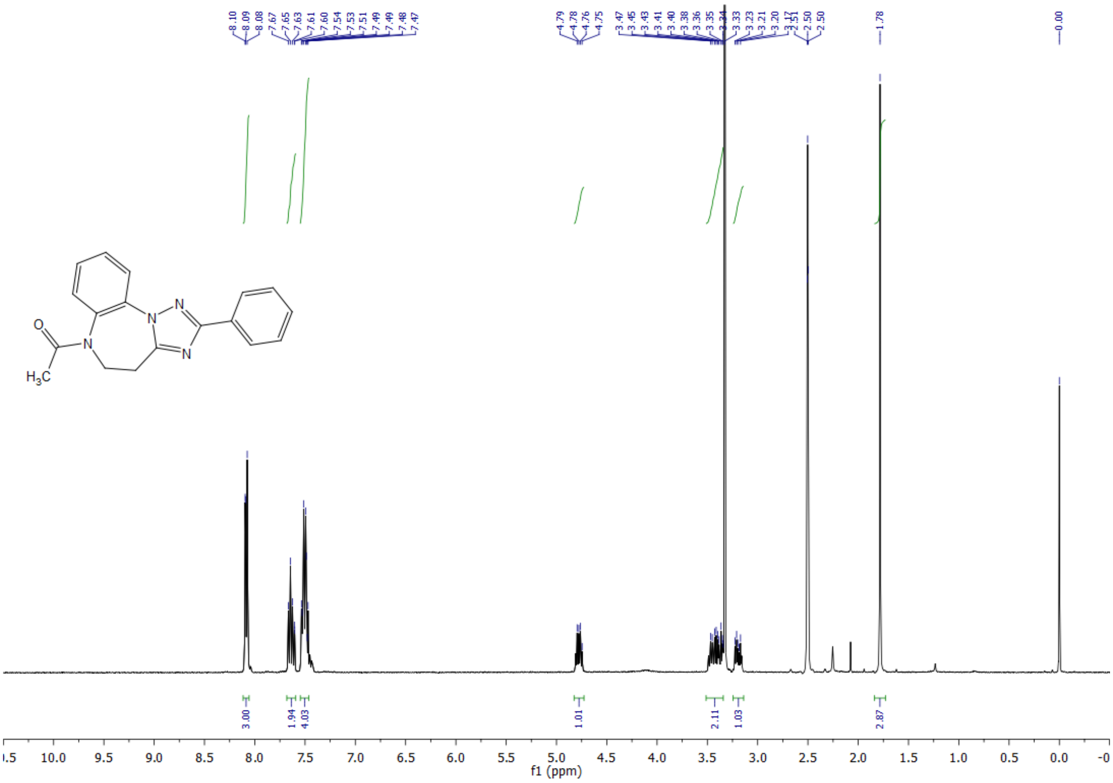

^{13}C NMR spectrum of **13c**

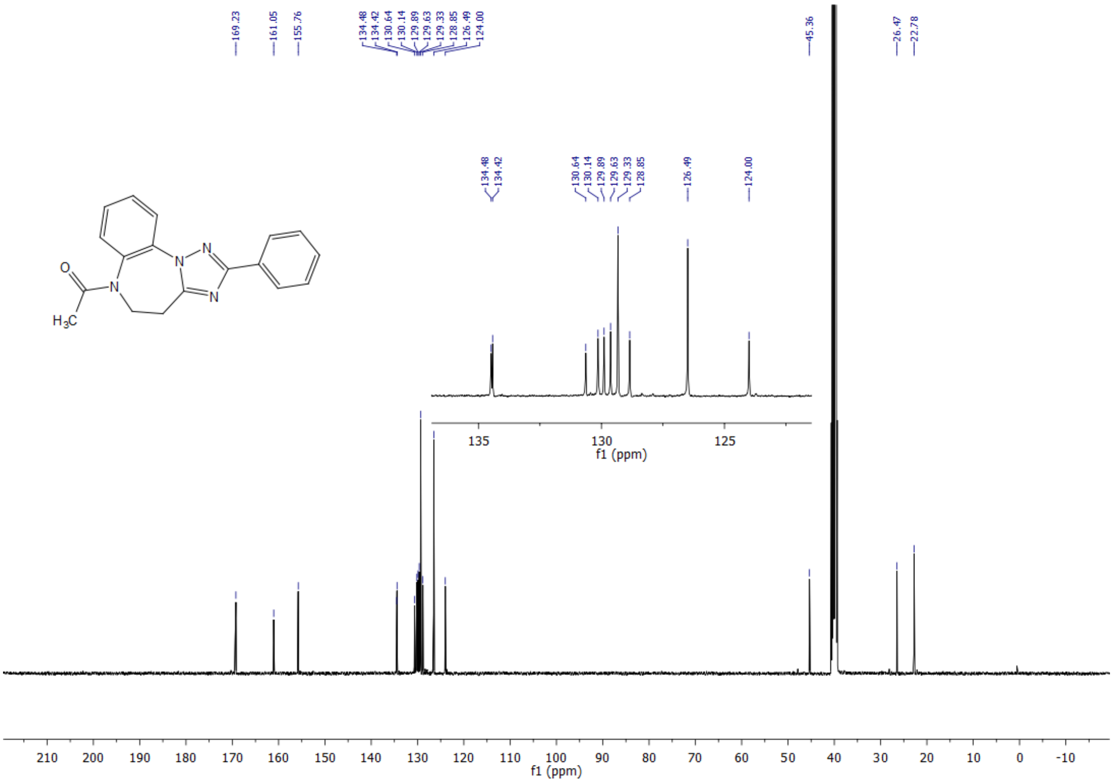

^{1}H NMR spectrum of **13d**

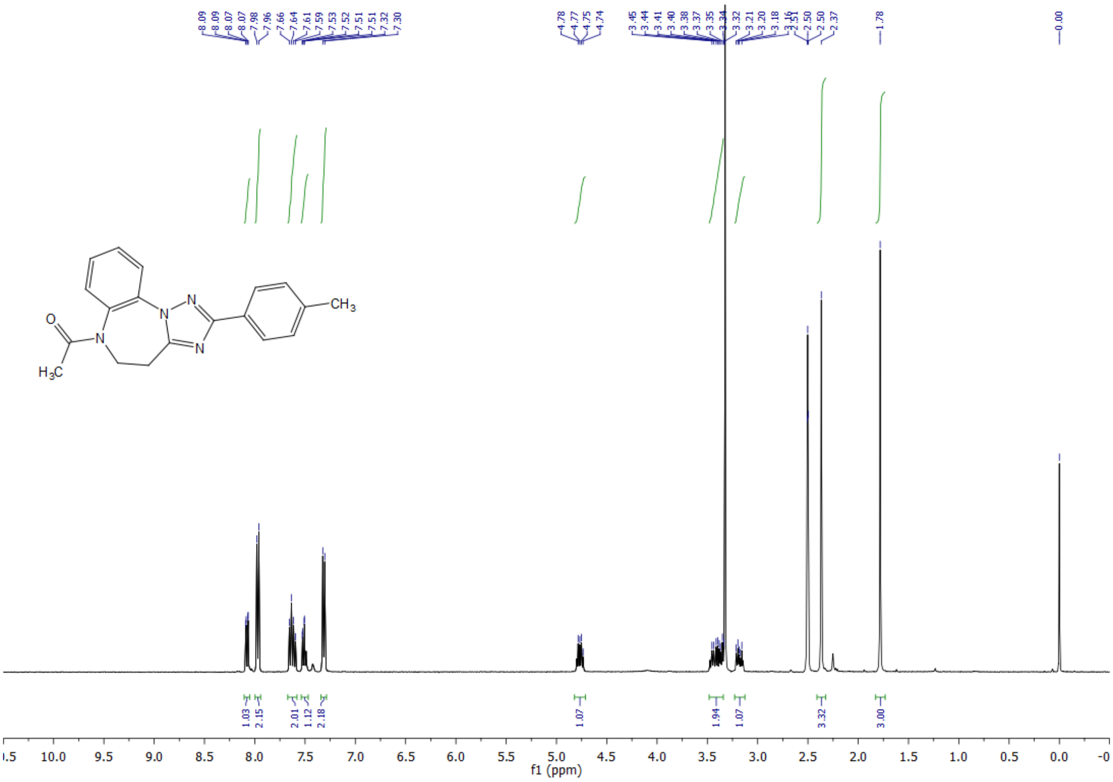

^{13}C NMR spectrum of **13d**

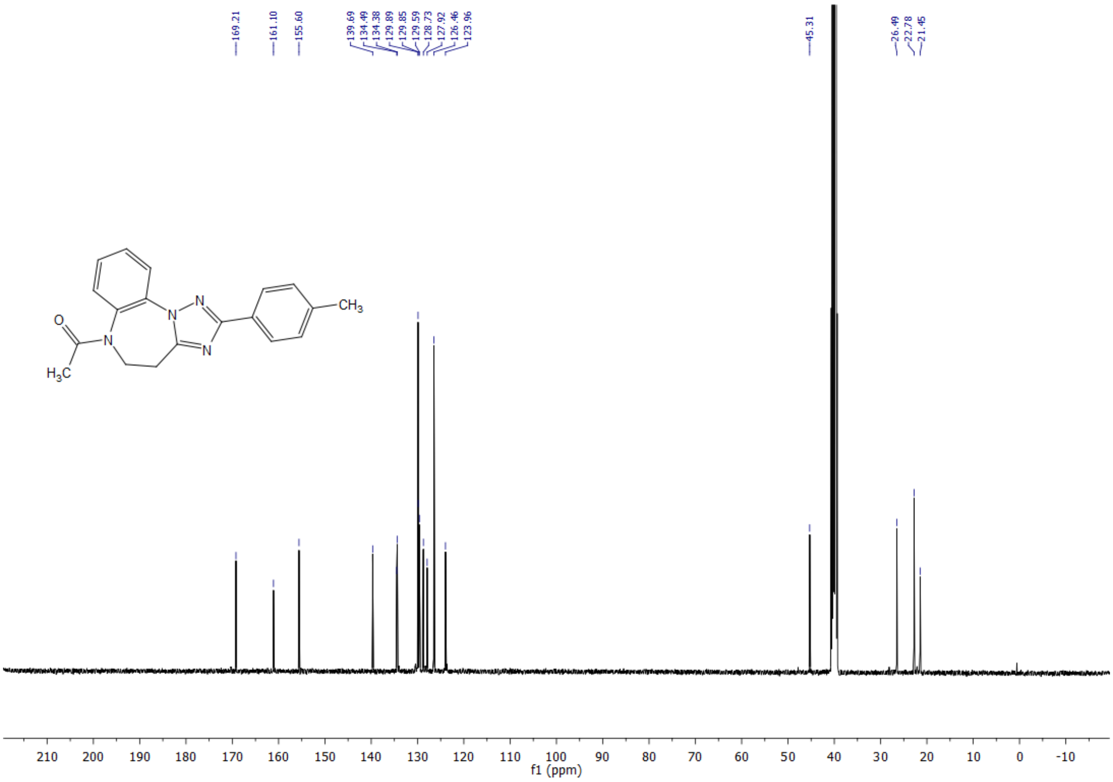

^{1}H NMR spectrum of **13e**

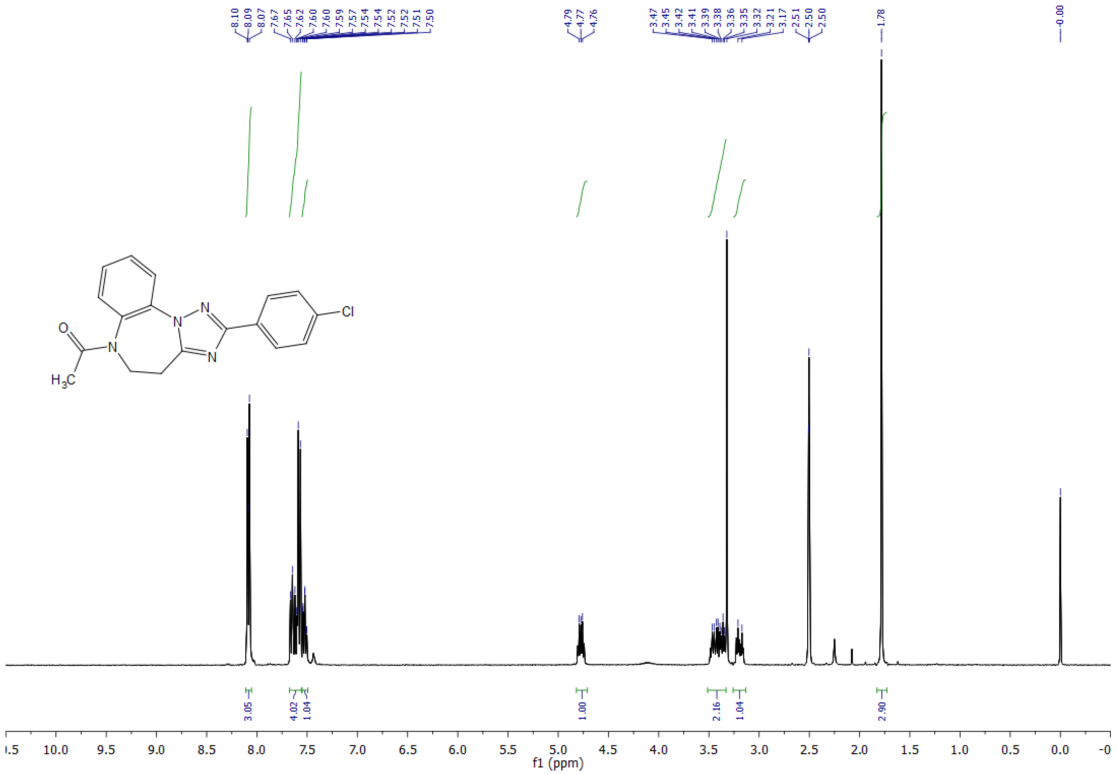

^{13}C NMR spectrum of **13e**

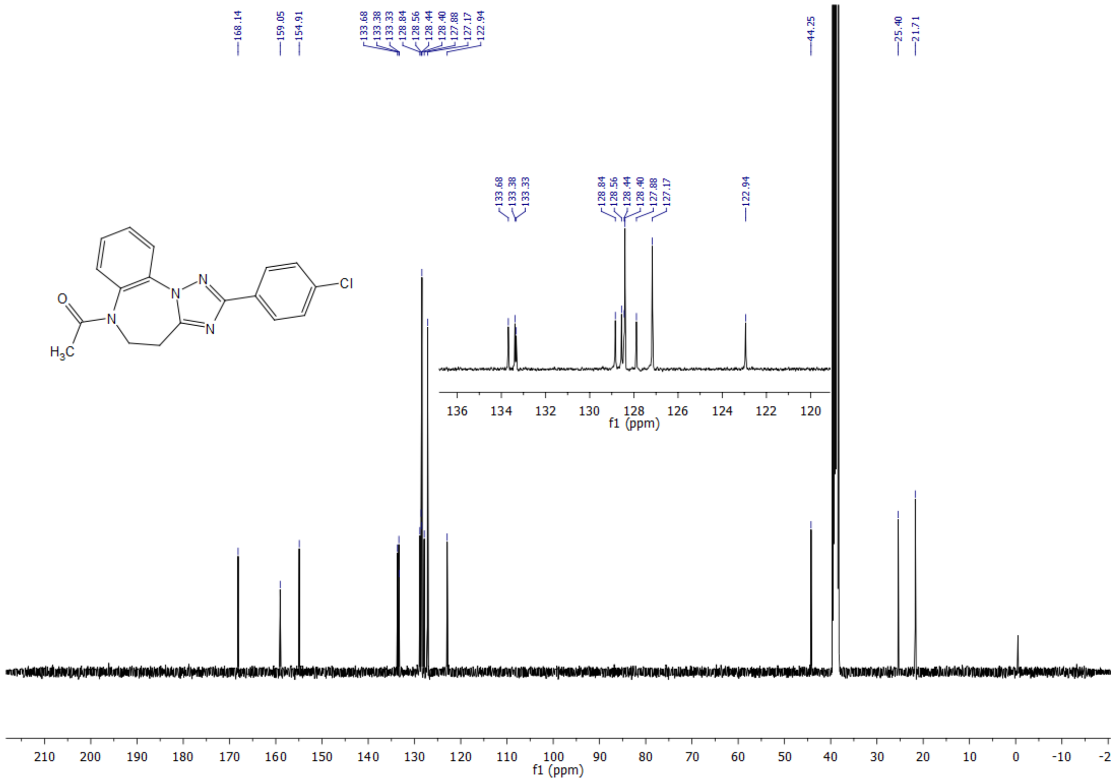

4. X-ray Crystal for compound 10k (anisotropic displacement parameters for ellipsoid contours are shown at 50% probability for all atoms except H)

The crystallographic data have been deposited with the Cambridge Crystallographic Data Centre (CCDC) as CCDC-1438637. CCDC information can be obtained free of charge from www.ccdc.cam.ac.uk. The detailed crystallographic data are summarized in Tables S1 and Table S2.

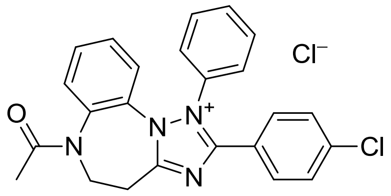

10k

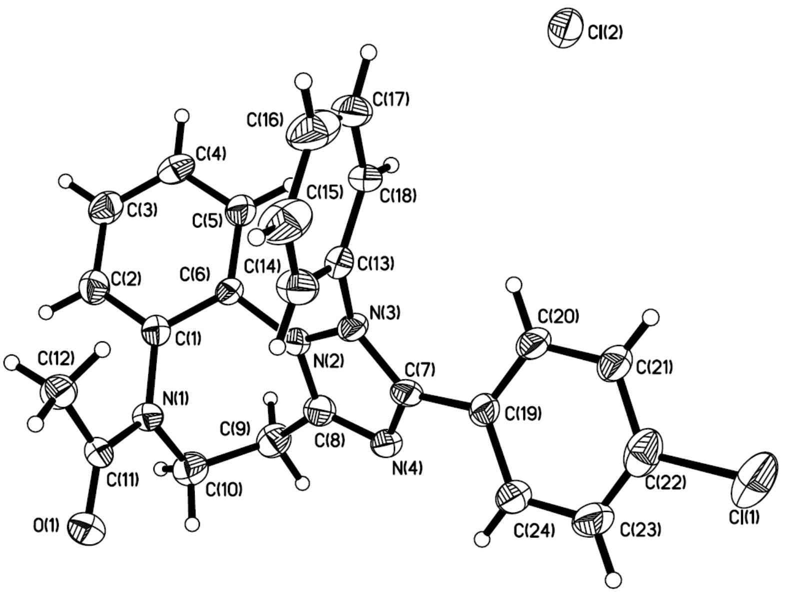

Table S1: Crystal data and structure refinement for **10k**.

Identification code	**10k**
Empirical formula	C24 H20 Cl2 N4 O
Formula weight	451.34
Temperature	293(2) K
Wavelength	0.71073 Å

Crystal system	Monoclinic
Space group	C 2/c
Unit cell dimensions	a = 22.048(11) Å α = 90°.
	b = 11.951(6) Å β = 106.792(7)°.
	c = 19.498(10) Å γ = 90°.
Volume	4919(4) Å^3
Z	8
Density (calculated)	1.219 Mg/m^3
Absorption coefficient	0.286 mm^{-1}
F(000)	1872
Crystal size	0.010 x 0.010 x 0.010 mm^3
Theta range for data collection	1.930 to 25.245°.
Index ranges	-26<=h<=13, -14<=k<=14, -22<=l<=23
Reflections collected	10249
Independent reflections	4443 [R(int) = 0.0955]
Completeness to theta = 25.242°	99.8 %
Absorption correction	Semi-empirical from equivalents
Max. and min. transmission	1.000 and 0.459
Refinement method	Full-matrix least-squares on F^2
Data / restraints / parameters	4443 / 0 / 281

Goodness-of-fit on F^2	1.071
Final R indices [I>2sigma(I)]	R1 = 0.1178, wR2 = 0.2654
R indices (all data)	R1 = 0.1664, wR2 = 0.2851
Extinction coefficient	n/a
Largest diff. peak and hole	0.339 and -0.343 e.Å^{-3}

Table S2: Datablock: 10k

Bond precision: C-C = 0.0110 A Wavelength=0.71073

Cell: a=22.048(11) b=11.951(6) c=19.498(10)

alpha=90 beta=106.792(7) gamma=90

Temperature: 293 K

Calculated Reported

Volume 4919(4) 4919(4)

Space group C 2/c C 2/c

Hall group -C 2yc -C 2yc

Moiety formula C24 H20 Cl N4 O, Cl ?

Sum formula C24 H20 Cl2 N4 O C24 H20 Cl2 N4 O

Mr 451.34 451.34

Dx,g cm-3 1.219 1.219

Z 8 8

Mu (mm-1) 0.285 0.286

F000 1872.0 1872.0

F000’ 1874.96

h,k,lmax 26,14,23 26,14,23

Nref 4449 4443

Tmin,Tmax 0.997,0.997 0.459,1.000

Tmin' 0.997

Correction method= MULTI-SCAN

Data completeness= 0.999 Theta(max)= 25.245

R(reflections)= 0.1178(2705) wR2(reflections)= 0.2851(4443)

S = 1.071 Npar= 281

5. X-ray Crystal for compound 13e (anisotropic displacement parameters for ellipsoid contours are shown at 50% probability for all atoms except H)

The crystallographic data have been deposited with the Cambridge Crystallographic Data Centre (CCDC) as CCDC-1438255. CCDC information can be obtained free of charge from www.ccdc.cam.ac.uk. The detailed crystallographic data are summarized in Tables S1-2.

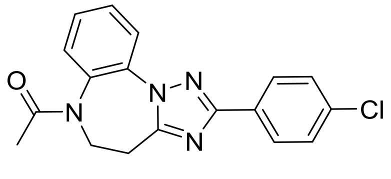

13e

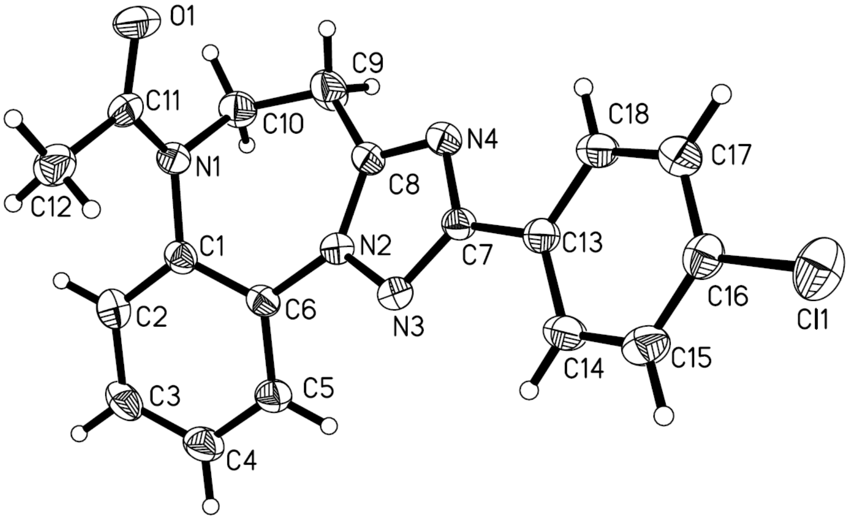

Table S3: Crystal data and structure refinement for **13e**.

Identification code	13e
Empirical formula	C18 H15 Cl N4 O
Formula weight	338.79
Temperature	293(2) K
Wavelength	0.71073 Å
Crystal system	Monoclinic

Space group	P 21/c	
Unit cell dimensions	a = 10.752(5) Å	α = 90°.
	b = 24.068(11) Å	β = 100.772(6)°.
	c = 12.529(6) Å	γ = 90°.
Volume	3185(3) $Å^3$	
Z	8	
Density (calculated)	1.413 Mg/m^3	
Absorption coefficient	0.252 mm^{-1}	
F(000)	1408	
Crystal size	0.660 x 0.260 x 0.240 mm^3	
Theta range for data collection	1.692 to 26.000°.	
Index ranges	-13<=h<=12, -29<=k<=29, -15<=l<=15	
Reflections collected	14470	
Independent reflections	6198 [R(int) = 0.0651]	
Completeness to theta = 25.242°	99.2 %	
Absorption correction	Semi-empirical from equivalents	
Max. and min. transmission	1.000 and 0.544	
Refinement method	Full-matrix least-squares on F^2	
Data / restraints / parameters	6198 / 0 / 435	
Goodness-of-fit on F^2	0.916	

Final R indices [I>2sigma(I)]	R1 = 0.0494, wR2 = 0.1248
R indices (all data)	R1 = 0.0730, wR2 = 0.1334
Extinction coefficient	n/a
Largest diff. peak and hole	0.347 and -0.273 e.Å^{-3}

Table S4: Datablock: 13e

Bond precision: C-C = 0.0030 A Wavelength=0.71073

Cell: a=10.752(5) b=24.068(11) c=12.529(6)

alpha=90 beta=100.772(6) gamma=90

Temperature: 293 K

Calculated Reported

Volume 3185(3) 3185(3)

Space group P 21/c P 21/c

Hall group -P 2ybc -P 2ybc

Moiety formula C18 H15 Cl N4 O ?

Sum formula C18 H15 Cl N4 O C18 H15 Cl N4 O

Mr 338.79 338.79

Dx,g cm-3 1.413 1.413

Z 8 8

Mu (mm-1) 0.252 0.252

F000 1408.0 1408.0

F000’ 1409.65

h,k,lmax 13,29,15 13,29,15

Nref 6270 6198

Tmin,Tmax 0.924,0.941 0.544,1.000

Tmin’ 0.847

Correction method= # Reported T Limits: Tmin=0.544 Tmax=1.000

AbsCorr = MULTI-SCAN

Data completeness= 0.989 Theta(max)= 26.000

R(reflections)= 0.0494(4118) wR2(reflections)= 0.1334(6198)

S = 0.916 Npar= 435

---

6. References

1. Armarego, W. L. F.; Chai, C. L. L. *Purification of Laboratory Chemicals* (7th *ed.*). Elsevier Science: Oxford (UK) and Waltham (USA), **2013.**

2. Fang, Y. S.; Rogness, D. C.; Larock, R. C.; Shi, F. *J. Org. Chem.* **2012**, *77*, 6262-6270. DOI:10.1021/jo3011073.

3. Lee, H.; Suzuki, M.; Cui, J.; Kozmin, S. *J. Org. Chem*. **2010**, *75*, 1756-1759 DOI: 10.1021/jo9025447.
